# Mobilisation of Hematopoietic CD34^+^ Precursor Cells in Patients with Acute Stroke Is Safe - Results of an Open-Labeled Non Randomized Phase I/II Trial

**DOI:** 10.1371/journal.pone.0023099

**Published:** 2011-08-26

**Authors:** Sandra Boy, Sophie Sauerbruch, Mathias Kraemer, Thorsten Schormann, Felix Schlachetzki, Gerhard Schuierer, Ralph Luerding, Burkhard Hennemann, Evelyn Orso, Andreas Dabringhaus, Jürgen Winkler, Ulrich Bogdahn, for the RAIS (Regeneration in Acute Ischemic Stroke) Study Group

**Affiliations:** 1 Department of Neurology, University of Regensburg, Bezirksklinikum Regensburg, Regensburg, Germany; 2 Neurological Therapy Center and St. Marien Hospital, Köln, Germany; 3 Department of Neurology, University Medical Centre, Düsseldorf, Germany; 4 Institute of Neuroradiology, Regensburg University Medical Centre, District Medical Centre Regensburg, Regensburg, Germany; 5 Department of Hemato-Oncology, Regensburg University Medical Centre, Regensburg, Germany; 6 Department of Clinical Chemistry, Regensburg University Medical Centre, Regensburg, Germany; 7 St. Mauritius Therapy Clinic, Meerbusch, Germany; Julius-Maximilians-Universität Würzburg, Germany

## Abstract

**Background:**

Regenerative strategies in the treatment of acute stroke may have great potential. Hematopoietic growth factors mobilize hematopoietic stem cells and may convey neuroprotective effects. We examined the safety, potential functional and structural changes, and CD34^+^ cell–mobilization characteristics of G-CSF treatment in patients with acute ischemic stroke.

**Methods and Results:**

Three cohorts of patients (8, 6, and 6 patients per cohort) were treated subcutaneously with 2.5, 5, or 10 µg/kg body weight rhG-CSF for 5 consecutive days within 12 hrs of onset of acute stroke. Standard treatment included IV thrombolysis. Safety monitoring consisted of obtaining standardized clinical assessment scores, monitoring of CD34^+^ stem cells, blood chemistry, serial neuroradiology, and neuropsychology. Voxel-guided morphometry (VGM) enabled an assessment of changes in the patients' structural parenchyma. 20 patients (mean age 55 yrs) were enrolled in this study, 5 of whom received routine thrombolytic therapy with r-tPA. G-CSF treatment was discontinued in 4 patients because of unrelated adverse events. Mobilization of CD34^+^ cells was observed with no concomitant changes in blood chemistry, except for an increase in the leukocyte count up to 75,500/µl. Neuroradiological and neuropsychological follow-up studies did not disclose any specific G-CSF toxicity. VGM findings indicated substantial atrophy of related hemispheres, a substantial increase in the CSF space, and a localized increase in parenchyma within the ischemic area in 2 patients.

**Conclusions:**

We demonstrate a good safety profile for daily administration of G-CSF when begun within 12 hours after onset of ischemic stroke and, in part in combination with routine IV thrombolysis. Additional analyses using VGM and a battery of neuropsychological tests indicated a positive functional and potentially structural effect of G-CSF treatment in some of our patients.

**Trial Registration:**

German Clinical Trial Register DRKS 00000723

## Introduction

Cerebrovascular disorders, specifically ischemic stroke, remain the third most common cause of death and a leading cause of disability [1], their significance steadily increasing due to demographic changes in western industrial societies. The introduction of intravenous (IV) thrombolysis with recombinant tissue plasminogen activator (rtPA) more than a decade ago was a milestone in stroke therapy; however, only a minority of patients benefit from this treatment due to the narrow time window of its effectiveness [2], [3], [4], [5]. Although a number of treatment targets within the cascade of neuronal death have been identified, neuroprotective strategies so far have proved to be a constant source of disappointment [6], [7].

Despite these setbacks, neuroregeneration, neuroplasticity and neuronal repair still may have the potential to improve functional and structural outcome once normal cerebral blood flow has been reestablished, if the prior ischemic environment favors repair. Similar to other organs, endogenous stem cells and progenitor cells are already present in the brai, mainly in the hippocampus and the subventricular zone [8], [9]. In addition, hematopoietic stem cells appear to be mobilized by the cerebral ischemic event and have the potential to home in on damaged brain parenchyma [10].

Since neural stem cells and precursor cells are the most hypoxia-resistant cells [11], they may survive the critical hypoxic phase of ischemia. These pluripotent cells have the capacity to differentiate into neurons, astrocytes, oligodendrocytes, and endothelial cells. Reports on different experimental stroke models indicate that stem cells can survive, integrate, and even operate as neurons [12], [13], [14].

Granulocyte colony-stimulating factor (G-CSF) is a 19.6-kDa glycoprotein that regulates the generation, proliferation, survival, and maturation of neutrophilic granulocytes [15]. G-CSF acts via the G-CSF receptor (G-CSFR), a single transmembrane protein belonging to the hematopoietin receptor superfamily, and is expressed on a variety of hematopoietic and neuronal cell types [16]. Its expression in the brain has been described in the cortex [16], [17], hippocampus, and subventricular zone among others (cerebellum and brainstem nuclei) [17], [18]. G-CSF has been used extensively in the last decades to mobilize CD34^+^ hematopoietic stem cells in neutropenic patients and for reconstitution of bone marrow [19], [20]. It has been shown to be safe with only a few well-described side effects.

Over the last few years evidence has emerged that G-CSF has a therapeutic potential in stroke. It has been shown to exhibit neuroprotective and regenerative activity in experimental stroke models [16], [21], [22], [23], [24], [25]. Initial clinical phase II studies on the use of systemically administered G-CSF in patients have also shown promising results [26], [27]. However, the timing, route of application, dosing, and length of G-CSF treatment have not yet been thoroughly investigated.

We report the results of an open-label acute ischemic-stroke phase I/II trial centered on the use of 5 daily subcutaneous injections of human recombinant G-CSF to mobilize CD34^+^ cells in a dose-escalation trial in acute stroke patients. Secondary outcome parameters included neuropsychological testing and voxel-guided morphometry (VGM). As an extension to previous studies, IV thrombolysis in acute stroke was allowed, adding to the safety profile of this approach.

## Methods

The protocol for this trial and supporting CONSORT checklist are available as supporting information; see Checklist S1 and Protocol S1.

### Treatment

The study was approved by the local institutional review board in accordance with the guidelines of Helsinki, and written informed consent was obtained from each patient. All amendments to the original protocol were approved by the local ethics committee and are attached as Amendment S1. Changes included the method of VGM for analysis of the acquired MRI data sets, additional MRI follow-up in selected patients after 5 years, and a higher leucocyte count at which a dose reduction in GCF-F would be initiated (described in this paper).

The trial is registered at the German Clinical Trial Register with the number DRKS 00000723.

GCS-F (Neupogen®, Amgen, Munich) was administered as a subcutaneous injection over a 5-day-period in a dose-escalation design. The initial daily dose of G-CSF was 2.5 µg/kg(bodyweight, bw) in the first 8 patients; this dose was escalated to 5 µg/kg(bw) in 6 patients and again to a final dose of 10 µg/kg(bw) in the last 6 patients. The study drug was administered within 12 hrs after stroke onset. Subsequent doses were given at 24-hr intervals. In agreement with the predefined safety parameters of the initial study protocol, the G-CSF dose was tapered from 2.5 µg to 1.25 µg (a 50% dose reduction) in cases in which leukocyte counts exceeded 20,000/µl. After the first 8 patients displayed no adverse events due to leukocytosis, the protocol was amended to state that a dose reduction should occur at a leukocyte threshold >50,000/µl.

### Inclusion and exclusion criteria

Patients suffering from acute middle cerebral artery ischemic stroke and moderate neurological deficits (National Institute of Health Stroke Scale [NIHSS] Score 4 to 22) were the target population in this study. Patients were enrolled in the study within 12 hrs after onset of symptoms (Table 1).

**Table 1:** Inclusion and exclusion criteria.

Inclusion criteria	Exclusion criteria
moderate acute stroke within MCA (middle cerebral artery) territory, specified as M2-occlusion dominant and non-dominant hemispheres patient must comprehend the study protocol inclusion in study protocol within 12 hours after stroke onset age ≥18 years to ≤65 years NIHSS Score 4–22 adequate liver function: GOT, GPT, γGT<3 times upper normal values; bilirubin <1.5 mg/dl adequate bone marrow function (no gross abnormalities in thrombocytes or leukocytes) patient must be compliant patient provided written informed consent	previous treatment with Abciximab (Rheopro**®**) thrombolytic therapy within previous 2 weeks except acute rTPA treatment for stroke participation in another trial global aphasia any type of immediately necessary intervention, e.g. carotid endarterectomy ulcerating plaque of carotid artery or pseudoocclusion signs and symptoms of acute cerebral vasculitis dissection of brain arteries relevant for acute symptomatology previous disabling stroke events (ischemic or hemorrhagic stroke) systemic malignancy hematological system disorder (e.g. myeloproliferative disorder) thrombocyte function disorders metabolic syndrome with inadequate treatment parameters, e.g. excessive hypertension, hyperlipemia, hyperglycemia known deficit in hemostasis serious coronary heart disease sickle cell anemia allergy against G-CSF (Neupogen **®**) pregnancy heavy smoker, daily use ≥20 cigarettes immunosuppressive medication (e.g. glucocorticoids) any other serious disease, for example: severe psychiatric disorder (major depression, schizophrenic psychosis, addiction), severe cardiac disorder with hemodynamic relevance), positive HIV serology

### Standard stroke care/thrombolytic therapy

Full standard stroke care was given to every patient. Concomitant treatments included fluid supplement, antiplatelet or anticoagulant medications, antibiotics, antihypertension drugs, fever control, and insulin when medically indicated. Thrombolytic therapy with standard IV rtPA (0.9 mg/kg bw) was initiated within 3 hours of stroke onset, in accordance with international guidelines.

### Safety - clinical parameters

The degree of each patient's neurological deficit(s) was assessed using the National Institute of Health Stroke Scale (NIHSS), the Barthel Index (BI), and the modified Rankin Scale (mRS) at study inclusion. The BI and mRS assessments were repeated at 24 hours and on Days 7, 28 and 90. In addition, NIHSS scores were recorded within 24 hours and daily up to Day 7. Significant deterioration leading to adverse events was defined as decreases from baseline of 4 points in the NIHSS score, 10 points in the BI, and 2 points in the mRS.

Attention was directed to thrombotic complications such as pulmonary embolism, deep venous thrombosis, and cerebral venous thrombosis. General vital parameters (heart rate, blood pressure, and temperature) were routinely monitored and included in the primary safety analysis.

### Clinical laboratory parameters

Clinical chemistry monitoring, including tests of blood count, C-reactive protein (CRP), electrolytes, liver enzymes, renal function, lipids, and coagulation parameters, was performed on Days 1 through 7 and on Days 14, 28, and 90. Significant toxicity was assumed when liver enzymes increased 3-fold over standard values, renal clearance decreased to 50% of baseline, and a decrease in the number of platelets and/or red blood cells reached below 50% of baseline. Quantitative determination of circulating hematopoietic stem- and progenitor cells (CD 34^+^) was conducted on Days 1, 4, 7, 28, and 90 according to the modified ISHAGE guidelines [28] by using a single-platform no-wash technique: two technical modifications were used (PharMingen, San Diego, USA; Becton Dickinson, Heidelberg, resp. Beckman-Coulter, Krefeld, Germany).

### Neuroradiological investigations

Brain hemorrhage was disclosed using standard cerebral computed tomography (cCT) prior to study inclusion. Magnetic resonance imaging (MRI) was performed within 48 hrs after admission and on Days 7 and 90 (2 patients underwent additional late follow-up investigations). The following MR sequences were used: T1- and T2-weighted, fluid-attenuated inversion recovery (FLAIR), diffusion-weighted MRI (DWI), apparent diffusion coefficient (ADC), high-resolution sagittal MPRAGE-T1, and time-of-flight magnetic resonance angiography (TOF-MRA). Adverse events were suspected if any of the following were found: significant and/or unexpected enlargement of the ischemic area, recurrent vascular occlusion, prolonged edema, atypical reperfusion, or any type of CNS hemorrhage except minor hemorrhagic transformation.

### Neuropsychological assessment

All evaluable patients underwent a battery of neuropsychological tests on Day 7; a subpopulation of eight patients underwent additional tests on Day 90. The tests were performed to assess verbal and nonverbal intelligence, long-term memory and working memory, and attention and word fluency. Details of the test battery can be found in another publication [29].

### Voxel-guided morphometry (VGM)

The volumetric method used in this study was voxel-guided morphometry (VGM), the details of which have been described elsewhere [30]. In principle, a combination of linear and nonlinear transformation processes is used to register consecutive 3D data sets from 2 or more time points in the same individual. This allows the clinician to follow individual structural changes within the brain volume on a voxel-by-voxel basis. VGM from high-resolution MPRage datasets consists of four steps. The first step is a coarse linear alignment by the extended principle axes theory (ePAT) generalized to affine movements; the second, a cross-correlation–based technique involving a matrix norm for fine linear alignment; and the third, an applied high-dimensional multiresolution full-multigrid method used to determine nonlinear deformations, thereby achieving a complete exploitation of information and effective processing. This method is used to measure a gray value–guided movement of each voxel from source to target. The resulting high-dimensional deformation field is further processed using a fourth step, the determination of volume alterations for each voxel. In a subpopulation of evaluable patients (N = 8, 3 female, 5 male) VGM was performed (table S1). The main endpoint criteria here were defined as any change in gray matter, white matter, or cerebral spinal fluid (CSF) space, expressed as a percentage change in the individual patient. In 2 patients long-term follow-up was performed after 5 years (Patients 12 and 13) because volume increases in the infarct area were observed after 3 months.

### Statistical methods

All patients who received the starting dose of G-CSF were included in the analysis and regarded as the intention-to-treat population. For the final data analysis, however, only patients whose treatment corresponded to the protocol were included in our evaluation. In this open-label, single-arm dose-escalation safety trial we chose a descriptive analysis: data are presented as medians and all analyses were performed using SPSS software (version 16.0; 2007 SPSS Inc.).

Neuropsychological raw data were transformed into z-scores to transform data into normative data (related to confounders); the definition of cut-offs for significant impairment is described elsewhere[31]. Since only eight patients completed both neuropsychological assessments, Wilcoxon nonparametric tests for comparisons of the transformed scores were computed.

## Results

### Patients

Twenty consecutive patients (ages 32–65 yrs, mean 55 yrs; 13 male, 7 female) were enrolled. Five patients received standard rtPA treatment prior to study inclusion. Two patients were lost to follow-up after Day 28 because they withdrew their consent to participate in the study. The baseline vascular risk profiles of the three dose groups are presented in Table 2. All 20 patients presented with signs and symptoms of middle cerebral artery (MCA) infarction. MRI at 48 hrs confirmed cerebral ischemia within the MCA territory in 17 patients, within the anterior cerebral artery (ACA) territory in 1 patient (No. 13), and within the vertebrobasilar territory in 2 patients (Patients 4 and 8). The latter were regarded as minor protocol violations and not excluded. In accordance with the study protocol, the dose of the study drug was reduced from 2.5 µg to 1.25 µg in three patients in whom the leukocyte count was >20,000/µl. After inclusion of 8 patients with high leukocyte counts but no adverse events, the protocol was amended to a G-CSF dose reduction whenever the leukocyte count exceeded 50,000/µl. None of the subsequent patients met this criterion. In an additional patient the dose was reduced from 10 µg to 5 µg because of another protocol violation.

**Table 2:** Baseline characteristics of intention-to-treat population.

Patient No.	Sex	Age, yr	Location			Lysis		MRS	Stroke risk factors			
			ACA	MCA	VBT	Yes	no	Pre-stroke	Smoking	Hypertension	Diabetes	Lipids
G-CSF Dose 2.5 µg/kg (BW)												
1	M	59		+			+	0	−	+	−	−
2	M	57		+			+	0	−	−	−	−
3	M	56		+			+	0	−	−	−	−
4	M	57			+		+	0	+	+	+	−
5	F	55		+		+		0	−	−	−	−
6	F	54		+		+		0	−	+	−	−
7	F	61		+			+	0	−	+	−	−
8	M	65			+		+	0	+	−	−	−
G-CSF Dose 5 µg/kg (BW)												
9	F	32		+			+	0	−	−	−	−
10	M	54		+			+	0	+	+	+	+
11	M	64		+			+	0	−	+	−	−
12	F	46		+			+	0	−	−	−	−
13	F	55	+				+	0	+	+	−	+
14	M	52		+		+		0	−	+	−	−
G-CSF Dose 10 µg/kg (BW)												
15	M	53		+			+	0	+	−	−	+
16	M	59		+			+	0	−	+	−	−
17	M	64		+			+	0	−	+	−	−
18	M	62		+		+		0	−	+	−	−
19	M	64		+		+		0	−	+	−	−
20	F	35		+			+	0	−	+	−	−

**Note: M = male; F = female; BW = Body weight ; ACA = anterior cerebral artery, MCA = middle cerebral artery, VBT = vertebrobasilar territory; MRS = modified Rankin scale.**

### Primary endpoint safety

**Figure 1:**
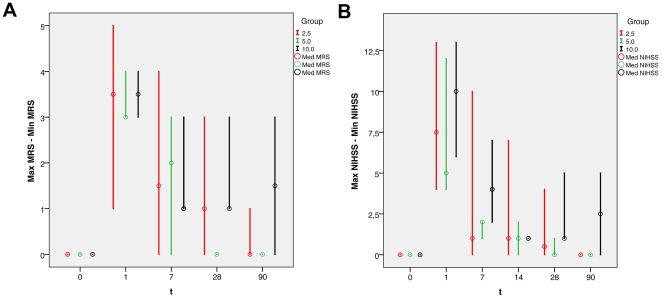
Scores over time. MRS (Modified Rankin Scale) scores over time as median with minimum and maximum in three different dosage groups (A). NIHSS (National Institutes of Health Stroke Scale) scores over time as median with minimum and maximum in three different dosage groups (B).

A total of 4 patients (20%) experienced adverse events resulting in termination of study drug treatment (Table 3). The remaining 16 patients (10 males/6 females) were determined to be the evaluable population and were included in the final analysis for study endpoints. Moderate thrombocytopenia without clinical symptoms (platelet count <150,000/µl) occurred in two patients. A single patient was included with a baseline platelet count of 131,000/µl (a minor violation), but had an unremarkable follow-up. Vital parameters—heart rate, blood pressure, and temperature—in all patients remained within normal limits. None of the patients complained of pain. During G-CSF treatment and the follow-up period none of the remaining patients experienced a neurological deterioration, as assessed using the NIHSS, BI, and mRS. All outcome scales indicated a good outcome at all time points, as demonstrated in Figure 1A for the mRS and Figure 1B for the NIHSS. All laboratory endpoints, including platelet and erythrocyte counts, coagulation, and liver and renal parameters, remained stable throughout the 90-day observation period. No increase in infarct volumes was seen on serial MRIs between 48 hrs and 7 days, and no adverse findings were seen up to Day 90. In one patient, baseline MRI showed a middle cerebral artery infarction in FLAIR and DWI images, but MRI on Day 90 did not reveal any remaining lesion in FLAIR, T1-weighted, or T2-weighted images.

**Table 3:** Serious adverse events with time of onset, duration and outcome; ICP = intracranial pressure; ESR = erythrocyte sedimentation rate.

SAE Term	Patient No.	Date of onset	Number of G-CSF doses received	Duration (days)	Relationship	Symptoms/AEs associated with SAE	Patient follow-up
Intracranial hemorrhage 3 hrs after first s.c. injection of 5 µg/kg G-CSF, 12 hrs after ischemia; patient was treated with iv heparin with ptt 44 secStudy medication withdrawn.	11	11 Jan 2004	1	17	Unlikely	Nausea, vomiting, and hemiplegia	Repeated hemorrhages without definite diagnosis of coagulation abnormality or thrombocyte dysfunction
Increasing edema in the area of infarction with rise of intracranial pressure about 20 hrs after fourth s. c. injection of 10 µg/kg G-CSF, about 110–120 hrs after ischemia	16	07 Jul 2005	4	10	Unlikely	First nausea, vomiting and increasing hemiparesis. Intubation and application of ICP sensor on 07/07/2005. Pneumonia with increase of CRP under ventilation.	Stabilization under anti-edematous and antibiotic therapy. Extubation after five days.
Pectanginous symptomatology about 12 hrs after second s.c. injection of 5 µg/kg G-CSF, about 48 hrs after ischemia; study medication withdrawn	17	08 Nov 2006	2	1	Unlikely	No	Controls of cardiac enzymes and ECG revealed no pathology.
Suspected endocarditis. Strongly accelerated ESR 10 hrs after first s.c. injection of 5 µg/kg G-CSF, about 21 hrs after ischemia Echocardiography showed moderate mitral regurgitation with thickening of leaflets; these results raised suspicion of Libman-Sacks endocarditis. Study medication withdrawn.	20	04 Sep 2008	1	1	Unlikely	Fatigue	Controls of ESR the next days showed normalized values. Further diagnostics revealed no autoimmune disease

### CD34^+^ stem cell/progenitor mobilization

**Figure 2:**
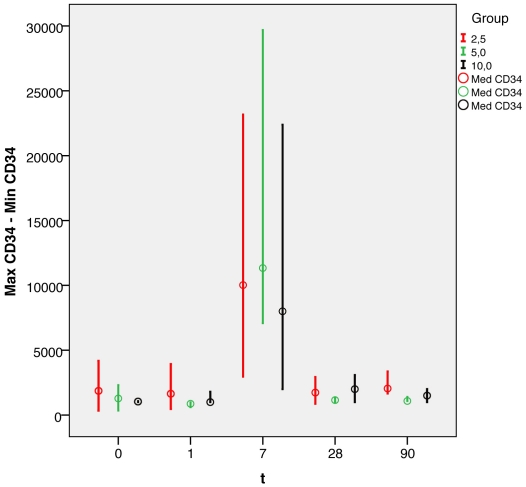
Absolute CD34+ cell count/µl over time as median with minimum and maximum over time in the three different dosage groups.

The number of CD34^+^ cells measured on Days 0, 1, 7, 28, and 90 increased from a median of 1653 cells/ml at baseline to a median of 10,022 cells/ml on Day 7. No correlation was found between the G-CSF dose and the mean CD34^+^ cell count; Fig. 2). Total leukocyte counts (figure S2) also reflected successful mobilization without an increase in inflammatory parameters such as CRP (figure S1).

### Voxel-guided morphometry

**Figure 3:**
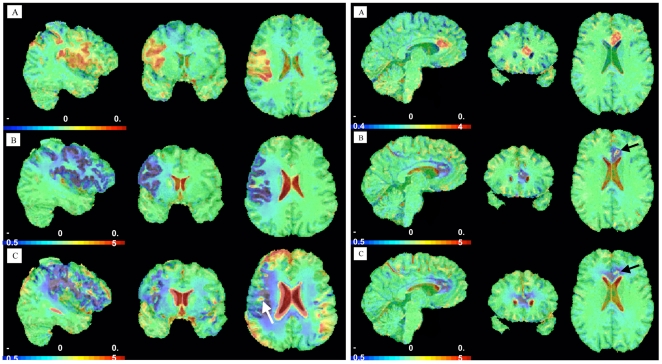
VGM volume fields in Patients 12 (left) and 13 (right). Left: The first MRI, obtained within 48 hours after stroke onset, is compared to MRIs obtained 4 days (A), 3 months (B), and 5 years (C) later. After 5 years, volume increases can be detected in a spotlike distribution (arrow) within the infarcted area. Right: Volume fields are shown from MRIs obtained 5 days (A), 3 months (B), and 5 years after the first MRI had been obtained immediately after stroke onset. A small area of volume increase (arrow) within the original ischemic zone, first seen after 3 months, still persists.

In almost all patients a slight volume increase within the ischemic area was observed within the initial time frame (Day 1 to Day 7); this was attributed to post-infarct edema. In most patients atrophic changes in the gray matter of the affected side between Days 1 and 90 exceeded what we expected based on our analysis of the T1-weighted images. Interestingly, in 2 patients (Patients 12 and 13) volume changes indicated some localized increases within infarcted and atrophied areas. These patients underwent long-term follow-up, which revealed persistent increases in the localized volumes within ischemic areas. Individual anatomical MR images obtained in the same patient were superimposed on previous images to demonstrate the anatomical areas involved in this structural change. After segmentation and linear alignment, the slices were carefully arranged so that they could be compared precisely on a voxel-by-voxel basis. This is shown in 2D in this paper, but full 3D images were also prepared for the whole brain volume, thus avoiding any interactive definition of regions of interest (see Figure 3 a and b).

Volume change was quantified (see LUT below the sagittal slices) locally. The LUT presents local volume changes in percentages (−0.2 = 20% volume decrease, 0.2 = 20% volume increase). Green and blue regions represent volume reductions, whereas yellow and red regions represent volume increases. In Patient 12 after 3 months a small spot of volume augmentation could be observed within the infarct area. After 5 years volume increases could be detected in the patient's frontal and parietal areas as well as in a spottled distribution (arrow) within the infarcted area ipsilaterally and in temporoparietal areas in the contralateral hemisphere (Figure 3). In Patient 13, who suffered an anterior cerebral artery infarction in the left hemisphere that also involved the corpus callosum, volume augmentation within the ischemic area—probably due to edema development—could be observed after 5 days. After 3 months, a loss of volume could be observed within the ischemic area accompanied by an increase in the volume of the lateral ventricle. An area of volume increase within the ischemic zone was seen. After 5 years, the frontal volume loss spread beyond the infarcted area and included adjacent white matter ipsi- and contralaterally. A slight volume decrease was also observed in the posterior white matter and in cortical areas in both hemispheres. The small area of volume increase (arrow) within the original ischemic zone still persisted (Figure 3).

### Neurocognitive findings

Data in 8 of 16 patients (mean age 56.3±7.1 years) could be analyzed for follow-up between Days 7 and 90. In the initial assessment of the group the mean scores in working memory (digit span backward, mean z = −1), attention (Trail Making Test B, mean z = −1.3), and visual perception (Rey Complex Figure Copy, mean z = −1.5) were significantly lower than normative data. In the second assessment on Day 90, only visual perception had a mean score lower than 1 standard deviation (Rey Complex Figure Copy mean z = −1.5). Attention and working memory were no longer impaired for the entire group. In the nonparametric comparisons there was a significant improvement in verbal (Wechsler Logical Memory II; Mt7 = −0.4, Mt90 = 0.3, p<0.05) and nonverbal (Rey Complex Figure Delay, Mt7 = −0.4, Mt90 = 0.3, p<0.05) long-term memory, as well as in attention (Trail Making Test B, Mt7 = −0.4, Mt90 = 0.3, p<0.05). No significant deterioration in neurocognitive functions was identified in the nonparametric comparison of Day 90 with Day 7. Details are shown in Table 4.

**Table 4:** Neuropsychological assessment.

Differences between Day 7 and Day 90 in standard deviations										
Patient No.	2	3	8	10	12	13	18	19	Mean difference	statistical significance
Long-term memory										
Wechsler Logical Memory II	0.7	1.4	−0.3	0.8	2	1.2	0.3	0	0.8	p<0.05
Wechsler Logical Memory I	0.8	1	−0.5	1.4	1.5	1.5	0.8	−0.1	0.6	not significant
Rey Complex figure Delay	0.5	1.5	0.8	−0.5	2.2	0.9	0.5	−0.1	0.7	p<0.05
Working memory										
Digit span backward	0	0.8	0	0.7	0.7	0	0	0	0.4	not significant
Digit span forward	0	1.8	−0.9	−0.9	1.7	1.7	0	0.9	0.3	not significant
Block span	2	0	0	1	1	2	0	−2.9	0	not significant
Attention										
Trail making Test B	1	0.8	0	1.6	1.7	1.5	−0.2	0	0.8	p<0.05
Ruff 2&7	0.2	0.4	0.4	0.4	0.2	n.a.	1.1	−0.7	0.5	not significant
Lexical Fluency	0.8	−0.1	0	1	1	0.8	0.2	−0.5	0.4	not significant
Semantic fluency	0.4	2.2	1.8	−0.7	0.3	0.4	0.1	−0.6	0.6	not significant
Visual perception										
Rey Complex Figure Copy	1.4	0.3	0	0.5	0.3	1.4	0.8	−0.3	0.2	not significant
Trail Making Test A	−0.4	0.7	0.3	0	0.9	−0.4	1.3	0.4	0.4	not significant
Full scale IQ	−0.8	0.2	0.4	1.6	0.8	0.3	0.5	−0.5	0.3	not significant

Negative values used to describe a loss from Day 7 to Day 90; positive values to describe an improvement. A difference in z-scores under or above z = ±1 is bigger than one standard deviation.

n.a. = not administered. Significance computed using the Wilcoxon test for repeated measures.

## Discussion

This is the first phase I/IIa trial in which standard IV thrombolysis and daily administration of subcutaneous G-CSF in a dose-escalating schedule were begun within the first 12 hours of acute ischemic stroke, thus combining recanalization strategies with a potentially regenerative treatment approach. Overall, the study results confirm the safety and feasibility of administering subcutaneous G-CSF during the acute stage of ischemic stroke. A clear dose-response relationship between G-CSF administration and CD34^+^ stem cell mobilization could not be established in this rather small cohort. Median mobilization levels of up to 10×10**^3^** cells/µl and peak levels of up to 29×10**^3^** cells/µl were achieved. Adverse events occurred in 4 patients, but they were regarded as unrelated to the G-CSF treatment. Good clinical outcome parameters and neurocognitive functions indicated safety and good tolerance, as well as no functional impairment. The VGM data revealed substantial atrophy around the infarcted territory; in two cases additive localized gray matter within the infarct lesions was seen.

In a recent study Schäbitz et al. assessed high intravenous doses of G-CSF in patients with acute stroke [32]. Major differences between that study and ours include the mode of drug administration (intravenous vs. subcutaneous), length of drug therapy (3 days vs. 5 days), and the fact that in our study tPA therapy was allowed. Given our choice of subcutaneous rather than intravenous administration, the systemic dose of G-CSF in our trial was probably lower but perhaps induced longer-term mobilization of hematopoietic stem cells.

### Mobilization of CD34^+^ cells and safety

Augmented levels of G-CSF have been observed in a variety of instances such as physical exercise [33], [34], septic exposure to various lipopolysaccharides [35], and exposure to lipoteichoic acid [36], as well as in different types of ischemia [37]–[38]. G-CSF transcripts are induced 65-fold at 16 hrs in experimental middle cerebral artery occlusion [39]: it was concluded that G-CSF induction in the brain may be part of an intrinsic stress response aimed at neuroprotection. Since acute stroke is accompanied by phases of breakdown of the blood-brain barrier, G-CSF could pass into the systemic circulation and mobilize hematopoietic cells. The hypothesis of an invasion of these cells into the injured tissue and a contribution to the initiation of cerebral repair seems tempting. Indeed, mobilized G-CSF peripheral progenitor cells administered intravenously have led to functional recovery in a rodent stroke model [40]. Intravenous injection of CD34^+^ cells 48 hrs after experimental brain ischemia reduced lesion size, enhanced angiogenesis, and neurogenesis, and improved functional outcome. It must be noted that angiogenesis and recanalization provide a favorable environment for neuronal regeneration: when CD 34^+^ cell-induced angiogenesis was blocked, neurogenesis was reduced [41]–[42]; this finding underlines the probable importance of CD34^+^ cell lesion access by recanalization therapy and the potential relevance of the stem cell niche. In CD34^+^-transplanted mice hematopoietic cells have been shown to differentiate into microglial and perivascular cells after middle cerebral artery occlusion; under these conditions a few cells were detected by positive staining of the neuronal marker NeuN [43]. On the other hand, neuronal and oligodendroglial gene products were detected in CD34^+^ cells derived from murine bone marrow, with their expression regulated in the brain [44].

In human adults CD34^+^ cells may differentiate into both hematopoietic stem cells and endothelial progenitor cells [45], [46]. Increasing evidence shows that circulating CD34^+^ cells contribute to angiogenesis after brain infarction [47]. Therefore it seemed reasonable to mobilize CD34^+^ cells in cases of acute cerebral infarction to foster potential repair processes—neuroprotective or even neuroregenerative. Translation of preclinical stroke research, especially that done in rodents, is a tall order, and recent data suggest multiple mechanisms of stem cell therapy for ischemic stroke [48], [49]. Dirk Hermann and his group recently suggested multiple bystander effects of transplanted neuronal precursor cells, such as reduction of inflammation, glial scar formation, and neuronal apoptotic death contributing to functional recovery in mice [50]. However, we cannot exclude true neuronal differentiation following bone-marrow stem-cell mobilization, and the complex mechanism in the injured brain that drives stem cells into the neuronal or astrocytic lineage is not completely understood [51]. Yet, the fate and function of hematopoietic or neuronal stem cells in the much more complex human brain will be a new challenge in stroke research and may be enhanced by techniques such as VGM and fMRI.

We found that subcutaneous administration of G-CSF was effective in mobilizing CD34^+^ stem cells into the peripheral blood in patients with acute stroke. Rather high CD34^+^ cell counts were obtained in the peripheral circulation, indicating enough cells for potential treatment effects. CD34^+^ cell mobilization varied within and among the different dosage groups; we were unable to establish a clear-cut dose-response relation, probably due to a high individual variation within small patient cohorts.

Our results in part contrast with those of the study by Sprigg and coworkers. In their randomized, controlled trial they assessed the safety of G-CSF administered for 5 days between Days 7 and 30 after stroke and the effect on circulating CD34^+^ stem cells. Those authors detected an increase in CD34^+^ cells in a dose-dependent manner [27]. The investigators started treatment at the earliest time, 7 days after symptom onset, in order not to interfere with leukocytosis seen during acute stroke: G-CSF-induced leukocytosis might increase the risk of leukocyte plugging of the microvasculature [52]. Our data indicate that high leukocyte counts in the initial phase do not seem to be associated with microembolism or sludging, as confirmed by findings of another randomized trial [26]. Specifically, new lesions on T2-weighted MR images were excluded, indicating an absence of obvious or silent additional embolic events. For leukocyte counts see figure S2. In a recent trial we were able to show that stroke leads to spontaneous increases in serum G-CSF and CD34^+^ cells [10]. Additional G-CSF may lead to an interference of spontaneous and induced G-CSF levels, and add to the effects of individual quality and quantity of mobilization at different time points. This may confound dose-response relationships in G-CSF trials. Moreover, our study is the first to indicate that the co-administration of G-CSF and rtPA is safe in patients with acute stroke. Five of our 20 patients received intravenous rtPA and none experienced intracerebral hemorrhage or other events relevant to safety issues. Clearly safety is an issue that needs further investigation.

Adverse events occurred in four of our remaining patients. A relationship between adverse effects and the study drug was unlikely in all cases. In one patient intracranial hemorrhage occurred 3 hrs after study drug administration and 12 hours after stroke onset. Laboratory results showed no thrombocytopenia or thrombocyte dysfunction but the patient was treated with IV heparin because cardioembolic stroke was suspected. A relationship between intracranial hemorrhage and G-CSF treatment seemed unlikely, but the use of anticoagulants in addition to G-CSF treatment should be carefully investigated in future trials.

Respiratory infections are a major contributor to morbidity following ischemic stroke; however, in our study treatment was not associated with an additional risk of infection. For CRP counts see figure S1. One patient developed pneumonia 1 day after cessation of G-CSF administration due to aspiration but a causal effect of G-CSF is very unlikely. In addition, a double-blind, placebo-controlled study showed safe but not efficacious administration of G-CSF in patients with pneumonia and severe sepsis [53]. In our study both suspected cardiac side effects were regarded to be completely unrelated (Table 3). Other systemic side effects, specifically bone pain or headaches, were not observed.

### Outcome parameters

Good functional outcomes were obtained in all of our patients. MRI investigations in our study displayed the expected structural changes after stroke. Interestingly, in one patient MRI obtained on Day 90 showed no remaining lesion on FLAIR and T1-weighted sequences. In addition we analyzed intra-individual brain volume changes over time using VGM [30], [54]. This is in line with a study by Kraemer et al., who showed secondary brain atrophy after stroke of the middle cerebral artery territory in 10 patients. Interestingly, and in contrast to our study, those authors did not detect any volume increase in either the acute or chronic state [54]. In 2 patients we were able to delineate a small volume increase in the infarct area (Figure 3). The interpretation of these findings clearly requires further investigations.

Due to the study design (phase I/II dose-escalation) and the small number of patients, additional subgroup analyses of outcomes were not performed, These should be addressed in further trials.

To our knowledge to date there is no information about the effects of G-CSF on cognition in stroke patients. Gibson et al. showed a beneficial effect of a single dose of G-CSF on cognitive deficits in transient focal ischemia in mice [22]. Our study is the first to describe neuropsychological findings in G-CSF-treated patients in acute stroke. The comparison of neurocognitive performance on Days 7 and 90 showed significant improvements in long-term memory and attention. There was no significant loss of function, so treatment with G-CSF at least does not seem to have adverse effects on neurocognitive function.

### Conclusion

The results of our study demonstrate a good safety profile for daily G-CSF injections when begun within 12 hours of acute stroke, even in combination with IV thrombolysis. Additional analyses involving voxel-guided morphometry and a battery of neuropsychological tests in some of our patients hint toward a positive functional regenerative effect of G-CSF treatment. Further studies should focus on the time window within which G-CSF treatment should commence, the length of this treatment, and on dose-response and structural effects.

## Supporting Information

Figure S1 **CRP (c-reactive protein) in mg/dl over time as median with minimum and maximum in the three different dosage groups.**
(TIF)

Figure S2 **Leukocytes/µl over time as median with minimum and maximum in the three different dosage groups.**
(TIF)

Table S1 **Patients included in the VGM analysis. The table shows the 8 individuals' gender and age and the timing of sequential MRIs after stroke onset (first to third MRI in days, fourth MRI in months).**
(DOC)

Checklist S1 **CONSORT 2010 checklist of information to include when reporting a randomised trial.**
(DOC)

Protocol S1 **Regeneration in Acute Ischemic Stroke (RAIS) [German: Regeneration nach akutem ischämischen Schlaganfall — Einarmige, Dosis-eskalierte klin. Phase I/II - Studie zur hämato-poetischen Stammzell-Mobilisation bei akutem ischämischen A. cerebri media –Teilinfarkt].**
(PDF)

Amendment S1 **Amendments to the original protocol.**
(PDF)
