# Supplementary figures and images for: Mobilisation of Hematopoietic CD34+ Precursor Cells in Patients with Acute Stroke Is Safe - Results of an Open-Labeled Non Randomized Phase I/II Trial
Source: PLoS One. 2011 Aug 26;6(8):e23099. doi: 10.1371/journal.pone.0023099 (PMC3162562; doi:10.1371/journal.pone.0023099)

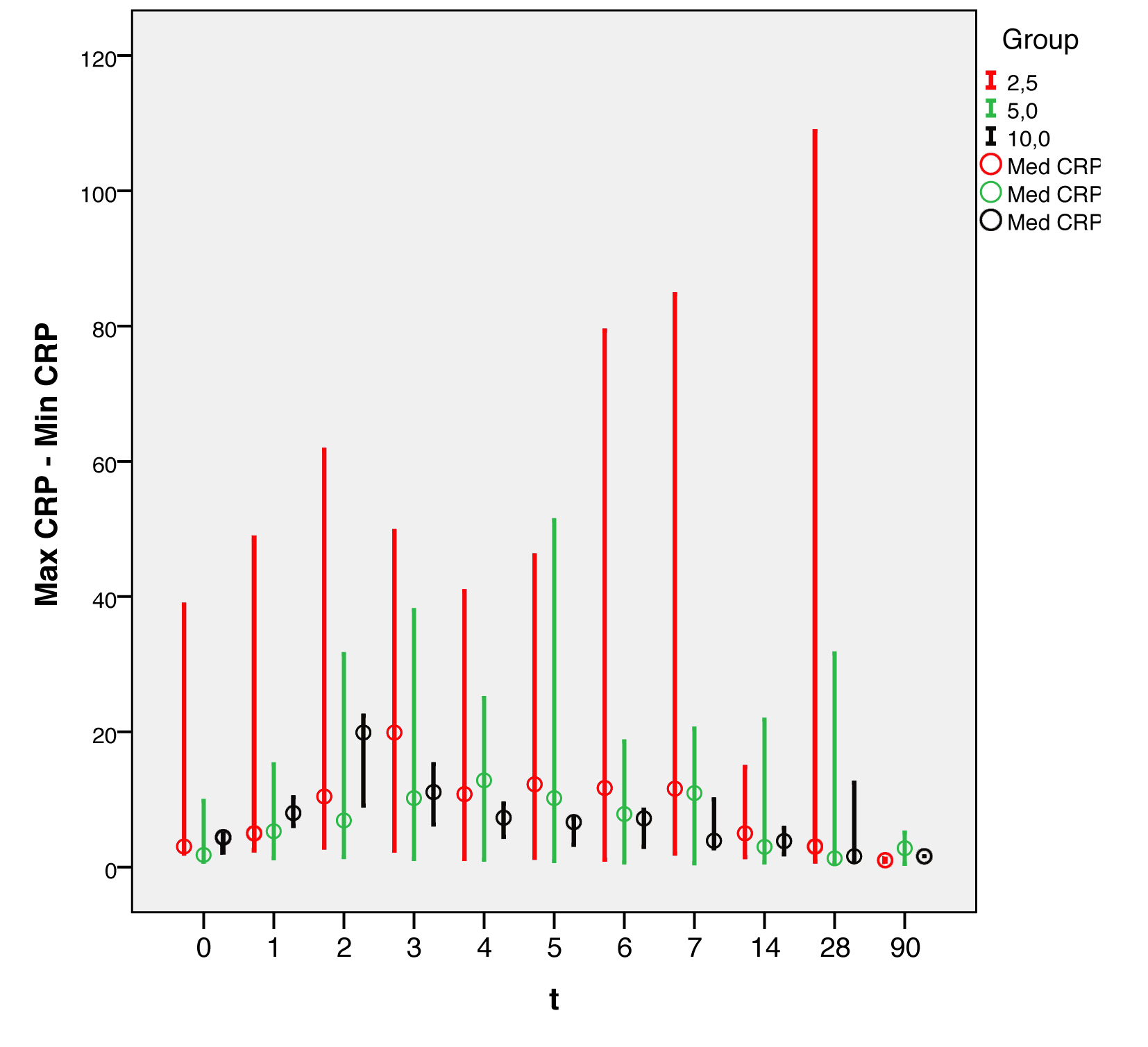

Supplement: Figure S1 — CRP (c-reactive protein) in mg/dl over time as median with minimum and maximum in the three different dosage groups. (TIF) [file pone.0023099.s001.tif]

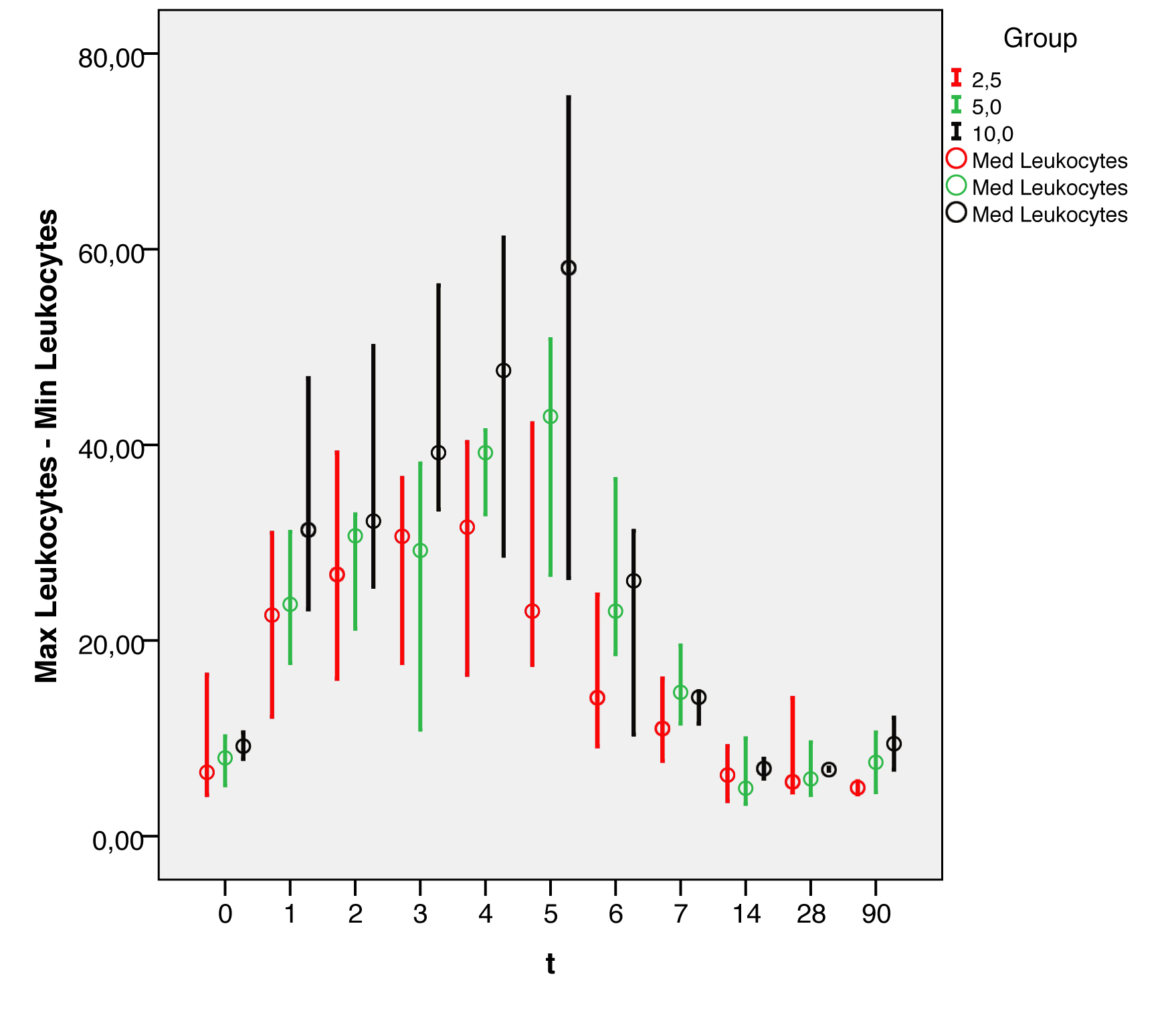

Supplement: Figure S2 — Leukocytes/µl over time as median with minimum and maximum in the three different dosage groups. (TIF) [file pone.0023099.s002.tif]
