# Supplementary material for: Mobilisation of Hematopoietic CD34+ Precursor Cells in Patients with Acute Stroke Is Safe - Results of an Open-Labeled Non Randomized Phase I/II Trial
Source: PLoS One. 2011 Aug 26;6(8):e23099. doi: 10.1371/journal.pone.0023099 (PMC3162562; doi:10.1371/journal.pone.0023099)
Supplement: Table S1 — Patients included in the VGM analysis. The table shows the 8 individuals' gender and age and the timing of sequential MRIs after stroke onset (first to third MRI in days, fourth MRI in months). (DOC) [file pone.0023099.s003.doc]

**Table S1**

| Patient | Age | Gender | 1st MRI (days) | 2nd MRI (days) | 3rd MRI (days) | 4th MRI (months) |
| --- | --- | --- | --- | --- | --- | --- |
| No. |
| 5 | 55 | F | 2 | 7 | - | - |
| 8 | 65 | M | 1 | 6 | 106 | - |
| 10 | 54 | M | 1 | 7 | 91 | - |
| 12 | 46 | F | 1 | 5 | 89 | 62 |
| 13 | 55 | F | 2 | 7 | 90 | 58 |
| 14 | 52 | M | 2 | 7 | - | - |
| 15 | 53 | M | 3 | 7 | - | - |
| 19 | 64 | M | 2 | 7 | 92 |  |
