# Supplementary material for: Mobilisation of Hematopoietic CD34+ Precursor Cells in Patients with Acute Stroke Is Safe - Results of an Open-Labeled Non Randomized Phase I/II Trial
Source: PLoS One. 2011 Aug 26;6(8):e23099. doi: 10.1371/journal.pone.0023099 (PMC3162562; doi:10.1371/journal.pone.0023099)
Supplement: Protocol S1 — Regeneration in Acute Ischemic Stroke (RAIS) [German: Regeneration nach akutem ischämischen Schlaganfall — Einarmige, Dosis-eskalierte klin. Phase I/II - Studie zur hämato-poetischen Stammzell-Mobilisation bei akutem ischämischen A. cerebri media –Teilinfarkt]. (PDF) [file pone.0023099.s005.pdf]

# Regeneration nach akutem ischämischen Schlaganfall

## - Regeneration in Acute Ischemic Stroke (RAIS) –

**(Einarmige, Dosis-eskalierte klin. Phase I/II - Studie zur hämatopoetischen Stammzell-Mobilisation bei akutem ischämischen A. cerebri media -Teilinfarkt)**

**Leiter der klinischen Prüfung:**

Prof. Dr. med. Ulrich Bogdahn
Direktor der Klinik und Poliklinik für Neurologie
im Bezirksklinikum Regensburg
Universität Regensburg
Universitätsstrasse 84
93053 Regensburg
Tel.: 0941 / 9 41 3001
Fax: 0941 / 9 41 3005
e-mail: ulrich.bogdahn@klinik.uni-regensburg.de

**Studienkoordination:**

OA Prof. Dr. med. Jürgen Winkler
OA Dr. med. Markus Horn
OA Dr. med. Guntram Ickenstein

**Beteiligte Mitarbeiter:**

Stroke Unit:

Dr. Markus Horn
Tel.: 0941 / 9 41 3330
e-mail: markus.horn@klinik.uni-regensburg.de

Dr. Guntram Ickenstein
Tel.: 0941 / 9 41 3012
e-mail: guntram.ickenstein@klinik.uni-regensburg.de

Dr. Petr Erban
Tel.: 0941 / 9 41 3202
e-mail: petr.erban@bkr-regensburg.de

Dr. Monika Seidl
Tel.: 0941 / 9 41 3202
e-mail: monika.seidl@bkr-regensburg.de

Dr. Nils Bijan Vatankhah
Tel.: 0941 / 9 41 3202
e-mail: bijan.vatankhah@bkr-regensburg.de

Dr. Sophie Sauerbruch
Tel.: 0941 / 941 3202
e-mail: sophie.sauerbruch@bkr-regensburg.de

## Intensivmedizin:

Dr. Wolfgang Jakob, Anästhesist
Tel.: 0941 / 9 41 3331
e-mail: wolfgang.jakob@bkr-regensburg.de

Dr. Bernhard Kaiser, Kardiologe
Tel.: 0941 / 9 41 3041
e-mail: bernhard.kaiser@bkr-regensburg.de

## Bildanalyse

PD Dr. Schuierer Gerhard
Dr. Victoria Villarrubia
Institut für Neuroradiologie
im Bezirksklinikum Regensburg
Universitätsstrasse 84
D 93053 Regensburg
Tel.: 0941 / 9 41 1300
Fax: 0941 / 9 41 1305
e-mail: gerhard.schuierer@bkr-regensburg.de

PD Dr. Arne May
Tel.: 0941 / 9 41 3320
e-mail: arne.may@klinik.uni-regensburg.de

## Neuropsychologie

Dr. Ralf Lürding
Tel.: 0941 / 9 41 3043
e-mail: ralf.lürding@bkr-regensburg.de

## Stammzellmonitoring

Prof. Dr. Reinhard Andreesen

PD. Dr. Burkhard Hennemann
Abt. Hämatologische Onkologie
Klinikum der Universität Regensburg
Franz-Josef-Strauss-Allee 11
93053 Regensburg
Tel.: 0941 / 9 44 5531
Fax: 0941 / 9 44 5511
e-mail: burkhard.hennemann@klinik.uni-regensburg.de

## Klinisch-Chemisches Labor

Prof. Dr. Gerd Schmitz
PD.Dr. Gregor Rothe
Institut für Klin. Chemie und Transfusionsmedizin
Universitätsklinik Regensburg,
Franz-Josef Strauss Allee 58
D-93053 Regensburg
Tel. 0941-944-6200
Fax: 0941-944-6202
e-mail: gerd.schmitz@klinik.uni-regensburg.de

## Beteiligte Institute:

### Klinik und Poliklinik für Neurologie im BKR:

Prof. Dr. med. Ulrich Bogdahn
Direktor der Klinik und Poliklinik für Neurologie
im Bezirksklinikum Regensburg
Universität Regensburg
Universitätsstrasse 84
93053 Regensburg
Tel.: 0941 / 9 41 3001
Fax: 0941 / 9 41 3005
e-mail: ulrich.bogdahn@klinik.uni-regensburg.de

### Institut für Neuroradiologie im BKR:

PD Dr. Schuierer Gerhard

Dr. Victoria Villarrubia
Institut für Neuroradiologie
im Bezirksklinikum Regensburg
Universitätsstrasse 84
D 93053 Regensburg
Tel.: 0941 / 9 41 1300
Fax: 0941 / 9 41 1305
e-mail: gerhard.schuierer@bkr-regensburg.de

### Abtlg. Hämatologie und Onkologie:

Prof. Dr. Reinhard Andreesen

PD. Dr. Burkhard Hennemann
Abt. Hämatologische Onkologie
Klinikum der Universität Regensburg
Franz-Josef-Strauss-Allee 11
93053 Regensburg
Tel.: 0941 / 9 44 5531
Fax: 0941 / 9 44 5511
e-mail: burkhard.hennemann@klinik.uni-regensburg.de

### Institut für Klinische Chemie

Prof. Dr. Gerd Schmitz
PD.Dr. Gregor Rothe
Institut für Klin. Chemie und Transfusionsmedizin
Universitätsklinik Regensburg,
Franz-Josef Strauss Allee 58
D-93053 Regensburg
Tel. 0941-944-6200
Fax: 0941-944-6202
e-mail: gerd.schmitz@klinik.uni-regensburg.de

## Externes Protokoll-Review Kommitee:

**Neurologie:**
**Prof. Dr. Roman Haberl**
Direktor der Neurolog. Klinik
Krankenhaus München Harlaching
Sanatoriumsplatz 2
81545 München
Tel. 089-6210-2257/58
Fax. 089-6210-2453
e-mail: rhaberl@extern.irz-muenchen.de

**Neuroradiologie:**
**Prof. Dr. Michael Schumacher**
Neurozentrum - Direktor der Sektion Neuroradiologie
Dekan der Med. Fakultät
Klinikum der Universität Freiburg
Braisacher Str. 64
79106 Freiburg
Tel. 0761-2705180
Fax. 0761-2705195
e-mail. schu@nz.ukl.uni-freiburg.de

**Hämatoonkologie:**
**Prof. Dr. Christian Peschel**
Direktor der 3. Medizinischen Klinik
Technische Universität München
Ismaninger Str. 22
89675 München
Tel. 089-41404110
Fax. 089-41404879
e-mail: christian.peschel@lrz.tu-muenchen.de

Wichtiger Hinweis: Dieser Prüfplan enthält vertrauliche Daten, die ausschliesslich den Mitgliedern des Protkoll-Komitees, der Ethikkommission sowie im Bedarfsfall Vertretern befugter Behörden zugänglich gemacht werden dürfen. Der Prüfplan dient der Durchführung und Auswertung der klinischen Studie und darf nicht ohne schriftliche Genehmigung des Protokoll-Komitees weitergegeben werden.

## Inhaltsverzeichnis

**1. Einleitung**

**1.1** Studienhintergrund

**1.2** Studienrationale

**1.3** Risikoabschätzung in präklinischen Studien - Grundlagen zu G-CSF

**1.4** Risikoabschätzung in klinischen Studien mit G-CSF

**1.5** Pharmakokinetische und pharmakodynamische Eigenschaften von G-CSF (Neupogen®)

**1.6** Funktionelles Outcome und Lebensqualität

**2. Studienziele**

2.1 Primäres Studienziel

2.2 Sekundäres Studienziel

**3. Studiendesign**

3.1 Art der Studie

3.2 Patientenzahl

3.3 Zeitplan

**4. Patientenauswahl**

4.1 Einschlusskriterien

4.2 Ausschlusskriterien

**5. Prüfmedikation**

## 6. Begleit- und Supportivmedikation

6.1 Basistherapie des Schlaganfalles

6.1.1 Blutdruck-Management
6.1.2 Gewährleistung adäquater Oxygenierung
6.1.3 Flüssigkeits-und Elektrolythaushalt
6.1.4 Glukosestoffwechsel
6.1.5 Körpertemperatur
6.1.6 Allgemeine Prophylaxe

6.2 Indikationen zur PTT-wirksamen i.v. Heparinisierung in der Akutphase des Schlaganfalles

6.3 Sekundärprävention nach Schlaganfall

## 7. Behandlungsplan und Dosierungsvorschriften

7.1 Therapiemodifikationen

## 8. Untersuchungen

8.1 Zeitpunkte der Untersuchungen

8.2 Umfang der Untersuchungen

8.3 Tabellarische Auflistung des Untersuchungsumfanges

## 9. Therapiebewertung

9.1. Primäre Endpunkte

9.2. Sekundäre Endpunkte

## 10. Sicherheit (Toxizität)

10.1 Erfassung und Dokumentation von AEs, SAE´s und ADR`s

10.2 Unerwünschte Ereignisse – Adverse Events (AE)
10.2.1 Meldung von AE´s

10.3. Schwerwiegende unerwünschte Ereignisse – Serious Adverse Events (SAE)
10.3.1 Meldung von SAE´s

10.4 Unerwünschte Arzneimittelwirkung – Adverse Drug Reaction (ADR)
10.4.1 Meldung von ADR´s

## 11. Vorzeitiges Studienende

11.1 Für den Patienten
11.2 Für die gesamte Studie

## 12. Dokumentation

## 13. Datenmanagment und statistische Aspekte

13.1 Datenerhebung

13.2 Statistische Methoden

13.3 Auswertbarkeit und Auswertung

## 14. Monitoring und Qualtätskontrolle

## 15. Ethische und regulatorische Belange

15.1 Ethikkommission
15.2 Patienteninformation und Datenschutz
15.3 Patientenversicherung
15.4 Studienfinanzierung
15.5 Prüfplanergänzungen
15.6 Gesetzliche Bestimmungen  und Richtlinien
15.7 Allgemeine Anzeigepflicht
15.8 Prüfarztinformationen

## 16. Vereinbarungen und organisatorische Abläufe

16.1 Leiter der klinischen Prüfung und Studienzentrale

16.2 Studienkoordination, medizinische Dokumentation und Datenmanagement

## 17. Literaturverzeichnis

## 18. Anhänge

| | | |
|---|---|---|
| 18.1. | Appendix A: | Patientenaufklärung |
| 18.2. | Appendix B: | Einverständniserklärung |
| 18.3. | Appendix C: | Patientenversicherung |
| 18.4. | Appendix D: | Ethikvotum |
| 18.5. | Appendix E: | Deklaration von Helsinki |
| 18.6. | Appendix F: | Produktinformationen |
| 18.7. | Appendix G: | Datenerfassungsbögen (CRF) |
| 18.8. | Appendix H: | Liste zur Identifikation der Patienten |
| 18.9. | Appendix I: | NIH-Stroke Scale (NIHSS) |
| 18.10. | Appendix J: | Modified Rankin Scale (RS) |
| 18.11. | Appendix K: | Barthel Index (BI) |
| 18.12. | Appendix L: | Functional Independence Measures (FIM) |
| 18.13. | Appendix M: | Testpsychologie |
| 18.14. | Appendix N: | SSQL |
| 18.15. | Appendix O: | Bericht über schwerwiegende unerwünschte Ereignisse (SAE´s) |
| 18.16. | Appendix P: | Bericht über unerwünschte Arzneimittelwirkungen (BfArM) |
| 18.17. | Appendix Q: | Synopsis Study Protokoll |

# 1. Einleitung

## 1.1 Studienhintergrund

Neuronale Stamm- und Vorläuferzellen finden sich im adulten menschlichen Gehirn (Gage FH et al., Science 2000). Es gibt eine ganze Reihe von Hinweisen, dass eine ihrer wesentlichen Aufgaben darin bestehen könnte, eine Rolle in der Reparatur von Hirnparenchymschädigungen und somit in der funktionellen Plastizität als Antwort auf Hirnschädigung bzw. kognitive Stimulation zu spielen (Brazelton TR et al., Science 2000, Nakatomi et al. Cell 2002). Gegenwärtig gibt es experimentelle Hinweise dafür, dass hämatopoetische Stammzellen in organspezifische – in diesem Fall phänotypisch neuronale–pluripotente Vorläuferzellen differenzieren können (Mezey E et al. Science 2000, Zivin et al. Neurology 2000).

Es zeigen sich eine ganze Reihe von unmittelbaren Beziehungen und Verwandschaften zwischen der hämatopoetischen und der neuronale Stammzellentwicklungslinien, wie sie sich in gemeinsamen Zelloberflächenantigenen, Zytokinen, Wachstumsfaktoren, Matrixkomponenten und Signaltransduktionswegen widerspiegeln (Scheffler et al. Trends Neurosc. 1999). Konsequenterweise führt die intravenöse, intraarterielle und intrazerebrale Applikation hämatopoetischer Stammzellen zum Überleben dieser Zellen im ZNS, zur Migration und Differenzierung innerhalb des Zentral-Nervensystems (ZNS), spezifischerweise begleitet von einer signifikanten funktionellen Verbesserung in experimentellen Modellsystemen für zerebrale Ischämie, die dem klinischen Schlaganfallsbild weitestgehend ähnlich sind (Li et al., Neurology 2001, Gu et al. JCerBlMet 2000). Experimente mit zusätzlicher Gabe von neurotrophen Wachstumsfaktoren während der Experimente verstärken diese Beobachtungen (Chen et al., Stroke 2001).

Ähnliche Ergebnisse wurden von experimentellen Modellsystemen des Myokardinfarktes berichtet: Die Transplantation von hämatopoetische Stammzellen über eine intravenöse bzw. intraarterielle Applikation führt in einem Myokardinfarktmodell zu einer signifikanten kardialen morphologischen und funktionellen Verbesserung, wobei Differenzierungen in endotheliale und kardiomyozytäre Zellpopulationen beobachtet werden (Orlic et al., Nature 2001): in dieser Serie konnte die Gesamtmortalität um 68 %, die Infarktgröße um 40 %, der diastolische Stress um 70% gesenkt werden. Ejektionsfraktion und hämodynamische Parameter verbesserten sich signifikant als Konsequenz von neuer Kapillarisierung und Kardiomyozyten-Einsprossung. Bisher gibt es nur ganz vorläufige Daten zur Mobilisierung von Stammzellen in diesen Modellsystemen.

Die zerebrale Ischämie scheint nach älteren Literaturangaben nicht ausreichend wirksam zu sein, um eine G-CSF-mRNA-Expression in Tiermodellen zu induzieren (Hierholzer et al., Transplant Proceedings 1998). In einer größeren humanen Untersuchungsserie von Ohara et al. sowie in eigenen präliminären Voruntersuchungen der Arbeitsgruppe von Prof. Andreesen / Prof. Bogdahn (Hennemann, Horn, Winkler) konnte dagegen ein signifikanter Anstieg der Serumspiegel von G-CSF in Schlaganfallpatienten beobachtet werden (Ohara et al., Life Science 1993), insbesondere im Vergleich zu anderen zerebralen Erkrankungen.

## 1.2 Studienrationale

Die Therapie des akuten Schlaganfalles besteht nach dem derzeitigen Kenntnisstand im wesentlichen aus Strategien, die sich ursächlich auf die Öffnung des verschlossenen Gefäßes fokussieren. So steht die Rekanalisation mittels Thrombolyse respektive die Behandlung der Gefäßkomplikationen wie Dissektion oder Pseudookklusion durch einen entsprechenden operativen Ansatz oder die konsequente Antikoagulation im Vordergrund (Albers G et al., Chest 1998). Der tatsächliche parenchymatöse Schaden wird derzeit durch keine Therapie ursächlich behandelt.

Im Anschluss an die Akutintervention stehen im wesentlichen rehabilitative Massnahmen im Vordergrund. Neuroprotektive Strategien haben sich sowohl experimentell als auch klinisch bisher nicht durchgesetzt. Der Kenntnisstand bezüglich des endogenen zellulären Potentials sowohl im adulten ZNS, als auch in anderen Organsystemen hat rapide zugenommen. Nach dem derzeitigen Kenntnisstand existieren sowohl neuronale Stamm- und Vorläuferzellen in unterschiedlichen Hirnregionen, die z. T. kontinuierlich sich selbst erneuern, respektive als ruhende Zellen beobachtbar sind. Diese Zellen haben die Eigenschaft, Läsionen im tierexperimentellen Bereich zu reparieren (Magavi et al. Nature 2000, Mezey et al., Science 2000; Li et al., Neurology 2001).

Es liegen ebenfalls zunehmend experimentelle Daten vor, dass hämatopoetische Stammzellen ebenfalls in das ZNS migrieren und dort einen neuronalen Phänotyp annehmen können (Zhao et al. Exper Neurology 2002, Chen et al., Stroke 2001). Man geht derzeit davon aus, dass das Transdifferenzierungspotential zirkulierender hämatopoetischer Stammzellen geeignet ist, nicht nur das Hirnparenchym zu besiedeln, sondern sich ebenfalls funktionell neuronal als auch glial zu integrieren (Iwai et al. J Cerebr Blood Flow Met 2001). Zusätzlich konnte gezeigt werden, dass durch die systemische Applikation von hämatopoetischen Stammzellen Reparaturvorgänge induzierbar sind (Kee et al. Exp Brain Res 2001).

Zerebro-vaskuläre Erkrankungen sind derzeit durch eine enorme Morbidität und Invalidität gekennzeichnet und stellen eine außerordentliche sozio-ökonomische Belastung für die westlichen Industrienationen dar. Das vorliegende Forschungsvorhaben basiert darauf, die effektiven Strategien der Behandlung der Gefäßkomplikationen beizubehalten, jedoch zusätzlich das endogen vorhandene Potential hämatopoetischer Stammzellen (eigene Vorarbeiten) zu rekrutieren und dafür zu benutzen, den entstehenden parenchymatösen Schaden besser behandeln zu können. Das Konzept sieht somit vor, neben der thrombolytischen Therapie einen zelltherapeutischen Ansatz zu verfolgen. Wir gehen davon aus, dass die zusätzliche Mobilisierung von hämatopoetischen Stammzellen aus dem Patienten eigenen humanen Knochenmark mittels einer systemischen G-CSF-Gabe einen wichtigen zusätzlichen regenerativen Schritt darstellen könnte. Diese Ergebnisse könnten eine auf dem Einsatz von adulten endogenen Stammzellen basierende Zelltherapie für Gefäßerkrankungen des zentralen Nervensystems etablieren.

## 1.3 Risikoabschätzung in präklinischen Studien - Grundlagen zu G-CSF

Aus zahlreichen Studien der letzten Jahre ließ sich dokumentieren, dass G-CSF in der Lage ist, den Anteil von $CD^{34}$-positiven Vorläuferzellen (peripheren Stammzellen) sowohl in gesunden Spendern als auch Patienten deutlich zu erhöhen (Tamura et al. Biochem Biophys res Commun 1987). Da $CD^{34}$-Zellen auch im Zusammenhang gebracht werden mit der Repopulation von Organen nach spezifischer Organschädigung und möglicher-

weise Vorstufen der neuronalen Precursorzellen sind, liegt es nahe, G-CSF in einem Mobilisierungsexperiment bei akuten ZNS-Schädigungen einzusetzen. Hiermit soll die Repopulation des ZNS mit $CD^{34}$-positiven Zellen/neuronalen Precursorzellen gefördert werden. Erste Publikationen aus dem tierexperimentellen Bereich deuten darauf hin, dass dies gelingen könnte (Scheffler et al. Trends Neurosci 1999): Neuere tierexperimentelle Studien in einem Okklusionsmodell an der Ratte konnten sogar einen neuroprotektiven Effekt von G-CSF in Rahmen eines voruebergehenden Verschlusses der Art. cerebri media (ACM) nachweisen: das Infarktvolumen konnte durch die Gabe von 60 µg/kg KG über 90 min i.v. um ca. 50% reduziert werden, eine Toxizität durch Leukozyten wurde nicht beobachtet. Die Expression von G-CSF Rezeptoren im Gehirn konnte mittels Westernblot und PCR nachgewiesen werden. Zudem konnte in einer Zellkultur gezeigt werden, dass vorwiegend Neurone von dem protektiven Effekt von G-CSF profitieren (Schaeblitz et al. 2001).

Während G-CSF die Bildung von Neutrophilen aus der hämatopoetischen Reihe deutlich stimuliert, scheint es eher einen Negativeffekt auf die Erythropoese zu haben; dieser ist jedoch im wesentlichen bei prolongierter Applikation zu beobachten. Während GM-CSF zu einer doch erheblichen Thrombozytose führen kann, scheint G-CSF auf die Thrombopoese nur einen geringeren Einfluss zu haben (McQualter et al. J Exp Med 2001; Morstyn et al., 1997).

Auch Monozyten werden durch G-CSF stimuliert und in ihrer Anzahl im peripheren Blut vermehrt. Die Effekte von G-CSF auf die hämatopoetische Zellreihe sind am ehesten durch eine Stimulation des Eintrittes der ruhenden hämatopoetischen Stammzellen in die Zellzyklus-S-Phase erklärbar. Damit wird auch verständlich, dass es zu einem deutlichen, allerdings zeitlich etwas retardierten Anstieg der $CD^{34}$-positiven Zellen kommt. Dies ist auch die Grundlage für die Mobilisierung von peripheren hämatopoetischen Vorläuferzellen durch G-CSF und GM-CSF (Hierholzer et al. Transplant Proc. 1998; Magavi S et al. Nature 2000).

## 1.4 Risikoabschätzung in klinischen Studien

Warum ist es notwendig, zum jetzigen Zeitpunkt eine klinische Studie zu initiieren, in der hämatopoetische Stammzellen mobilisiert werden sollen, um ein besseres Schlaganfall-Outcome zu erreichen?

Das wissenschaftliche Schlaganfallsfeld hat eine Zeit multipler klinischer Ansätze für eine Gefäß- und parenchymatöse Neuroprotektion mit dem Ziel einer Neuroregeneration hinter sich, die leider allesamt enttäuschend verliefen. Daher ist es aus wissenschaftlichen, aber auch aus ökonomischen Gründen ausgesprochen vielversprechend und dringlich notwendig, über Stammzelltechnologien, die ethisch unbedenklich sind, einen klinischen Ansatz zur regenerativen Therapie von ischämisch-induzierten Hirnparenchymschädigungen zu entwickeln und erfolgreich zu etablieren.

Das Wissen bezüglich der allgemeinen Stammzellbiologie hat sich in den letzten Jahren vervielfacht. Die meisten neurobiologischen Daten bezüglich einer potentiellen Rolle von Stammzellen für die Neuroregeneration stammen allerdings aus Daten von Nagern, bei denen ein wesentlich höheres endogenes regeneratives Potential besteht und eine funktionelle Erholung im ZNS und im Rückenmark häufig spontan beobachtet werden kann (Tamura et al. 1987; Nakatomi et al. Cell 2002).

Allerdings ist die Wahrscheinlichkeit für eine erfolgreiche Anwendung von mobilisierten

Stammzellen im humanen System nach einer akuten hypoxischen Gewebsschädigung, nach allem, was wir bisher wissen, ausgesprochen hoch. Strukturelle, funktionelle und metabolische Bildgebung sowie neuropsychologische und neurophysiologische klinische Untersuchungstechniken sind sehr weit fortgeschritten, so daß klinische Daten im humanen System uns sehr rasch neue Einsichten in die potentielle Rolle von humanen, adulten mobilisierten Stammzellen und ihre Möglichkeiten, Regeneration zu induzieren, geben werden.

Im vorgelegten Studienprotokoll zur stammzellbasierten Therapie des Schlaganfalles soll das bereits zugelassene Präparat Neupogen® (Filgrastim, r-Hu-G-CSF) zur Mobilisierung von peripheren Stammzellen bei Schlaganfallpatienten eingesetzt werden. G-CSF ist eines aus einer ganzen Reihe gut charakterisierter knochenmarksaktiver Zytokine, über das im klinischen Bereich insbesondere zwei große Erfahrungspotentiale bestehen

1. wird G-CSF in der ablativen Hochdosis-Chemotherapie solider Tumoren und hämatopoetischer maligner Erkrankungen eingesetzt.

2. wird G-CSF für die Knochenmarksspende gesunder Probanden zur Mobilisierung von peripheren Stammzellen mit nachfolgender Leukapherese eingesetzt. Hier bestehen auch die größten Erfahrungen in der Anwendung von G-CSF im gesunden Organismus.

Die Rolle von G-CSF in der normalen Hämatopoese ist gut untersucht. Normale Plasmaspiegel im Gesunden bewegen sich bei etwa 10 pg/ml, bei neutropenischen Patienten werden Spiegel bis zu 100.000 pg/ml beobachtet (Morstyn et al., 1997). G-CSF ist, wie viele andere Untersuchungen belegen, daher physiologischerweise in der Knochenmarksantwort auf unterschiedliche Ursachen, die zu Neutropenie führen, involviert. Hierbei dient es offensichtlich der Repopulation des peripheren Blutes mit Leukozyten (Tamura et al. Biochem Biophys Res Commun 1987). Aus klinischer Sicht ist es momentan daher das wichtigste Zytokin, um die Regeneration des Knochenmarks zu fördern. Hohe Konzentrationen von G-CSF in der Amnionflüssigkeit sowie im mütterlichen Plasma während des letzten Trimesters der Schwangerschaft deuten auf eine ebenso wichtige Bedeutung in der Entwicklung des kindlichen Knochenmarks hin, ähnlich wie hohe Konzentrationen im Nabelschnurblut. Aus tierexperimentellen Überexpressionsstudien mit G-CSF lässt sich seine Rolle für die Hämatopoese durchaus von der anderen Seite beleuchten, hierbei kommt es zu einer signifikanten Neutrophilie sowie der Infiltration verschiedener Organe durch Neutrophile bzw. Knochenmarksvorläuferzellen. Diese ist aus unserer Sicht für eine Anwendung bezüglich Organrepair am ZNS besonders wichtig.

G-CSF wird in dieser Studie zur Mobilisierung von Stammzellen bei Schlaganfallpatienten eingesetzt. Hierüber gibt es bisher keine klinischen Daten in der Literatur. Allerdings kann man wohl davon ausgehen, dass ein ähnliches Toxizitätsprofil besteht, wie es bei der Mobilisierung von Stammzellen in gesunden Spendern zu erwarten ist. Die am häufigsten beobachteten unerwünschten dosisabhängigen Nebenwirkungen sind hier leichte bis mäßige, vorübergehende Schmerzen der Skelettmuskulatur bzw. Knochenschmerzen und treten bei ca. 20% der Patienten auf (Morstyn et al., 1997). Diese Nebenwirkungen können sehr einfach durch Gabe von Paracetamol® kontrolliert oder verhindert werden. Bei 41% der Spender wurde eine Leukozytose beobachtet (Majolino et al. 1997). Da eine Leukapherese nicht vorgesehen ist, ist möglicherweise von der sonst beobachteten Thrombozytopenie in Kombination mit G-CSF-Gabe und Leukapherese nicht aus-

zugehen. Weist die Anamnese eine Überempfindlichkeit des Patienten für E. coli-Produkte aus, sollte auf eine Anwendung von G-CSF verzichtet werden.

G-CSF wurde in klinischen Studien an gesunden Spendern und Patienten generell gut toleriert. In einer Follow-up-Studie zeigten Patienten über einen Zeitraum von drei Jahren keinerlei Verträglichkeitsprobleme unter einer Therapie mit rhG-CSF (mittlere rhG-CSF Dosis 16 µg/kgKG über einen Median von 6 Tagen - Cavallaro et al. 2000). Auch wenn rhG-CSF beim Menschen eventuell die Koagulationskaskade beeinflusst, so zeigten sich jedoch keinerlei schädliche Auswirkungen, was anhand von zerebralen Mikroembolisationsmessungen mittels transkranieller Doppleruntersuchung bestimmt wurde (Sohngen et al., Bone Marrow Transplant 1998). Zudem gibt es bereits zahlreiche Verträglichkeitsstudien von rhG-CSF in Patienten mit hämatopoetischen und onkologischen Grunderkrankungen. Diese Untersuchungen ergeben keinen Hinweis darauf, dass nach rhG-CSF Applikation das Risiko für einen Schlaganfall ansteigt (Morstyn et al., 1997).

Aus den zahlreichen Erfahrungen hinsichtlich der Mobilisation von Stammzellen bei gesunden und bei onkologischen Patienten lässt sich subsumieren, dass nicht nur das periphere Blut eine wesentlich effektivere Quelle für transplantierbare Vorläuferzellen darstellt, sondern auch das Knochenmark von einer G-CSF-Therapie stark profitiert: es findet sich eine Zunahme von $CD^{34+}$ Zellen unter Zytokin-Behandlung. Auch bis zu 14 Tagen nach einer solchen Behandlung findet sich in einem behandelten Knochenmark eine etwa 10-fach höhere reproduktive Aktivität als in unbehandelten Kontrollen. (Morstyn et al., 1997; Waller et al., 1996).

Durch die Behandlung der Patienten mit rhG-CSF kommt es zu einem Anstieg der Leukozytenzahl im Blut. Bisher konnte in keiner Studie ein direkter kausaler Zusammenhang hergestellt werden zwischen Leukozytose und erhöhtem Schlaganfallrisiko, abgesehen von Leukozytose>100,000/µl, was mit rheologischen Komplikationen verbunden ist (Schmid-Schönbein et al. Microvasc Res 1980; Chien et al. 1995). Bei den von uns gegebenen Dosierungen im Rahmen einer Eskalationsstudie sind Leukozytenwerte<70,000/µl zu erwarten, die zu keinerlei Komplikationen führen sollten. Leukozytose im Rahmen eines Schlaganfallgeschehens könnte eventuell den frustranen Versuch des Organismus darstellen, Stammzellen aus dem Knochenmark zu mobilisieren, um endogene Reparaturmechanismen einzuleiten. Zudem initiieren Leukozyten und Granulozyten vermutlich eine Abräumreaktion im Infraktareal mit nachfolgender Glia- bzw. Narbenbildung (Slevin et al. Stroke 2000).

Die Effekte von G-CSF sind durchwegs dosisabhängig, sowohl in präklinischen als auch in klinischen Experimenten (Morstyn et al. 1997). Bezüglich der klinischen Anwendung zur Mobilisation von Stammzellen hat sich eine fünfmalige intravenöse Gabe an 5 aufeinanderfolgenden Tagen von 10 µg/kgKG als geeignet erwiesen, einen ausreichenden Effekt zu produzieren und am besten verträglich zu sein. Es kommt nach etwa 2–3 Tagen zu einem leichten Anstieg der Gesamtleukozyten auf ca. 20.000 /µl. Dosen von 30–60 µg/kgKG führen zu höheren Leukozytenzahlen, so dass man für die Knochenmarksspende (Stammzellenmobilisierung) daher allgemein 10 µg/kgKG benutzt (Morstyn et al., 1997). Um diesem Befund Rechnung zu tragen und um eine zusätzliche Sicherheit für den Patienten in dieser Studie zu gewährleisten, soll eine Dosisreduktion um 50% durchgeführt werden, sobald die Leukozytenzahl>20.000/µl überschreitet. Das übrige Studienprotokoll wird unverändert fortgeführt.

Die Arbeitsgruppe um Slevin et al. konnte zeigen, dass VEGF (vascular endothelial growth factor) in höheren Konzentrationen im Serum von Schlaganfallpatienten nachzu-

weisen ist. VEGF ist als wichtiges Zytokin vor allem in der Penumbra des Infarktareals erhöht und wird mit Angiogenese, Gewebsinflammation und Fibrosierung in Verbindung gebracht. Da die VEGF-Konzentrationen positiv mit der Schwere des Schlaganfalles korrelieren, vermuten die Autoren einen Zusammenhang mit dem Reparaturmechanismus des Körpers und dem Versuch, einer teilweisen Wiederherstellung des Gehirnareals (Slevin et al. Stroke 2000).

Ein wesentlicher Teil des positiven Effektes der Leukozytose und der Stammzellmobilisierung auf die Verbesserung des funktionellen Outcome nach Schlaganfall könnte dementsprechend nicht nur durch die Funktionalität der neuen Zellen selber, sondern auch durch deren Auswirkung auf das Gehirn, bespielsweise durch Ausschüttung trophischer Faktoren wie Interleukine, Stem-Cell-Factor oder Colonie-stimulierende Faktoren (CSF-1) bedingt sein.

## 1.5 Pharmakokinetische und pharmakodynamische Eigenschaften von G-CSF (Neupogen®)

Die beiden Haupteffekte von G-CSF, die sich als pharmakologische Endpunkte eignen, sind einmal die Mobilisierung von neutrophilen Granulozyten aus dem bestehenden Knochenmarkspool und hiermit möglicherweise indirekt auch eine Mobilisierung von $CD^{34+}$ positiven Zellen innerhalb weniger Stunden nach Applikation der Substanz. Zum Zweiten findet sich die bereits oben erwähnte Stimulation der Hämatopoese, insbesondere der Granulopoese: durchschnittlich erscheinen experimentell markierte Granulozyten aus dem G-CSF-stimulierten Knochenmark etwa 3 Tage nach einer einmaligen s. c. Dosis von 300 µg im peripheren Blut. Eine überschießende Reaktion wird teilweise durch die Zunahme von G-CSF-Rezeptor-tragenden Zellen, die freies G-CSF über Rezeptor-Bindung wegfangen, vermieden.

Nach subkutaner Gabe wird Neupogen® rasch absorbiert, die Anstiege der Plasmakonzentrationen finden sich bereits 2–8 Stunden nach der Gabe. Die Eliminationshalbwertszeit nach i. v. und s. c. Gabe liegt bei ca. 2–4 Stunden, wobei Clearance und Halbwertszeit sowohl von der Dosis als auch von der Neutrophilen-Anzahl (entsprechende G-CSF-Rezeptordichte) abhängig sind.

Es bestehen ausführliche Daten zur klinischen Pharmakokinetik (i. v. und s. c.) in gesunden Probanden: nach i. v. Gabe findet sich eine Halbwertszeit von etwa 2,7 Stunden mit einem Verteilungsvolumen von 162 ml/kgKG. Nach subkutaner Gabe finden sich dosisabhängige Halbwertszeiten von 1,6–16,28 Stunden mit Expositionsdosen von 119 bis max. 209 ng x Stunde/ml.

Eine ganze Reihe von pharmakokinetischen Daten sind für Tumorpatienten verfügbar, die sich z. T. erheblich voneinander unterscheiden. Aus pharmakodynamischer Sicht lässt sich zeigen, dass nach kontinuierlicher Infusion mit 10 µg/kg pro Tag die Neutrophilen am Tag 2 bereits kräftig ansteigen und ihr Maximum am Tag 4–5 erreichen. Die G-CSF-Spiegel erreichen hier unter diesen Bedingungen am Tag 3 ihr Maximum mit 20 ng/ml und fallen dann rasch ab durch die Clearance über G-CSF-Rezeptoren. Qualitativ findet sich nach G-CSF-Gabe ein deutlicher Links-Shift im peripheren Blutbild hinsichtlich der Neutrophilen sowie deutliche Veränderungen der Granulierung, der zytoplasmatischen Vakuolisierung und anderer Differenzierungsparameter.

In der Zusammenschau lassen sich die pharmakokinetischen und pharmakodynami-

schen Eigenschaften von Neupogen® als relativ gut charakterisiert bezeichnen, so dass sie für eine derartige Phase-I/II-Studie bei Patienten mit zerebraler Ischämie als ausreichend abgesichert gelten dürfen. Da die meisten Daten in gesunden Patienten aus der Mobilisierung von Stammzellen für die Knochenmarksspende hervorgegangen sind, gingen unsere Überlegungen auf die Dosierung in diesem Patienten- bzw. Probanden-Kollektiv zurück. Für die hiesige Studie werden aufgrund der noch nicht klar absehbaren Interaktionen aus möglicher spontaner Mobilisation von Leukozyten während der zerebralen Ischämie bzw. aus Neupogen®-induzierter Leukozytose eine Dosiseskalation von Neupogen® durchgeführt: es werden 3 Dosiskohorten mit jeweils 5 Patienten gebildet, wobei die erste Kohorte 2,5 µg/kg KG, die zweite Kohorte 5 µg/kgKG, und die dritte Kohorte 10 µg/kgKG täglich über 5 Tage s. c. erhält. Die Kohorte mit der höchsten Dosierung (10 µg/kgKG) erhält zunächst am Tag 1 und Tag 2 einschleichend 5 µg/kgKG und dann an den Tagen 3, 4, 5 jeweils täglich 10 µg/kg KG (Näheres siehe Punkt 7, Behandlungsplan und Dosisregime).

Literatur in:
**Morstyn G, Dexter TM, Food MA: Filgrastim (r-metHuG-CSF) in Clinical Practice. Marcel Dekker 1998**

## 1.6 Funktionelles Outcome und Lebensqualität

Das funktionelle Outcome wird mit Hilfe der modifizierten Rankin-Skala und des Barthel-Index evaluiert. Beide Skalen wurden wegen Ihrer Reliabiliät, ihrer großen Akzeptanz, ihrer guten Anwendbarkeit und schließlich wegen ihrer Verwendung in großen Schlaganfallakutstudien (NINDS, ECASS) gewählt. Die modifizierte Rankin-Skala ist eine 6-Punkteskala, die der allgemeinen Beurteilung der Unabhängigkeit dient. Der Barthel-Index wird zur Quantifizierung der Alltagsfunktionen wie Essen, Baden, Gehen, Treppensteigen, Anziehen, etc. herangezogen.

Zusätzlich bestimmen wir das Outcome mittels der Funktionellen Unabhängigkeitsskala (FIM=Functional Independance Measure). Die motorischen und kognitiven Komponenten der Skala werden zu Beginn und am Ende der Rehabilitationsphase erhoben. Die FIM-Skala ist eine 18-Punkte Skala, die die funktionelle Unabhängigkeit des Patienten mißt, einschließlich Essen, Kämmen, Anziehen, Toilettengang, Beweglichkeit und Kognition. Patienten erhalten einen Punktwert von 1 bis 7 für jeden Teilbereich der Skala, ein Punktwert von 126 zeigt völlige funktionelle Unabhängigkeit an. Die Skala demonstriert in den Studien eine hohe Validität, mißt auch geringe funktionelle Änderungen nach Schlaganfall und korreliert gut mit dem NIHSS Status, der nach einem Schlaganfall erhoben wird (Pallicino et al. 1992).

Die Beurteilung der Lebensqualität am Ende des Beobachtungszeitraumes erfolgt anhand der schlaganfallspezifischen Lebensqualitätsskala (SSQL). Die SSQL-Skala wurde von Williams et. al. entwickelt und 1999 in Stroke vorgestellt. Sie ist valide, reliabel und besteht aus insgesamt 12 Domänen bzw. 49 Fragen. Es können mindestens 49 und maximal 245 Punkte vergeben werden.

## 2. Studienziele

Da die Schlaganfall-bezogene Morbidität und Invalidität eine enorme sozioökonomische Belastung für unsere Volkswirtschaft darstellt, werden sowohl neue therapeutische Optionen für die Akutphase der Erkrankung als auch effektive Strategien für die Langzeitbehandlung mittels Neurorehabilitation dringend benötigt. Stammzell-basierte Therapiekonzepte könnten diesbezüglich eine potente nicht-invasive Behandlungstrategie für den akuten Schlaganfall darstellen.

Neben der thrombolytischen Therapie, die zunächst durch Revaskularisierung den initialen ischämischen Schaden reduziert und weiteren vaskulären und Gewebeschaden verhindert, könnten Stammzellen dazu beitragen, die zellulär basierten Reparaturmechanismen im Gehirngewebe zu beschleunigen und die funktionelle Erholung des Gehirns zu unterstützen.

Deshalb soll in der vorliegenden Studie untersucht werden, ob die Mobilisierung von Stammzellen aus dem Knochenmark mittels rhG-CSF zusätzlich dazu beitragen kann, (1) das funktionelle Outcome der Schlaganfallpatienten nach der Akuttherapie zu verbessern und (2) das endogene, Stammzellen-basierte Reparatursystem des ZNS nach ischämischen Ereignissen zu nutzen.

### 2.1. Primäres Studienziel

- Es soll die Verträglichkeit von rhG-CSF im Akutstadium des Schlaganfalles sowie die potentielle Toxizität der Medikation untersucht werden.

### 2.2. Sekundäre Studienziele

- Evaluierung der potentiellen funktionellen Verbesserung der Schlaganfallpatienten und Korrelation dieses funktionellen Gewinnes mit potentiellen morphologischen und perfusionsbedingten Änderungen im Läsionsgebiet.

- Evaluierung der Lebensqualität (SSQL) unter diesem Therapieprotokol

- Mögl. Morphologische Veränderungen im MRI und Veränderungen der zerebralen Perfusion

# 3. Studiendesign

## 3.1. Art der Studie

Es handelt sich um eine Dosis-eskalierte Phase I/II-Studie bei Patienten mit reperfundiertem akutem ischämischen A. cerebri media Teilinfarkt. Patienten mit inkomplettem Infarkt der Art. cerebri media (MCA) und mittelschwerem neurologischen Defizit (NIHSS 4-22) sollen mit rhG-CSF behandelt werden. Die Mobilisierung der Stammzellen aus dem Knochenmark und die Integration dieser Zellen in das ZNS soll im Rahmen eines standardisierten akutneurologischen und rehabilitativen Programmes mittels Bildgebung, funktioneller Bildgebung und laborchemischem Monitoring überwacht werden.

## 3.2. Patientenzahl

Im Rahmen dieser Studie sollen zunächst bezüglich des primären Zielparameters auswertbare Patienten behandelt werden. Insgesamt sollen 15 Patienten (3 x 5 Patienten) in diese Studie eingeschlossen werden, die ersten 5 mit einer niedrigeren Dosis (2,5 µg/kgKG), die zweiten 5 mit einer mittleren Dosis (5,0 µg/kgKG) und die nächsten 5 mit der Standarddosis (10 µg/kgKG), wobei bei der letzten Dosis von 10 µg/kgKG am Tag 1 und Tag 2 einschleichend 5 µg/kgKG s.c., dann an Tag 3, 4, 5 jeweils 10 µg/kg KG s.c. appliziert wird.

## 3.3. Zeitplan

Die Patientenrekrutierung soll vom 01.11.2002 bis 31.07.2003 erfolgen. Bei einer Nachbeobachtungszeit von 3 Monaten ist die gesamte Studie voraussichtlich spätestens im November 2003 beendet.

| | |
|---|---|
| **Studienpopulation** | Akuter ischämischer Hirninfarkt (Schlaganfall) mit moderaten neurologischen Ausfallserscheinungen (NIHSS 4-22) im Territorium der A. cerebri media (Media-Astverschluß, M2-Verschluß) , verifiziert durch CCT und/oder MRT |
| **Studiendesign** | • Prospektive klinische Phase I/II-Studie bei akuten Schlaganfall-Patienten<br>• Klinische Evaluation (NIH-Stroke Skala) nach 1, 7, 14, 28 Tagen sowie 3 Monaten<br>• Erfassung der Lebensqualität (SSQL)<br>• Funktionelles Outcome (Barthel, Rankin, FIM-Score)<br>• Klinische Chemie Tag 1-7, 14, 90<br>• Neuroradiologische Akutdiagnostik (CCT oder MRT)<br>• Morphologische Verlaufskontrollen (MRT): Tag 1-3, 7, 90 |
| **Patientenanzahl** | 15 Patienten in der Pilotphase: jeweils 5 Patienten mit niedriger (2,5 µg/kg KG), 5 Patienten mit mittlerer (5,0 µg/kg KG) und 5 Patienten mit hoher Dosierung (10,0 µg/kg KG) von G-CSF |
| | |
| **Behandlungsdauer** | 5 Behandlungen mit G-CSF (Neupogen$^{R}$ ) pro Patient, beginnend |

| | |
|---|---|
| | an Tag 1 nach Schlaganfall bis einschließlich Tag 5 |
| **Studiendauer** | Rekrutierungsphase: ca. 6 Monate<br>Beobachtungsintervall: ca. 3 Monate |

# 4. Patientenauswahl

## 4.1. Einschlusskriterien

- Akuter ischämischer Hirninfarkt (Schlaganfall) mit moderaten neurologischen Ausfallserscheinungen (NIHSS 4-22) im Territorium der A. cerebri media (Media-Astverschluß, M2-Verschluß)
- dominante und nicht-dominante Hemisphäre
- Aufklärungsfähigkeit der Patienten
- Studieneinschluß innerhalb 12 Stunden nach Beginn des Schlaganfallsymptome
- Alter ≥ 18 Jahre ≤ 65 Jahre (entgegen Fachinfo < 60 Jahre, da keine Leukapharese bei den Patienten durchgeführt wird)
- NIH-Stroke Scale 4-22 Punkte
- Normale Leberfunktion: GOT, GPT, γGT < 3-fache Normalwerte; Bilirubin < 1,5 mg/dl
- Normale Knochenmarksfunktion (Thrombozyten- Leukozytenzahlen im Normbereich)
- Compliance des Patienten
- Schriftliche Einwilligung (informed consent) des Patienten

## 4.2. Ausschlusskriterien

- Vorangegangene Behandlung mit Abciximab (Rheopro R)
- Vorangegangene thrombolytische Therapie innerhalb von 14 Tagen, nicht jedoch akute Lysetherapie des Schlaganfalles mit rtPA
- Teilnahme an anderer Studie
- globale Aphasie
- jegliche notwendige unverzügliche operative Intervention
- ulzerierende Karotis-Plaques, Pseudokklusion
- Hinweise auf akute Vaskulitis
- Dissektion arterieller Hirngefäße, ursächlich für das akute Schlaganfallereignis
- Vorangegangene Schlaganfallereignisse mit bleibender Behinderung (ischämisch oder hämorrhagisch)
- Medikation mit Immunsuppressiva (z.B. Glukokortikoide)
- Hämatologische Grunderkrankung (z.B: myeloproliferative Prozesse)
- Thrombozytenfunktionsstörungen
- Unbehandelte oder inadäquat behandelte metabolische Syndrome (Diabetes mellitus, Hyperlipidämie) oder nicht einstellbarer arterieller Hypertonus
- Gerinnungsstörungen
- schwere KHK
- Sichelzellanämie
- Jede andere schwere Grunderkrankung wie:
  - Gravierende psychiatrische Erkrankung (Depression, Psychose, Sucht)
  - Höhergradige Myokardinsuffizienz
  - positive HIV-Serologie
  - Malignom
- Allergie gegen G-CSF (Neupogen R)
- Schwangerschaft
- Nikotinabusus ≥ 20 Zigaretten/Tag

## 4.3. Patientenaufnahmeverfahren / Protokollfragen

Vor Aufnahme eines Patienten in die Studie führt der verantwortliche Arzt eine Überprüfung der Patientendaten bzgl. der Erfüllung der Ein- und Ausschlusskriterien des Untersuchungsplanes durch (siehe Synopsis). Das Aufklärungsgespräch mit dem Patienten wird in Anwesenheit eines Zeugen geführt, der den Aufklärungsbogen ebenfalls unterschreibt. Nur wenn der Patient aufklärungsfähig ist, wird ein Aufklärungsgespräch geführt. Es wird für die Patienten ein entsprechendes CRF nach FDA-Richtlinien geführt.

## 5. Prüfmedikation

Das vorliegende Protokoll einer klinischen Therapieoptimierung der Phase I/II wird mit der Handelsware Neupogen® durchgeführt. Hinweise zur Anwendung und zu möglichen Nebenwirkungen befinden sich in den jeweils gültigen Fachinformationen (s.u. Appendix). Rekobinantes Human-Granulocyte-Colony Stimulating Factor (G-CSF - Filgrastim, in Deutschland: Neupogen®) ist ein in Escherichia coli produziertes aus 175 Aminosäuren bestehendes Protein/Zytokin (Wong et al. 1985). Im Unterschied zu natürlichem G-CSF ist es nicht glykosyliert und besitzt ein zusätzliches N-terminales Methionin. Die hauptsächliche Wirkung von Filgrastim besteht darin, die Produktion neutrophiler Granulozyten zu stimulieren. Als Wachstumsfaktor erhöht es dementsprechend die Anzahl der im Blut zirkulierenden Vorläuferzellen. Darüber hinaus erhöht es die phagozytische und zytotoxische Funktion der neutrophilen Granulozyten. G-CSF verkürzt die Dauer einer schweren Neutropenie und verringert die Sekundärerkrankungsrate infolge von bakteriellen Infektionen und Mykosen (Hammond et al. 1989). Eine Filgrastim-Therapie kann auch den neutrophilen Granulozytenspiegel bei manchen Patienten mit Rückenmarksfehlbildungen oder -schädigungen (mässig schwere aplastische Anämien oder Tumorinfiltration ins Mark) verbessern.

Im Gegensatz zu G-CSF ist rhGM-CSF (Rekobinantes Human-Granulocyte Macrophage-Colony Stimulationg Factor (Sargramostin, in Deutschland: Molgramostim®) ein in Hefe produziertes, aus 127 Aminosäuren bestehendes Glykoprotein (Lee et al. 1985). Obwohl Sargramostin wie auch natürliches GM-CSF in Zellkulturen ein breites Aktivitätsspektrum aufweist, besteht seine primäre therapeutische Wirkung in der Stimulation der Myelopoese. GM-CSF ist in der Lage, die Proliferation, Differenzierung und Funktion verschiedener myeloischer Zellinien zu stimulieren. Darüberhinaus fördert es Migration, Phagozytose und Superoxidproduktion sowie die antikörperabhänige zellvermittelte Zytotoxizität von neutrophilen Granulozyten, Monozyten und eosinophilen Granulozyten, wobei auch die Produktion von TNF, IL-1 und M-CSF angeregt wird.

Filgrastim (G-CSF) wird als subkutane Injektion in einer Dosierung von 2,5-10 μg/kgKG pro Tag verabreicht. Die Verteilung im Plasma und die Plasmaclearance ist sowohl bei subkutaner als auch nach intravenöser Applikation bekannt und hat eine Halbwertszeit von 3,5 Stunden. Die Effektivität der Behandlung kann durch regelmässig erstellte Blutbilder kontrolliert werden. Als Dosis von Filgrastim wurden 2,5 bzw. 5 μg/kgKG bzw. 10 μg/kgKG pro Tag gewählt. G-CSF hat in diesen Dosierungen bisher keine dosislimitierenden Nebenwirkungen gezeigt.

| | |
|---|---|
| **Studienmedikation und Behandlungsplan** | • Studienmedikation: G-CSF (Neupogen R) 2,5 bzw. 5 bzw.10 μg/kg KG s.c. von Tag 1 bis einschl. Tag 5 nach Schlaganfall<br>• In der letzten Gruppe mit 10 μg/kg KG s.c. am Tag 1 und Tag 2 einschleichend Gabe von 5 μg/kg KG s.c., dann an Tag 3, 4, 5 Gabe von 10 μg/kg KG s.c.<br>• Fraktioniertes Heparin s.c. (Thrombembolie-Prophylaxe)<br>• Aspirin 100 mg/d und Clopidogrel 75 mg/d (Schlaganfall-Sekundärprävention)<br>• Symptomatische Therapie von arteriellem Hypertonus, Hypercholesterinämie, Diabetes mellitus, Infektionen etc.<br>• Standardisierte Physiotherapie, Logopädie und Ergotherapie |

# 6. Begleit- und Supportivmedikation

## 6.1 Basistherapie des akuten Schlaganfalls

### 6.1.1 Blutdruck-Management

- arterielle Hypotonie (< 140 mmHg syst.):
  - Volumensubstitution (HAES 6% i.v.)
  - Katecholamine (Dopamin, Dobutamin i.v.)
- arterelle Hypertonie (> 200 mmHg syst. und/oder > 110 mmHg diast.):
  - Nitrendipin 5-10 mg p.o. oder
  - Labetalol 10-20 mg i.v. oder
  - Urapidil 5-25 mg i.v.

### 6.1.2 Gewährleistung adäquater Oxygenierung

- kapilläre Hypoxygenierung ($O_2$- Sätt. < 95%):
  - 2-4 l $O_2$ über Nasensonde oder Maske
- Anämie:
  - Erythrozytenkonzentrate

### 6.1.3 Flüssigkeits- und Elektrolythaushalt

- Hypovolämie, Hyponatriämie, Hypokaliämie:
  - Flüssigkeits-, Elektrolytsubstitution

### 6.1.4 Glukosestoffwechsel

- Hypoglykämie (BZ < 70 mg%):
  - 25-50 ml Glukose 40% i.v.
- Hyperglykämie (BZ > 180 mg%):
  - Altinsulin 4 I.E. s.c. bzw. nach Insulinschema

### 6.1.5 Körpertemperatur

- ab 37,5°C Rektaltemperatur:
    - physikalische Kühlung
    - Antipyretika (Paracetamol, Metamizol)
- bei zusätzlichen Infektzeichen:
    - frühzeitige antibiotische Therapie

### 6.1.6 Allgemeine Prophylaxen

- Thromboseprophylaxe: Kompressionsstrümpfe, low-dose-Heparin s.c.
- Ulcusprophylaxe: Sucralfat oder $H_2$-Antagonisten
- Obstipationsprophylaxe: Lactulose, Laxantien
- Pneumonieprophylaxe: N-Acetylcystein
- Dekubitusprophylaxe: Lagerung

## 6.2 Indikationen zur PTT-wirksamen i.v. Heparinisierung in der Akutphase des Schlaganfalls

- Kardiale Emboliequelle:
    - (Vorhofflimmern, PFO, Herzklappendefekt bzw.-ersatz, intrakardiale Thromben)
- hochgradige symptomatische bzw. hämodynamisch relevante Karotisstenosen
- symptomatische intrakranielle Gefäßstenosen
- Dissektion der A. carotis oder A. vertebralis
- Koagulopathien:
    - (primäres Antiphospholipid-Antikörper-Syndrom, APC-Resistenz, Protein C-, S-Mangel)

## 6.2 Sekundärprävention nach Schlaganfall

| | |
|---|---|
| • TIA oder ischämischer Infarkt | ASS 100 mg/die und Clopidogrel 75 mg/die |
| • keine kardiale Emboliequelle | ASS 100 mg/die und Clopidogrel 75 mg/die |
| • keine höhergradige Karotisstenose | ASS 100 mg/die und Clopidogrel 75 mg/die |
| • Symptomatische höhergradige Karotisstenose (KS) | Thrombendarteriektomie + ASS 300 mg/die oder STENT-Anlage + ASS 300 mg/die bzw. Clopidogrel 75 mg/die oder Antikoagulation (INR 3-4) |
| • Stenose der A. carotis interna (<70%) | ASS 100 mg/die und Clopidogrel 75 mg/die |
| • Kardiale Emboliequelle | Antikoagulation (INR 3-4) |
| • nicht-rheumatischesVorhofflimmern | Antikoagulation (INR 3-4) |
| • intrakardiale Thromben | Antikoagulation (INR 3-4) |
| • Klappendefekt | Antikoagulation (INR 3-4) |
| • persistierendes offenes Foramen ovale (PFO) | ASS 100 mg/die und Clopidogrel 75 mg/die |
| • und Vorhof-Septum-Defekt | Antikoagulation (INR 3-4) oder Operation mit Transkatheterverschluß (PFO-Verschluss) |
| • Gerinnungsstörungen (z.B. Antiphospholipid-Antikörper -Syndrom, APC-Resistenz, Protein C-,S-Mangel) | Antikoagulation (INR 3-4) |
| • TIA oder ischämischer Infarkt und Myokardinfarkt oder pAVK | ASS 100 mg/die und Clopidogrel 75 mg/die |

# 7. Behandlungsplan und Dosierungsvorschriften

## 7.1. Übersicht über den Therapieplan

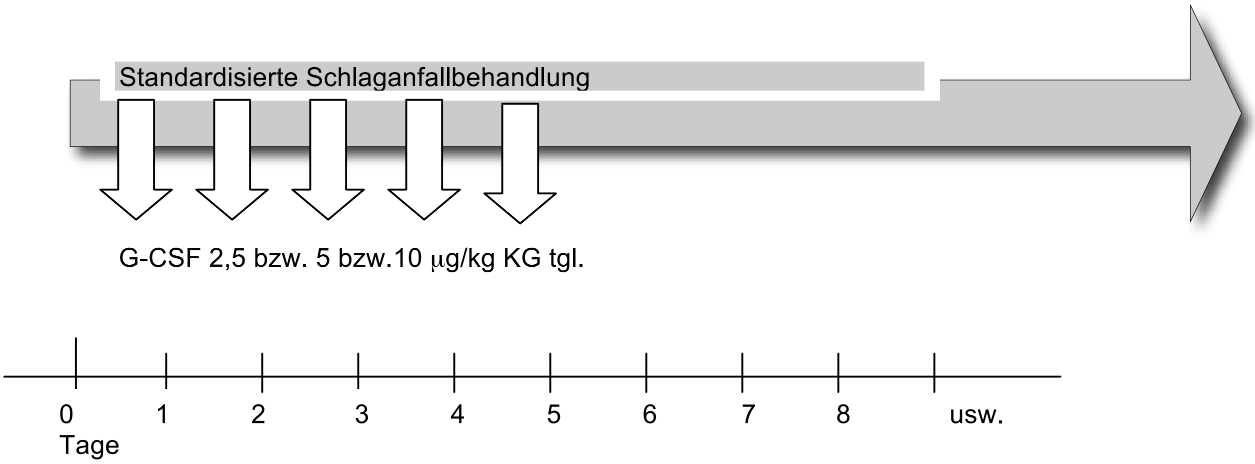


Da es sich um eine Phase I/II-Studie handelt, ist innerhalb des Patientenprotokolls eine Dosiseskalation vorgesehen: Aus Sicherheitsgründen werden daher den ersten 5 Patienten innerhalb von 5 Tagen täglich s. c. 2,5 µg/kg KG G-CSF Neupogen® verabreicht. In einer zweiten Kohorte werden den nächsten 5 Patienten 5,0 µg/kg KG G-CSF tgl. über 5 Tage verabreicht und anschliessend der dritten Kohorte 10,0 µg/kg KG G-CSF. In der letzten Gruppe mit 10 µg/kg KG s.c. am Tag 1 und Tag 2 einschleichend Gabe von 5 µg/kg KG s.c., dann an Tag 3, 4, 5 Gabe von 10 µg/kg KG s.c. Alle übrigen Bedingungen der Dosierung und des Therapieplanes gelten uneingeschränkt. Um eine zusätzliche Sicherheitsstufe für den Patienten in die Studie einzubauen, soll eine Dosisreduktion um 50% durchgeführt werden, sobald die Leukozytenzahl 20.000/µl überschreitet. Das übrige Studienprotokoll wird unverändert fortgeführt.

Die Dosiseskalation darf nur stattfinden, wenn keine schwerwiegende substanzbedingte Toxizität bei 3 von 5 Patienten der Kohorte unter der jeweiligen Prüfdosierung beobachtet wird. In kritischen Fragen bezüglich der Dosiseskalation entscheidet das externe Protokoll-Review-Kommitee. Selbstverständlich darf der Patient in keiner anderen Schlaganfallstudie rekrutiert sein, es darf auch zusätzlich keine weitere experimentelle Diagnostik und Therapie an dem Patienten vorgenommen werden. Eine akute Thrombolyse mit rtPA und Thrombozytenaggregationshemmung ist derzeit eine Standardtherapie des Schlaganfalles und deshalb erlaubt.

## 7.2. Therapiemodifikationen

Die Therapie wird gundsätzlich ohne Modifikationen wie oben beschrieben durchgeführt. Prinzipiell gelten die weiter unten und in der Studiensynopsis beschriebenen Abbruchkritterien für den einzelnen Patienten. Gegebenenfalls notwendige Änderungen der Therapie werden nur in Abstimmung mit dem externen Protokoll-Review-Kommitee vorgenommen und als Amendmend der Ethik-Kommission vorgelegt.

# 8. Untersuchungen

## 8.1. Zeitpunkte der Untersuchungen

- Am Tag des Einschlusses in das Studienprotokoll (komplette klinische Untersuchung und neurologische Untersuchung mit NIH-Stroke Skala)
- Klinische Untersuchung täglich während der Behandlungsphase (Tag 1 bis Tag 5), weiterhin an den Tagen 6, 7 und 14 sowie nach 3 Monaten (komplette klinische Untersuchung und neurologische Untersuchung mit NIH-Stroke Skala)
- Klinische Chemie täglich während der Behandlungsphase, weiterhin an den Tagen 6, 7, 14 und nach 3 Monaten
- Bestimmung der Lebensqualität nach 3 Monaten (SSQL)
- Neurospychologische Testung an Tag 7 und Tag 90
- Bestimmung der funktionellen Skalen (Barthel, Rankin) bei Einschluß, Tag 7 und 28 sowie Tag 90
- Bestimmung des funktionellen FIM-Score am Tag 7, 28 sowie Tag 90
- CCT- oder MRT-Bildgebung bei Aufnahme und Einschluß in die Studie
- MRT-Bildgebung an den Tagen 1-3, 7 und nach 3 Monaten

## 8.2. Umfang der Untersuchungen

- Ein-/Ausschlußkriterien
  entsprechend Prüfplan bzw. Datenerfassungsbögen (CRF)

- Anamnese
  - subjektive Beschwerden
  - relevante Vor-und Begleiterkrankungen
  - Vor- und Begleitmedikation
  - Beginn der neurologischen Symptomatik (Feststellen des Zeitfensters)
  - Zerebrovaskuläre Risikofaktoren (Nikotingenuss, arterielle Hypertonie, Diabetes mellitus, Hyperlipidämie)

- Klinische Untersuchung
  - untergliedert nach Organsystemen (neurologisch und internistisch)
  - internistische Untersuchung (Körpergröße, Gewicht, Blutdruck, Puls, Pumo, Cor, Abdomen, Fußpulse, Körpertemperatur)
  - neurologische Untersuchung

- Neurologisches Defizit
  - NIHSS (NIH Stroke Skala): eine 42-Punkte Skala, welche in großen Schlaganfallakutsudien eingesetzt wurde und die neurologische Beeinträchtigung in insgesamt 11 Kategorien beurteilt → siehe Anhang

- Funktionalität

- Barthel Index (BI): Beurteilung der Alltagsfunktionen wie Essen, Baden, Gehen, Treppensteigen, Anziehen, etc. (maximal 100 Punkte) → siehe Anhang

- Modifizierte Rankin Skala (RS): Beurteilung der Unabhängigkeit (0-2 = Patient unabhängig, 3 = Patient gehfähig, 4-5 = Patient rollstuhlpflichtig bzw. bettlägerig) → siehe Anhang

- Funktionelle Unabhängigkeits-Score (FIM=Functional Independance Measure): die motorischen und kognitiven Komponenten der Skala werden zu Beginn und am Ende der Rehabilitationsphase erhoben. Der FIM-Score ist eine 18-Punkte Skala, die die funktionelle Unabhängigkeit des Patienten misst, einschließlich Essen, Kämmen, Anziehen, Toilettengang, Beweglichkeit und Kognition. Patienten erhalten einen Punktwert von 1 bis 7 für jeden Teilbereich der Skala, ein Punktwert von 126 zeigt völlige funktionelle Unabhängigkeit an → siehe Anhang

- Lebensqualität
  - Beurteilung anhand der SSQL (stroke-specific quality of life scale), eingeführt von Williams et. al. (Stroke 1999), besteht aus insgesamt 12 Domänen und 49 Fragen wobei mindestens 49 und maximal 245 Punkte zu vergeben sind → siehe Anhang

- Neuropsychologie
  - Neuropsychologische Testung nach 7 Tagen und 3 Monaten → siehe Anhang

- Laborparameter
  - Initial: Differentialblutbild, G-CSF, BSG, CRP, Elektrolyte (Na+, K+, Ca++), Kreatinin, Harnstoff-N, Leberwerte (Bilirubin, AP, GGT, GPT, GOT), CK, Glukose, Gesamtcholesterin, Triglyceride, HDL, LDL, INR, PTT, ausführliche Gerinnung, Schwangerschaftstest, HIV-Serologie
  - Follow-up: Differentialblutbild, G-CSF, BSG, CRP, Elektrolyte (Na+, K+, Ca++), Kreatinin, Harnstoff-N, Leberwerte (Bilirubin, AP, GGT, GPT, GOT), CK, Glukose, INR, PTT, ausführliche Gerinnung
  - Zytokin-Bestimmung: eine $CD^{34}$ und AC 133 – Bestimmung erfolgt an den Tagen 0, 4, 7,28 und Tag 90

- Neuroradiologie
  - CCT/CTA oder MRT/MRA am Einschlusstag in die Studie: akute Schlaganfallpatienten mit M2-Segment Verschluß im MCA (Art. cerebri media)-Stromgebiet
  - MRT: T1, T2, Flair, Diffusion, ADC, Perfusion (Tag 1-3, Tag 7 und Tag 90)
  - MP-Rage: T1-gewichtet 3-D-GE Sequenz axial Flair, axial DWI und ADC, koronar T1, axial Perfusion, TOF-MRA

## 8.3. Tabellarische Auflistung des Untersuchungsumfanges

| **Untersuchung** | **Einschluß** | | | | | | | | | |
|---|---|---|---|---|---|---|---|---|---|---|
| **Tag der Unersuchung** | **0 / 1** | **2** | **3** | **4** | **5** | **6** | **7** | **14** | **28** | **90** |
| **Ein-/Ausschlußkriterien** | **+** | | | | | | | | | |
| **Einverständniserklärung** | + | | | | | | | | | |
| **Anamnese** | + | | | | | | | | | |
| **Klinische Untersuchung** | | | | | | | | | | |
| Intern. (incl.RR, Puls, Temp) | + | + | + | + | + | + | + | + | + | + |
| Neurologisch (incl.Skalen) | + | + | + | + | + | + | + | + | + | + |
| **Funktionalität** | | | | | | | | | | |
| NIHSS | + | + | + | + | + | + | + | + | + | + |
| Barthel-Index | + | | | | | | + | | + | + |
| Modified Rankin Scale | + | | | | | | + | | + | + |
| FIM | | | | | | | + | | + | + |
| **Lebensqualität** | | | | | | | | | | |
| SSQL | | | | | | | | | | + |
| **Neuropsychologie** | | | | | | | | | | |
| Testpsychologie | | | | | | | + | | | + |
| **Laboruntersuchungen** | | | | | | | | | | |
| Hämatologisch* | + | + | + | + | + | + | + | + | + | + |
| Klnisch-chemisch** | + | + | + | + | + | + | + | + | | |
| Schwangerschaftstest*** | + | | | | | | | | | |
| CD$^{34}$ / AC 133 | + | | | + | | | + | | + | + |
| **Zusatzuntersuchungen** | | | | | | | | | | |
| EKG | + | | | | | + | | | | |
| Duplexsonographie | + | | | | | + | | | | |
| Körpertemperatur | + | + | + | + | + | + | + | + | + | + |
| **Neuroradiologie**** ** | | | | | | | | | | |
| CCT / CTA | + | | | | | | | | | |
| MRT / MRA | + | | | | | | + | | | + |

* Differentialblutbild, Zytokine, G-CSF

** BSG, CRP, Elektrolyte (Na+, K+, Ca++), Kreatinin, Harnstoff, Leberenzyme (Bilirubin, AP, GGT, GPT, GOT),CK, Glucose, Gesamtcholesterin, Triglyceride, HDL, LDL, INR, PTT, HIV-Serologie, ausführlicher Gerinnungsstatus

*** bei Frauen < 50 Jahre

**** Falls am Einschlusstag ein CCT durchgeführt wurde, wird am Tag 1 – 3 das MRT/MRA durchgeführt

## 9. Therapiebewertung

Die Bewertung der Therapie erfolgt nach folgenden Gesichtspunkten:

**Primäre Endpunkte**

- Toxizität und Tolerabilität von G-CSF bei Patienten mit akutem Schlaganfall

| | |
|---|---|
| **Kriterien für Toxizität -**<br>**"adverse events"** | • Anhaltendes Fieber > 38,5 $^0$C<br>• Thrombozytose > 500,000 /µl mit klinisch-neurologischer oder systemischer Verschlechterung<br>• Schwere anhaltende Leukozytose > 50,000 /µl mit klinisch-neurologischer oder systemischer Verschlechterung<br>• Jegliche Art der ZNS-Störung die normalerweise nicht im Zusammenhang mit einem Schlaganfall im Mediastromgebiet zu erklären ist<br>• Wiederholte kleine oder große Schlaganfälle innerhalb der 14tägigen Studienperiode, nicht erklärt durch die individuelle Pathophysiologie des Patienten<br>• Makroskopische ZNS Hämorrhagien innerhalb von 14 Tagen nach Studieneinschluss<br>• Jegliche Art von systemischer Toxizität nicht erklärbar durch vorherige toxikologische Studien |

**Sekundäre Endpunkte**

- Funktionelles Outcome bei Patienten mit akutem Schlaganfall nach Gabe von G-CSF: wiederholte Erhebung der NIH-Stroke Skala, der modifizierten Rankin-Skala, des Barthel-Index und des FIM-Score
- Morphologische Veränderungen im MRT (MR-voxel-directed morphometry) zwischen Tag 1-3, Tag 7 und Tag 90 nach Hirninfarkt
- Lebensqualität SSQL am Ende des Beobachtungszeitraumes

## 10. Sicherheit (Toxizität)

G-CSF wird in dieser Studie zur Mobilisierung von Stammzellen bei Schlaganfallpatienten eingesetzt. Hierüber gibt es bisher keine klinischen Daten in der Literatur. Allerdings kann man wohl davon ausgehen, dass ein ähnliches Toxizitätsprofil besteht, wie es bei der Mobilisierung von Stammzellen in gesunden Spendern zu erwarten ist. Die am häufigsten beobachteten unerwünschten Nebenwirkungen sind leichte bis mäßige, vorübergehende Knochenschmerzen sowie Schmerzen der Skelettmuskulatur. Bei 41 % der Spendern wurde bei einer G-CSF Dosierung von 10 µg/kg KG s.c. eine Leukozytose über 50,000/µl beobachtet. Da eine Leukapherese nicht vorgesehen ist, ist möglicherweise von der sonst beobachteten Thrombozytopenie in Kombination von G-CSF-Gabe und Leukapherese nicht auszugehen.

Über vorübergehende geringfügige Erhöhung der Spiegel der alkalischen Phosphatase, der Laktatdehydrogenase, der SGOT sowie der Harnsäure wurde bei gesunden Spendern, die NEUPOGEN® erhalten hatten, berichtet. Diese blieben ohne klinische Folgen. Selten wurde eine Verschlechterung von arthritischen Symptomen beobachtet. Über Symptome, die auf allergische Reaktionen hinweisen, wurde sehr selten berichtet. In Studien mit peripheren Blutstammzellspendern, wurde auch über Kopfschmerzen berichtet, die möglicherweise durch NEUPOGEN® verursacht wurden. Einzelheiten des Toxizitätsprofiles von NEUPOGEN® im Einsatz bei gesunden Spendern sind dem folgenden zu entnehmen:

In mehreren Publikationen von Studien zum Einsatz von NEUPOGEN® bei gesunden Spendern (Kinder und Erwachsene) wurde auf das Sicherheitsprofil von NEUPOGEN® eingegangen. NEUPOGEN® wurde bei den insgesamt 235 gesunden Spendern, die hier beschrieben sind, gut vertragen, wobei Knochenschmerzen das Ereignis war, das am häufigsten angeführt wurde.[1-12]

Aus der Literatur sind Einzelfälle über unerwünschte Ereignisse bei gesunden Spendern bekannt, wie z.B. Milzruptur, anaphylaktoide Reaktion, Gichtarthritis, Schlaganfall, Vaskulitis oder Angina pectoris.

Von Bedeutung ist eine von Becker et al. berichtete Milzruptur bei einem gesunden Spender mit kräftiger Muskulatur, bei dem keine Splenomegalie palpabel war und bei dem während einer 6-tägigen Gabe von NEUPOGEN® zur Mobilisierung von peripheren Blutstammzellen Fieber, Kopfschmerzen, Übelkeit und Erbrechen auftraten[13]. Der Patient erhielt nicht genauer spezifizierte Analgetika und Antiemetika, die Apherese wurde an den Tagen 6 und 7 abgeschlossen; die maximale Leukozytenzahl betrug 35.600/µl. 12 Stunden nach Entfernung des Apheresekatheters aus der rechten Subclavia entwickelte der Patient an Tag 8 nach einer Episode starken Erbrechens einen symptomatischen rechtsseitigen Pneumothorax, und eine Pleuradrainage wurde gelegt. An Tag 11, also 4 Tage nach Beendigung der NEUPOGEN®-Gabe, wurden bei dem Patienten eine Splenomegalie und eine Milzruptur bei einem Hämatokrit von 18,9% festgestellt. Daraufhin wurde eine Splenektomie durchgeführt; im Pathologiebericht fand sich der Hinweis auf extramedulläre Blutbildung, Epstein-Barr-Virus (EBV) konnte im Milzgewebe nicht nachgewiesen werden. Als postoperative Komplikation trat ein ARDS auf, der Patient erholte sich nach entsprechender Behandlung und wurde am 10. Tag nach der Operation entlassen.

Margolis et al. beschreiben, in einem als Abstract veröffentlichten Fallbericht, das Auftreten einer Milzruptur bei einem 43-jährigen gesunden Stammzellspender, bei dem sich am sechsten Tag der Stammzellmobilisierung mit NEUPOGEN® nach Abbruch der Leukapherese im CT perihepatische und perisplenisches Blut bei normaler Milzgröße zeigte. Eine Milzruptur wurde diagnostiziert. Der Spender benötigte Erythrozyten- und Thrombozyten-Konzentrate und konnte nach Verbesserung seines Zustades ohne Operation vier Tage nach dem Ereignis entlassen werden[19].

Adkins et al. erwähnen einen 16 Jahre alten gesunden Spender, der 50 Minuten nach der ersten subkutanen Injektion von NEUPOGEN® 10 μg/kg KG zur Mobilisierung von peripheren Blutstammzellen Anzeichen bzw. Symptome einer anaphylaktoiden Reaktion zeigte. Als Symptome wurden Kurzatmigkeit und starke schmerzhafte Krämpfe im Unterbauch beobachtet. Der Blutdruck sank von 110/70 mmHg bei der Eingangsuntersuchung auf 80/50 mmHg, der Puls stieg von 88 auf 140/min an; die Atemfrequenz lag bei 28/min. Die Lunge war bei der Auskultation frei. Eine intravenöse Flüssigkeitssubstitution, sowie eine Behandlung mit H1- und H2-Rezeptorenblockern und Kortikosteroiden wurde umgehend eingeleitet. Obwohl der systolische Blutdruck wieder anstieg und die Atemnot verschwand, litt der Patient weiterhin unter Schmerzen im Unterbauchbereich. Es wurden dann Meperidin und Epinephrin gegeben, woraufhin die Schmerzen im Unterbauch innerhalb von 10 Minuten verschwanden. Zwei Stunden nach der NEUPOGEN®-Injektion kam es bei dem Patienten zu Blasenkrämpfen und Hämaturie; im Urin fanden sich kleine Blutgerinnsel. Am nächsten Tag waren bei dem Spender Anzeichen von Schwäche zu beobachten. Die Autoren vermuteten wegen des zeitlichen Bezuges zur NEUPOGEN®-Gabe und des Fehlens anderer Ursachen einen Kausalzusammenhang zwischen der anaphylaktoiden Reaktion und NEUPOGEN®.

Spitzer et al. berichten über einen gesunden Spender mit degenerativer Arthritis im rechten Knie mit Z.n. Knieersatz, der nach 5 Tagen, an denen ihm Filgrastim zur Mobilisierung der peripheren Blutstammzellen verabreicht worden war, eine Gichtarthritis im linken Fuß bekam[15]. Bei dem Patienten war vorher keine Gicht aufgetreten, und bei der eingangs durchgeführten körperlichen Untersuchung zeigte er ebensowenig Gicht- oder Arthritissymptome; bei dieser Untersuchung lag der Serumharnsäurespiegel bei 8,2 mg/dl. Symptome von Druckempfindlichkeit, Erythem, Schwellung sowie Beweglichkeit über der Ferse wurden einer entzündlichen Hautreaktion (Cellulitis) zugeschrieben, und der Patient wurde zunächst mit Breitspektrumantibiotika behandelt. Es wurde keine weitere Bestimmung des Harnsäurespiegels mehr vorgenommen. Bei einer Nadelaspiration wurden Uratkristalle festgestellt, daraufhin wurde Colchicin gegeben, was zu einer Besserung der Symptome führte. Die Autoren gehen davon aus, dass der Spender aufgrund seines hohen Harnsäurespiegels und einer Aspirintherapie eine Veranlagung zur Entwicklung einer Gichtarthritis hatte; die schnelle Zunahme der myeloischen Stammzellen sowie der erhöhte Zellumsatz durch Filgrastim lösten möglicherweise die Sehnenentzündung und die akute Gichtarthritis aus.

Baker et al. beschreiben einen Fall eines älteren Stammzellspenders, mit vorbestehender Hypertension und Angina pectoris. Dieser entwickelte eine Verschlechterung seiner intellektuellen Leistungsfähigkeit und Mobilität einige Tage nach der 3. Stammzellapherese nach Mobilisierung mit NEUPOGEN®[16].Daran anschließend erlitt der Patient einen rechtsseitigen Schlaganfall und verstarb 2 Wochen später. Die maximale Leukozytenzahl betrug 43.500/µl. Der Patient hatte zuvor nur über Knochenschmerzen geklagt. Die Autoren ziehen den Schluss, dass Spender, welche Ausschlusskriterien für eine Vollnar-

kose zur Knochenmarkspende haben, auch gleichzeitig einem erhöhten Risiko bei der Stammzellmobilisierung ausgesetzt sind.

Vaz et al. berichten über einen gesunden jugendlichen Spender, welcher 2 Monate nach Mobilisierung mit NEUPOGEN® eine lymphozytische Vaskulitis entwickelte, welche sich über Hautläsionen manifestierte und durch eine Biopsie bestätigt werden konnte.[17] Das Blutbild war normal und ein immunologisches Screening unauffällig. Die Hautläsionen heilten ohne Behandlung ab.

In einem „Brief an den Herausgeber" wurde über einen gesunden Spender mit einer Anamnese von Nikotinabusus, einem zweiwöchigen leichten Belastungsbrustschmerz sowie mit einer Familienanamnese einer Herzgefäßerkrankung berichtet. Dieser Patient entwickelte 6 Stunden nach der zweiten NEUPOGEN®-Gabe zur Stammzellmobilisierung Palpitationen und Brustschmerz. Die Leukozyten waren von 6.900/µl auf 27.100/µl gestiegen. Der EKG-Befund zeigte eine Trigeminie und neue kleine Q-Zacken in der Ableitung II sowie Vorhofflimmern und T-Wellen-Inversion in Ableitung I und II. Die NEUPOGEN®-Behandlung wurde unterbrochen und der Spender wurde hospitalisiert. Troponin, PT- und PTT- Werte waren innerhalb des Normbereichs. Ein erneutes EKG 7 Stunden später zeigte andauernde Q-Zacken und T-Wellen Veränderungen. Am darauffolgenden Tag wurde die dritte NEUPOGEN®-Dosis verabreicht. Nach 6 Stunden kam es zu einem Wiederauftreten der Symptome. NEUPOGEN® wurde abgesetzt. Nachfolgende Herzkatheterisierung zeigte eine signifikante koronare Herzkrankheit mit einem 100%igen Verschluss der rechten Koronararterie sowie eine 100%igen Verschluss eines septalen und diagonalen Astes der linken Koronararterie. Des weiteren wurde eine 50%ige Okklusion der linken Koronararterie festgestellt. Der Spender erhielt eine perkutane transluminale koronare Angioplastie, während der ein Stent in die rechte Koronararterie eingesetzt wurde. Zwei Monate später wurde das Spenderknochenmark unter Vollnarkose ohne Komplikationen entnommen. Die Autoren bemerkten, dass obwohl der Einfluss von NEUPOGEN® auf die kardiale Ischämie des Spenders unklar ist, der zeitliche Zusammenhang zwischen der NEUPOGEN®-Gabe und dem Beginn der kardialen Ischämie einen potentiellen Zusammenhang nahe legt, möglicherweise als Resultat einer erhöhten Thrombozytenaggregation. Des weiteren regten die Autoren an, dass Stammzellspender mit Anamnese oder Symptomen einer Herzgefäßerkrankung kein G-CSF erhalten sollten. Sollte die Applikation trotzdem erfolgen, muss die kardiale Funktion eines solchen Spenders engmaschig kontrolliert werden.

G-CSF wurde in vielen klinischen Studien generell gut toleriert. Auch wenn rhG-CSF beim Menschen eventuell die Koagulationskaskade beeinflusst, so zeigten sich jedoch keinerlei schädlichen Auswirkungen oder Verträglichkeitsprobleme unter einer Therapie mit rhG-CSF, was anhand einer Follow-up Studie bei Patienten über einen Zeitraum von drei Jahren untersucht wurde (mittlere rhG-CSF Dosis 16 µg/kgKG über einen Median von 6 Tagen - Cavallaro et al. 2000). Es gibt bereits zahlreiche Verträglichkeitsstudien von rhG-CSF in Patienten mit hämatopoetischen und onkologischen Grunderkrankungen. Diese Untersuchungen ergeben keinen Hinweis darauf, dass nach rhG-CSF Applikation das Risiko für einen Schlaganfall ansteigt. Dosisabhängige Nebenwirkungen mit Fieber und Knochenschmerzen sind in der Literatur beschrieben und treten in ca. 20% der behandelten Patienten auf. Diese Nebenwirkungen können sehr einfach durch Gabe von Paracetamol kontrolliert oder verhindert werden. Bei hohen und über längere Zeit verabreichten G-CSF Dosen können schwache bis moderate Knochenschmerzen und bei subkutaner Injektion lokale Hautreaktionen und selten eine dermale nekrotisierende

Vaskulitis auftreten. Weist die Anamnese eine Überempfindlichkeit des Patienten für ein E. coli-Produkt aus, sollte auf eine Anwendung von G-CSF verzichtet werden.

## 10.1. Erfassung und Dokumentation von AEs, SAE´s und ADR´s

(1) Alle unerwünschten Ereignisse werden kontinuierlich während der Verlaufsbeobachtung und bei jeder Kontrollvisite registriert und entsprechend der CTC Skala Version 2.0 bewertet.

(2) Der Zusammenhang des unerwünschten Ereignisses (AE) mit der Applikation des Prüfmedikamentes sollte entsprechend den Klassifikationsmerkmalen der CTC beurteilt werden:

| Code | Bezeichnung | Definition |
|---|---|---|
| 5 | gesichert (definite) | Das AE hat einen klaren/deutlichen Zusammenhang (clearly related) mit der Prüfsubstanz. |
| 4 | wahrscheinlich (probable) | Das AE hat einen warscheinlichen/ glaubhaften Zusammenhang (likely related) mit der Prüfmedikation. |
| 3 | möglich (possible) | Das AE könnte einen Zusammenhang (may be related) mit der Prüfsubstanz haben. |
| 2 | unwarscheinlich (unlikely) | Der Zusammenhang zwischen AE und Prüfsubstanz ist zweifelhaft (doubtfully related) |
| 1 | kein (not related) | Das AE hat klar/deutlich keinen Zusammenhang (not related) mit der Prüfsubstanz. |

Jedes unerwünschte Ereignis sollte genau überwacht werden, bis die Beschwerden abgeklungen sind.

## 10.2. Unerwünschte Ereignisse – Adverse Events (AE)

Unerwünschte Ereignisse sind alle während einer klinischen Prüfung bei einer Versuchsperson beobachteten klinischen, labordiagnostischen, instrumentell-diagnostischen etc. Veränderungen, unabhängig von einem möglichen kausalen Zusammenhang mit der Behandlung der Prüfsubstanz. Der Prüfarzt muss daher alle unerwünschten Ereignisse in einen Prüfbogen eintragen, auch wenn sie nicht arzneimittelbezogen sind, einschließlich der bekannten Nebenwirkungen handelsüblicher Produkte.

### 10.2.1 Meldung von AE´s

Nicht schwerwiegende unerwünschte Ereignisse werden im CRF dokumentiert.

## 10.3. Schwerwiegende unerwünschte Ereignisse - Serious Adverse Events (SAE)

Im folgenden eine typische Klassifizierung von schwerwiegenden unerwünschten Ereignissen. Das Ereignis wird als schwerwiegend eingestuft, wenn die Versuchsperson:

- verstirbt
- in eine lebensbedrohliche Situation gerät
- stationär behandelt werden oder längere Zeit im Krankenhaus verbleiben muss
- durch anhaltende oder starke Körperbehinderung oder Individualität geschädigt wird
- Nachkommen mit angeborener Anomalie oder Missbildungen bekommt
- ein bedeutendes medizinisches Ereignis (wie im Prüfplan definiert) eintritt, wie z.B. Überdosierung, Schwangerschaft oder Krebs.

Beim Ereignis “Tod” sollte immer die Todesursache ermittelt (möglichst Sektion) und dokumentiert werden. Falls die Todesursache unbekannt ist und trotz sorgfältiger Recherche unbekannt bleibt, kann der Ausdruck “unerklärbarer Tod” (“unexplained death”) verwendet werden, um die Reaktion zu beschreiben.

### 10.3.1 Meldung von SAE´s

Schwerwiegende unerwünschte Ereignisse (SAE) müssen durch den jeweils verantwortlichen Arzt innerhalb von 24 Stunden nach Bekanntwerden dem Studienleiter und dem Referat Arzneimittelsicherheit der Herstellerfirma des mit dem SAE in Zusammenhang stehenden Arzneimittels gemeldet werden.

Leiter der klinischen Prüfung:

**Prof. Dr. med. U. Bogdahn**

Klinik und Poliklinik für Neurologie der Universität Regensburg im Bezirksklinikum
Universitätsstrasse 84
93053 Regensburg
Tel.: 0941 / 9 41 3001
Fax: 0941 / 9 41 3205

Folgende Minimalkriterien müssen bei schwerwiegenden Nebenwirkungen in jedem Fall dokumentiert werden:

- Patient: Mindestens zwei Angaben zur Identifikation (Patientennummer, Geburtsdatum, Initialen, Geschlecht), z. B. ”Patientennummer 56, *27.05.40, A.B., weiblich”
- Arzneimittel: Präparat oder Wirkstoff; z. B. ”Präparat X”
- Reaktion: Symptom; z. B. ”Milzruptur”

- Datenquelle: Studiennummer, Studienzentrum, Arzt; z. B. CF456, Zentrum Y, Arzt W (Telefonnummer)

Die zuständige Ethikkommission muss über alle Ereignisse, die während der Studie auftreten und die Sicherheit der Studienteilnehmer oder die Durchführung der Studie beeinträchtigen könnten, informiert werden. Sie bewertet, ob eine erneute Prüfung der ethischen Aspekte notwendig erscheint. Die Meldung an die Ethikkommission erfolgt durch den Leiter der klinischen Prüfung.

Sofern erforderlich, werden die Arzneimittelbehörden informiert.

Der Name der Berichtenden und anderer Beteiligter wird nur mit schriftlichem Einverständnis des Berichtenden bekannt gegeben. Die Anonymität der Patientenidentität wird gewährleistet.

## 10.4. Unerwünschte Arzneimittelwirkung - Adverse Drug Reaction (ADR)

Wenn bei einem unerwünschten Ereignis der Verdacht besteht, dass die Studienmedikation die Ursache hierfür ist, muss dieses Ereignis als ADR betrachtet werden. Ein ADR ist schädlich und unbeabsichtigt. Sie tritt auf bei normaler Dosierung, die als sicher angenommen wird.

Eine Überschreitung der lt. Protokoll vorgeschriebenen Dosierung sowie Überdosierung, Missbrauch, Abhängigkeit und Wechselwirkung mit anderen medizinischen Produkten müssen als unerwünschte Arzneimittelwirkung betrachtet werden.

### 10.4.1 Meldung von ADR´s

Nicht-schwerwiegende ADR´s werden im CRF dokumentiert.

**Referenzen zur Toxitität**


1. Anderlini P, Körbling M, Dale D, Gratwohl A, Schmitz N, Howe C, Leitman S, Horowitz M, Gluckman E, Rowley S, Przepiorka D, Champli R. Alogeneic Blood Stem Cell Transplantation: Considerations for Donors. *Blood*. 1997; 90(3):903-908

2. Anderlini P, Przepiorka D, Seong D, Miller P, Sundberg J, Lichtiger B, Norfleet F, Chan KW, Champlin R, Korbling M. Clinical toxicity and laboratory effects of granulocyte colony-stimulating factor (filgrastim) mobilization and blood stem cells apheresis from normal donors, and analysis of charges for the procedures. *Transfusion*. 1996; 36:590-595

3. Anderlini P, Przepiorka D, Lauppe J, Seong D, Giralt S, Champlin R, Korbling M. Collection of peripheral blood stem cells from normal donors 60 years of age or older. *British Journal of Hematology*. 1997; 485-487.

4. Arbona C, Prosper F, Benet I, Mena F, Solano C, Garcia-Conde J. Comparison between once a day vs twice a day G-CSF for mobilization of peripheral blood progenitor cells (PBPC) in normal donors for allogeneic PBPC transplantation. *Bone Marrow Transplantation*. 1998; 22:39-45.

5. Gonzalez M, Benito A, Diaz MA, Madero L. Peripheral blood progenitor cell (PBPC) collection by large-volume leukapheresis from pediatric donors [letter]. *Bone Marrow Transplantation*. 1999; 23:631-632.

6. Kawano Y, Takaü Y, Watanabe T, Watanabe A, Iwai A, Kuroda Y. Peripheral blood stem cell mobilization with granulocyte colony-stimulating factor and a harvesting procedure in pediatric donors. *Bone Marrow Transplantation*. 1998; 21(S3):S32-S34.

7. Ordemann R, Holig K, Wagner K, Rautenberg U, Bornhauser M, Kroschinsky F, Schafter J, Schuler U, Ehninger G. Acceptance and feasibility of peripheral stem cell mobilisation compared to bone marrow collection from healthy unrelated donors. *Bone Marrow Transplantation*. 1998; 21(S3):S25-S28.

8. Stroncek DF, Clay ME, Smith J, Ilstrup S, Oldham F, McCullough J. Changes in blood counts after the administration of granulocyte colony-stimulating factor and the collection of peripheral blood stem cells from healthy donors. *Transfusion*. 1996; 36:596-600.

9. Stroncek DF, Clay ME, Smith J, Ilstrup S, Oldham F, McCullough J. Treatment of normal individuals with granulocyte colony-stimulating factor: donor experiences and the effects on peripheral blood CD 34+ cell counts and on the collection of peripheral blood stem cells. *Transfusion*. 1996; 36:601-610.

10. Waller CF, Bertz, H, Wenger MK, Fetscher S, Hardung M, Engelhardt M, Behringer D, Lange W, Mertelsmann R, Finke J. Mobilization of peripheral blood progenitor cells for allogeneic transplantation: efficacy and toxicity of a high-dose rhG-CSF regimen. *Bone Marrow Transplantation.* 1996; 18:279-283.

11. Majolino I, Cavallaro AM, Bacigalupo A, Rambaldi A, Falda M, Locatelli F, Faimondi R, Pucci G, Mordini N, Barbui T, Rodeghiero F, Irrera G, Indovina A, Valbonesi M, Bellavita P, Tassi V, Perotti C, Soli M, Bresolin G. Mobilization and collection of PBSC in healthy donors: a retrospective analysis of the Italian Bone Marrow Transplantation Group (GITMO). *Hematologica*. 1997; 82:47-52.

12. Tabilio A, Falzetti F, Giannoni C, Aversa F, Martelli MP, Rossetti M, Caputo P, Chionne F, Gambelunghe C, Martelli MF. Stem cell mobilization in normal donors. *Journal of Hematotherapy*. 1997; 6:277-234.

13. Becker PS, Wagle M, Matous S, Swanson RS, Pihan G, Lowry PA, Stewart FM, Heard SO. Spontaneous splenic rupture following administration of granulocyte colony-stimulating factor (G-CSF): Occurrence in an allogeneic donor of peripheral blood stem cells. *Biology of Blood and Marrow Transplantation*. 1997; 3:45-49.

14. Adkins DR. Anaphylactoid reaction in a normal donor given granulocyte colony-stimulating factor [letter]. *Journal of Clinical Oncology*. Feb 1998; 16(2):812-813.

15. Spitzer T, McAfee S, Poliquin C, Colby C. Acute gouty arthritis following recombinant human granulocyte colony-stimulating factor therapy in an allogeneic blood stem cell donor [letter]. *Bone Marrow Transplantation*. 1998; 21:966-967.

16. Baker D, Durrant S. Fatal stroke following Filgrastim mobilisation of PBPC for allogeneic transplantation. Abstract presented at the *25th Annual Meeting of the European Group for Blood and Marrow Transplantation*. 1999.

17. Vaz CP, Correia O, Henrique R, Campilho F, Campos A, Pimentel P. Recombinant granulocyte colony-stimulating factor (rhG-CSF) associated vasculitis during stem cell mobilization. *Bone Marrow Transplantation*. 1999; 23(S1):abstract 266.

18. Vij R, Adkins DR, Brown RA, Khoury H, DiPersio JF, Goodnough T. Unstable angina in a peripheral blood stem and progenitor cell donor given granulocyte colony-stimulating factor [letter]. *Transfusion*. 1999; 39:542-543.

19. Margolis D, Dincer A, Anderson L, Moraski L, Casper J, Gottschall J. Serious Adverse Events in Parental Donors Undergoing Peripheral Blood Progenitor Cell Mobilization: A Trigger for Changing the Donation Process. *43rd Annual Meeting of American Society of Hematology*, Orlando, Florida, December 7-11, 2001, abstract no. 750

Stand November 2001

## 11. Vorzeitiges Studienende

### 11.1. Für den Patienten

Der Patient kann zu jeder Zeit, ohne Angabe von Gründen, aus der Untersuchung ausscheiden. Ein Behandlungsnachteil darf ihm hieraus nicht entstehen.

Die Studie muß abgebrochen werden wenn in 3 von 5 Patienten der jeweiligen Kohorte ein schweres "adverse event" oder Tod auftritt.

Der Abbruch der Therapie für den einzelnen Patienten erfolgt bei folgenden Kriterien:

| | |
|---|---|
| **Kriterien für einen Studienabbruch** | Die Studie muß abgebrochen werden bei einem individuellen Patienten bei:<br>• Schwerer Toxizität<br>• Zweiter Schlaganfall oder große Hämorrhagie innerhalb des Behandlungzeitraumes<br>• Größeren bedrohlichen Nebenwirkungen, die als Nebenwirkungen von Neupogen® bekannt sind<br>• Allergien gegen Neupogen $^{R}$ oder Escherichia coli Produkte<br>• Leukozyten > 70.000 /µl mit klinisch-neurologischer und/oder systemischer Verschlechterung<br>• Auftreten einer bisher unbekannten KHK<br>• Wunsch des Patienten zum Studienabbruch |

Patienten, bei denen ein vorzeitiges Studienende vorauszusehen ist oder bei denen die Studie abgebrochen wurde, sollen möglichst der Abschlussuntersuchung unterzogen und entsprechend dokumentiert werden.

### 11.2. Für die gesamte Studie

Die gesamte Prüfung kann vom Leiter der klinischen Prüfung unter Abwägung des Nutzen/Risiko-Verhältnisses jederzeit unterbrochen oder beendet werden. Ein Abbruch oder eine Unterbrechung der Gesamtstudie erfolgt ausserdem, wenn das externe Protokoll-Review-Kommitee dies dringend vorschlägt.

## 12. Dokumentation

Alle während der Studie erhobenen relevanten Daten von allen in die Studie eingebrachten Patienten sind vom verantwortlichen Studienarzt oder einer von ihm beauftragten Person in die für diese Studie erstellten Dokumentationsbögen (CRF's) oder andere entsprechende Dokumentationsmittel einzutragen und mit Unterschrift und Datum abzuzeichnen. Die Daten müssen vollständig und plausibel sein. Die Eintragungen müssen eindeutig und leserlich vorgenommen werden.

Korrekturen auf den Originalbögen werden so durchgeführt, dass die ursprüngliche Eintragung sichtbar bleibt. Ergänzungen und Korrekturen werden vom verantwortlichen Arzt oder einer autorisierten Person datiert und signiert. Nicht selbsterklärende Korrekturen sind zu begründen.

# 13. Datenmanagement und statistische Aspekte

## 13.1. Datenerhebung

Die Datenerhebung erfolgt in den dafür vorgesehenen Dokumentationsbögen (CRF's) wie oben definiert. Nach Abschluss der Verlaufsbeobachtung beim jeweiligen Patienten wird der Dokumentationsbogen dem Leiter der klinische Prüfung bzw. seinem Beauftragten zugestellt. Nach Überprüfung und Durchführung erforderlicher Korrekturen und Ergänzungen erfolgt die Datenverarbeitung.

## 13.2. Statistische Methoden

Es sollen Patienten im Rahmen dieses Phase I/II-Studienprotokolls behandelt werden. Die statistische Auswertung erfolgt im Rahmen eines intent-to-treat Designs.

## 13.3. Auswertbarkeit und Auswertung

Patienten/-innen, bei denen grobe Verstöße gegen die Ein- und Ausschlusskriterien (‚non-eligible') vorliegen, werden von der Auswertung ausgeschlossen. Über diese Fälle wird lediglich kasuistisch berichtet.

Alle übrigen Patienten fließen in die Bewertung des primären Zielkriteriums ein (siehe dazu auch Abbruchkriterien). Es werden zwei Auswertungskollektive gebildet:

- Die Intent-to-treat Stichprobe (ITT) umfaßt alle Patienten, bei denen zumindest mit der Therapie begonnen wurde (erste Medikamenteneinnahme).
- Aus der ITT-Stichprobe wird im weiteren die Per-Protokol Stichprobe abgegrenzt: Patienten, die einer nicht protokollgerechten Behandlung unterzogen wurden, werden dabei aus der Endauswertung ausgeschlossen.

Über die Auswertbarkeit von einzelnen Patienten bezüglich Toxizität oder Funktionalität ist nach Vorlage der vollständigen Dokumentation durch den Leiter der klinischen Prüfung, ggfs. das externe Protokoll-Review Komitee (PRK) zu entscheiden.

## 14. Monitoring und Qualitätskontrolle

Der protokollgemäße Ablauf der klinischen Prüfung sowie die Vollständigkeit, Korrektheit und Plausibilität der ausgefüllten Dokumentationsbögen (CRF`s) wird durch den Studienleiter sichergestellt. In regelmäßigen Abständen wird vor allem ein Quelldatenvergleich durchgeführt. Der Studienleiter berichtet dem Protokoll-Review Kommitee (PRK).

## 15. Ethische und regulatorische Belange

### 15.1. Ethikkommission

Über das Prüfvorhaben, insbesondere den Prüfplan, die Patienteneinverständniserklärung und -aufklärung, wird die für den Leiter der Studie zuständige Ethikkommission beraten. Nach Vorlage eines positiven Votums, der Patientenversicherung sowie der Meldung an das *Bpharm*, kann mit der klinischen Prüfung begonnen werden.

### 15.2. Patienteninformation und Datenschutz

Jeder Patient wird über die Modalitäten der Verlaufsbeobachtung entsprechend der beiliegenden Patienteninformation unterrichtet. Die Aufklärung des Patienten erfolgt sowohl schriftlich als auch mündlich durch den behandelnden Arzt. Dem Patienten muss ausreichend Gelegenheit gegeben werden, sich für oder gegen eine Teilnahme an dieser Studie zu entscheiden und hierzu Fragen zu formulieren. Er ist ferner darauf hinzuweisen, dass er jederzeit die Möglichkeit hat, die Teilnahme an der Studie ohne Angabe von Gründen zu beenden und ihm dadurch keine Nachteile entstehen. Die Aufklärung umfaßt folgende Punkte:

1) die Art der Erkrankung
2) die Prognose allgemein
3) die zur Verfügung stehenden Therapiemöglichkeiten
4) Hintergrund und Zielsetzung der Studie
5) Forschungscharakter der Studie
6) Art der Behandlung und Randomisierung
7) Ablauf und Dauer der Studie, Anzahl der Teilnehmer
8) Art und Ziele der Verlaufsbeobachtung (3 Monate)
9) Vorkenntnisse über die eingesetzten Medikamente nach dem Stand der Wissenschaft
10) Verantwortlichkeiten der Studienteilnehmer
11) Mögliche Risiken und Unannehmlichkeiten, sowie die zu erwartenden Wirkungen und Nebenwirkungen der Therapie
12) Mögliche Vorteile der Behandlung
13) Alternative Behandlungsmöglichkeiten
14) Versicherungsschutz
15) Behandlungsmaßnahmen im Falle studienbedingter unerwünschter Ereignisse
16) Finanzielle Belastungen bzw. Zuwendungen bei Studienteilnahme
17) die Wahrung der Vorschriften über die ärztliche Schweigepflicht und den Datenschutz
18) Freiwilligkeit der Studienteilnahme und Widerruf der Einwilligung

19) die Möglichkeit der Prüfung personenbezogener Originaldaten durch die Behörden oder ordnungsgemäß autorisierte Personen
20) Vertraulichkeit und Datenschutz, Speicherung der Daten in anonymer Form
21) Informationspflicht des behandelnden Prüfarztes gegenüber dem Studienteilnehmer über wichtige Ergebnisse aus dem bisherigen Studienverlauf
22) Ansprechpartner für (Rück-) Fragen bzw. in Notfällen
23) Vorhersehbare Gründe für eine vorzeitige Beendigung der Studie bei einem Teilnehmer
24) Verpflichtungen des Patienten (keine anderen Therapien etc.).

Die hier angegeben Punkte sollen dem Patienten in einer für ihn verständlichen Sprache erläutert werden. Der aufklärende Arzt und der Patient datieren und unterzeichnen hierzu eine Einverständniserklärung (s. Anlage). Eine Kopie der Aufklärung und Einverständnis verbleibt beim Patienten, die originalen Einverständniserklärungen sind Bestandteil der Patientenakte und werden mit dieser archiviert.

Die Vorschriften über die ärztliche Schweigepflicht und den Datenschutz werden gewahrt. Sollte die Datenüberprüfung eine direkte Einsichtnahme in die Patientenakte erforderlich machen, ist dies ebenfalls vom Patienten schriftlich zu genehmigen.

### 15.3. Patientenversicherung

Entsprechend der arzneimittelrechtlichen Bestimmungen wurde für alle an der Studie teilnehmenden Patienten eine Versicherung an der Universität Regensburg (s.u. Anlage), wie im Ethikantrag näher definiert, abgeschlossen.

### 15.4. Studienfinanzierung

Bisher noch keine Finanzierung.

### 15.5. Prüfplanergänzungen (Amendments)

Relevante Ergänzungen und Änderungen des Prüfplans nach Erteilung des Ethikvotums müssen schriftlich formuliert (Amendment) und der Ethikkommission zur Kenntnis gegeben werden.

Änderungen, die das Gesundheitsinteresse des Patienten berühren, erfordern ein neues Votum der Ethikkommission und eine erneute schriftliche Einwilligung der Patienten, die es betrifft. Alle Amendments bedürfen der schriftlichen Zustimmung des Studienleiters.

Ergänzungen und Änderungen des Prüfplans nach Studienbeginn werden allen Prüfärzten unmittelbar schriftlich mit dem vereinbarten Termin der Änderung mitgeteilt. Die Kenntnisnahme muss schriftlich bestätigt werden.

## 15.6. Gesetzliche Bestimmungen und Richtlinien

Die klinische Prüfung wird in Übereinstimmung mit den Bestimmungen des deutschen Arzneimittelgesetzes (AMG, in der jeweils gültigen Fassung), der „Deklaration von Helsinki“ sowie den Grundsätzen für Standards der guten klinischen Praxis (ICH-GCP) durchgeführt.

## 15.7. Allgemeine Anzeigepflicht

Entsprechend dem Arzneimittelgesetz wird die klinische Prüfung bei den zuständigen Behörden des Studienleiters sowie ggf. weiterer Studienteilnehmer vorgelegt bzw. gemeldet. Der Studienleiter übernimmt diese Meldung mit Zustimmung der Studienteilnehmer und informiert diese über den Vollzug der Anzeige durch eine Kopie.

## 15.8. Prüfarztinformation

Der Prüfarzt wird durch den Leiter der klinischen Prüfung ständig über relevante, neue Erkenntnisse über das eingesetzte Präparat (G-CSF-Filgrastim) informiert.

## 16. Vereinbarungen und organisatorische Abläufe

Der Leiter der klinischen Prüfung verfügt über eine mindestens zweijährige Erfahrung in der klinischen Prüfung von Arzneimitteln/Medizinprodukten.

### 16.1. Leiter der klinischen Prüfung und Studienzentrale:

**Prof. Dr. med. U. Bogdahn**
Klinik und Poliklinik für Neurologie der Universität Regensburg
im Bezirksklinikum
Universitätsstrasse 84
93053 Regensburg

### 16.2. Studienkoordination, medizinische Dokumentation und Datenmangement:

PD. Dr. med. Jürgen Winkler
Tel: 0941-941-3001
e-mail: juergen.winkler@klinik.uni-regensburg.de

Dr. med. Markus Horn
Tel.: 0941 / 9 41 3330
e-mail: markus.horn@klinik.uni-regensburg.de

Dr. med. Guntram Ickenstein
Tel.: 0941 / 9 41 3012
e-mail: guntram.ickenstein@klinik.uni-regensburg.de

## 17. Literaturverzeichnis


1. Gage FH. Mammalian neural stemm cells. Science 2000; 287: 1433-1438

2. Brazelton TR, Rossi FMV et. al.. From marrow to brain: Expression of neuronal phenotypes in adult mice. Science 2000; 290: 1775-1779

3. Mezey E, Chandross KJ, et. al.. Turning blood into brain: Cells Bearing neuronal antigens generated in vivo from bone marrow. Science 2000; 290: 17791782

4. Anderson DJ, Gage FH, Weismann IL. Can stem cells cross lineage boundaries. Nature Medicine 2001; 7: 393-395

5. Orlic D, Kajstura J, et. al.. Bone marrow cells regenerate infarcted myocardium. Nature 2001; 410: 701-705

6. Kunh HG, Svendsen CN. Origins, functions and potential of adult neural stem cells. BioEssays 1999; 21: 625-630

7. Bjornson CR, Rietze RL, et. al.. Turning brain into blood: A hematopoietic fate adopted by adult neural stem cells in vivo. Science 1999; 283: 534-537

8. van der Kooy D, Weiss S. Why stem cells?. Science 2000; 287: 1439-1441

9. Kondziolka D, Wechsler L, et. al.. Transplantation of cultured human neural cells for patients with stroke. Neurology 2000; 55: 565-569

10. Chen J, Li Y, et. al.. Therapeutic benifit of intravenous andministration of bone marrow stromal cells after cerebral ischemia in rats. Stroke 2001; 1001-1011

11. Zhao L, Duan W, et. al.. Human bone marrow stem cells exhibit neural phenotypes and ameliorate neurological deficits anter grafting into the ischemic brain of rats. Experimental Neurology 2002; 174: 11-20

12. Morshead CM, Benveniste P, et. al.. Hematopoietic competence is a rare proporty of neural stem cells that may depend on genetic and epigenetic alterations. Nature Medicine 2002; 8: 268-273

13. Li Y, Chen J, et. al.. Treatement of stroke in rat with intracarotid administration of marrow stromal cells. Neurology 2001; 56

14. Zivin JA. Cell transplant therapy for stroke. Neurology 2000; 55:467

15. Nakatomi H, Kuriu T, Okabe S, Yamamoto S, Hatano O, Kawahara N, Tamura A, Kirino T, Nakafuku M. Regeneration of hippocampal pyramidal neurons after ischemic brain injury by recruitment of endogenous neural progenitors. Cell in press 2002.

16. Gu W, Brännström T, Wester P. Cortical neurogenesis in adult rats after reversible photothrombotic stroke. J Cerebr Blood Flow Met 2000; 20: 1166 – 1173

17. Magavi S, Leavitt BR, Macklis JD. Induction of neurogenesis in the neocortex of adult mice. Nature 2000; 405: 951 – 955

18. Zhao L, Duan W, Reyes M, Keene C, verfaillie C, Low W. Human bone marrow stem cells exhibit neural phenotypes ans ameliorate neurological deficits after grafting into ischemic brain of rats. Exper Neurology 2002; 174: 11-20

19. Kee N, Preston E, Wojtowicz JM. Enhanced neurogenesis after transient global ischemia in the dentate gyrus of the rat. Exp brain res 2001; 136:313 – 320

20. Iwai M, Sato K, Omori N, Nagano I, Manabe Y, Shoji M, Abe K. Three steps of neural stem cells development in gerbil dentate gyrus after transient ischemia. J Cerebr Blood Flow Met 2001; 22:411-419

21. Albers G et al. Antithrombotic and thrombolytic therapy for ischemic stroke. Chest 1998; 114: 683-698

22. Morstyn G, Dexter TM, Food MA. Filgrastim (r-metHuG-CSF) in Clinical Practice. In Marcel Dekker 1998

23. Cavallaro AM, Lilleby K, Majolino I, Storb R, Appelbaum FR, Rowley SD, Bensinger WI. Three to six year follow-up of normal donors who received recombinant human granulocyte colony-stimulating factor.*Bone Marrow Transplant* 2000; 25(1):85-89

24. Schaeblitz WR, Kollmar R, Schwaninger M, Sommer C, Schoelzke M, Schwab S, Wildemann B. Recombinant granulocyte-colony stimulating factor (rhG-CSF) is neuroprotective following focal transient cerebral ischemia and excitotoxicity*. Society for Neuroscience Abstracts* 2001; 27(2): 2027

25. Ohara N, Okamura S, Kubota A, Shimoda K, Umei T, Itoh S, Kuwahara H, Niho Y. Levels of serum granulocyte colony-stimulating factor in patients with cerebrovascular disease. Life Sci 1993;53 (11):939-943

26. Hierholzer C, Kalff JC, Billiar TR, Bauer A, Tweardy DJ. Activation of STAT proteins following ischemia reperfusion injury demonstrates a distinct IL-6 and G-CSF mediated profile. Transplant Proc. 1998; 30: 2047

27. Tamura M, Hattori K, Nomura H. Induction of neutrophilic granulocytosis in mice by administration of purified human native granulocyte colony stimulating factor (G-CSF). Biochem Biophys Res Commun 1987;142:454 - 460

28. Scheffler B, Horn M, Blumcke I, Laywell ED, Coomes D, Kukevov VG, Steindler DA. Marrow-mindedness: a perspective on neuropoiesis. Trends Neurosci 1999; 22(8): 348 - 357

29. Sohngen D, Wienen S, Siebler M, Boogen C, Scheid C, Schulz A, Kobbe G, Diehl V, Heyll A. Analysis of rhG-CSF-effects on platelets by in vitro bleeding test and transcranial Doppler ultrasound examination. Bone Marrow Transplant 1998; 22: 1087-1090

30. Slevin M, Krupinski J, Slowik A, Kumar P, Szczudlik A, Gaffney J. Serial measurement of vascular endothelial growth factor and transforming growth factor-beta1 in serum of patients with acute ischemic stroke. Stroke 2000; 31: 1863-1870

31. McQualter JL, Darwiche R, et. al.. Granulocyte macrophage colony-stimulating factor: A new putative therapeutic tartget in multiple sclerosis. J. Exp. Med. 2001; 194: 873-881.

32. Guan K, Chang H, et. al.. Embryonic stem cell-derived neurogenesis. Retinoid acid induction and lineage selection of neural cells. Cell Tissue Res 2001; 305 (2): 171-6

33. Benraiss A, Chmielnicki E, et.al.. Adenoviral brain-derived neutrophic factor induces both neostriatal and olfactory neuronal recruitment from endogenous progenitor cells in the adult forebrain. J Neurosci 2001; 21(17): 6718-31

34. Storch A, Paul G, et. al.. Long-term proliferation and dopaminergic diefferentiation of human mesencephalic neural precursor cells. Exp Neurol 2001; 170(2): 317-25

35. Sawamoto K, Nakao N, et. al.. Generation of dopaminergic neurons in the adult brain from mesencephalic precursor cells labeled with a nestin-GFP transgene. J Neurosci 2001; 21(11): 3895-903

36. Fallon J, Reid S, et.al.. In-vivo induction of massive proliferation, directed migration, and differentiation of neural cells in the adult mamalian brain. Proc Natl Acad Sci 2000; 97(26): 14686-91

37. Li Y, Chen J, M. Chopp. Adult bone marrow transplantation after stroke in adult rats. Cell Transplant 2001; 10(1): 31-40

38. Strauer BE, Brehm M, et. al.. Myocardial regeneration after intracoronary transplantation of human autologous stem cells following acute myocardial infarction. Dtsch Med Wochenschrift 2001; 126: 932-938.

39. Coplin WM, Cochran MS, et. al. Stroke after bone marrow transplantation: frequency, etiology and outcome. Brain 2001; 124: 1043-1051.

40. Anderlini P, Przepiorka D, Seong D, Miller P, Sundberg J, Lichtiger B, Norfleet F, Chan KW, Champlin R, Korbling M. Clinical toxicity and laboratory effects of granulocyte colony-stimulating factor (filgrastim) mobilization and blood stem cells apheresis from normal donors, and analysis of charges for the procedures. *Transfusion*. 1996; 36:590-595

41. Anderlini P, Przepiorka D, Lauppe J, Seong D, Giralt S, Champlin R, Korbling M. Collection of peripheral blood stem cells from normal donors 60 years of age or older. *British Journal of Hämatology*. 1997; 485-487.

42. Arbona C, Prosper F, Benet I, Mena F, Solano C, Garcia-Conde J. Comparison between once a day vs twice a day G-CSF for mobilization of peripheral blood progenitor cells (PBPC) in normal donors for allogeneic PBPC transplantation. *Bone Marrow Transplantation*. 1998; 22:39-45.

43. Gonzalez M, Benito A, Diaz MA, Madero L. Peripheral blood progenitor cell (PBPC) collection by large-volume leukapheresis from pediatric donors [letter]. *Bone Marrow Transplantation*. 1999; 23:631-632.

44. Kawano Y, Takaü Y, Watanabe T, Watanabe A, Iwai A, Kuroda Y. Peripheral blood stem cell mobilization with granulocyte colony-stimulating factor and a harvesting procedure in pediatric donors. *Bone Marrow Transplantation*. 1998; 21(S3):S32-S34.

45. Ordemann R, Holig K, Wagner K, Rautenberg U, Bornhauser M, Kroschinsky F, Schafter J, Schuler U, Ehninger G. Acceptance and feasibility of peripheral stem cell mobilisation compared to bone marrow collection from healthy unrelated donors. *Bone Marrow Transplantation*. 1998; 21(S3):S25-S28.

46. Stroncek DF, Clay ME, Smith J, Ilstrup S, Oldham F, McCullough J. Changes in blood counts after the administration of granulocyte colony-stimulating factor and the collection of peripheral blood stem cells from healthy donors. *Transfusion*. 1996; 36:596-600.

47. Stroncek DF, Clay ME, Smith J, Ilstrup S, Oldham F, McCullough J. Treatment of normal individuals with granulocyte colony-stimulating factor: donor experiences and the effects on peripheral blood CD 34+ cell counts and on the collection of peripheral blood stem cells. *Transfusion*. 1996; 36:601-610.

48. Waller CF, Bertz, H, Wenger MK, Fetscher S, Hardung M, Engelhardt M, Behringer D, Lange W, Mertelsmann R, Finke J. Mobilization of peripheral blood progenitor cells for allogeneic transplantation: efficacy and toxicity of a high-dose rhG-CSF regimen. *Bone Marrow Transplantation.* 1996; 18:279-283.

49. Majolino I, Cavallaro AM, Bacigalupo A, Rambaldi A, Falda M, Locatelli F, Faimondi R, Pucci G, Mordini N, Barbui T, Rodeghiero F, Irrera G, Indovina A, Valbonesi M, Bellavita P, Tassi V, Perotti C, Soli M, Bresolin G. Mobilization and collection of PBSC in healthy donors: a retrospective analysis of the Italian Bone Marrow Transplantation Group (GITMO). *Hematologica*. 1997; 82:47-52.

50. Tabilio A, Falzetti F, Giannoni C, Aversa F, Martelli MP, Rossetti M, Caputo P, Chionne F, Gambelunghe C, Martelli MF. Stem cell mobilization in normal donors. *Journal of Hematotherapy*. 1997; 6:277-234.

51. Becker PS, Wagle M, Matous S, Swanson RS, Pihan G, Lowry PA, Stewart FM, Heard SO. Spontaneous splenic rupture following administration of granulocyte colony-stimulating factor (G-CSF): Occurrence in an allogeneic donor of peripheral blood stem cells. *Biology of Blood and Marrow Transplantation*. 1997; 3:45-49.

52. Adkins DR. Anaphylactoid reaction in a normal donor given granulocyte colony-stimulating factor [letter]. *Journal of Clinical Oncology*. Feb 1998; 16(2):812-813.

53. Spitzer T, McAfee S, Poliquin C, Colby C. Acute gouty arthritis following recombinant human granulocyte colony-stimulating factor therapy in an allogeneic blood stem cell donor [letter]. *Bone Marrow Transplantation*. 1998; 21:966-967.

54. Baker D, Durrant S. Fatal stroke following Filgrastim mobilisation of PBPC for allogeneic transplantation. Abstract presented at the *25th Annual Meeting of the European Group for Blood and Marrow Transplantation*. 1999.

55. Vaz CP, Correia O, Henrique R, Campilho F, Campos A, Pimentel P. Recombinant granulocyte colony-stimulating factor (rhG-CSF) associated vasculitis during stem cell mobilization. *Bone Marrow Transplantation*. 1999; 23(S1):abstract 266.

56. Vij R, Adkins DR, Brown RA, Khoury H, DiPersio JF, Goodnough T. Unstable angina in a peripheral blood stem and progenitor cell donor given granulocyte colony-stimulating factor [letter]. *Transfusion*. 1999; 39:542-543.

57. Margolis D, Dincer A, Anderson L, Moraski L, Casper J, Gottschall J. Serious Adverse Events in Parental Donors Undergoing Peripheral Blood Progenitor Cell Mobilization: A Trigger for Changing the Donation Process. *43rd Annual Meeting of American Society of Hematology*, Orlando, Florida, December 7-11, 2001, abstract no. 750

58. Schmid-Schönbein GW, Usami S, Skalak R, Chien S. The interaction of leukocytes and erythrocytes in capillary and postcapillary vessels. Microvasc Res 1980; 19: 45

59. Chien S, Gallik S. Rheology in normal individuals and polycythemia vera. In: Wasserman LR, Berk PD, Berlin N (eds.). Polycythemia vera and the myeloproliferative disorders. 1995: 335

# 18. Anhänge

| | | |
|---|---|---|
| 18.1 | Appendix A: | Patientenaufklärung |
| 18.2 | Appendix B: | Einverständniserklärung |
| 18.3 | Appendix C: | Patientenversicherung |
| 18.4 | Appendix D: | Ethikvotum |
| 18.5 | Appendix E: | Deklaration von Helsinki |
| 18.6 | Appendix F: | Produktinformationen |
| 18.7 | Appendix G: | Datenerfassungsbögen (CRF) |
| 18.8 | Appendix H: | Liste zur Identifikation der Patienten |
| 18.9 | Appendix I: | NIH-Stroke Scale (NIHSS) |
| 18.10 | Appendix J: | Modified Rankin Scale (RS) |
| 18.11 | Appendix K: | Barthel Index (BI) |
| 18.12 | Appendix L: | Functional Independence Measures (FIM) |
| 18.13 | Appendix M: | Testpsychologie |
| 18.14 | Appendix N: | SSQL |
| 18.15 | Appendix O: | Bericht über schwerwiegende unerwünschte Ereignisse (SAE´s) |
| 18.16 | Appendix P: | Bericht über unerwünschte Arzneimittelwirkungen (BfArM) |
| 18.17 | Appendix Q: | Synopsis Study Protokoll |

## Appendix A

### Patientenaufklärung

**zur klinischen Studie**

### Regeneration beim akuten ischämischen Schlaganfall – Programm zur Entwicklung einer Stammzellbasierten Therapie: Eine Phase I/II-Studie – Protokoll-Nr.:

Sehr geehrte Patientin, sehr geehrter Patient,

Sie wurden von Ihrer Ärztin/ Ihrem Arzt gefragt, ob Sie an der oben genannten wissenschaftlichen Studie mit **Filgrastim (Neupogen ®)** teilnehmen möchten. Die Studie wird unter der Leitung von Herrn Professor U. Bogdahn in der Neurologischen Universitätsklinik Regensburg durchgeführt. Es ist wichtig, dass Sie die nachfolgenden allgemeinen Grundsätze, die für alle Studienteilnehmer gelten, sorgfältig lesen und verstehen. Die Teilnahme an dieser wissenschaftlichen Studie ist vollkommen freiwillig. Sie können sich auch gegen eine Teilnahme an dieser Studie entscheiden oder jederzeit aus der Studie ausscheiden, ohne dass dies mit irgendwelchen Nachteilen für Ihre weitere medizinische Behandlung verbunden ist.

**HINTERGRUNDINFORMATIONEN UND ZIEL DER KLINISCHEN STUDIE**
Sie werden gebeten an dieser Studie teilzunehmen, da Ihre Ärzte bei ihnen einen Schlaganfall diagnostiziert haben, der durch ein Blutgerinnsel in einem Ast der sogenannten Arteria cerebri media verursacht wurde. Die Arteria cerebri media ist eines der wichtigsten, das Gehirn mit Blut versorgenden Gefäße, wobei sie sich in ihrem Verlauf wie ein Baum in mehrere Äste aufteilt.

Diese Art von Schlaganfall, bei der ein Teil des Gehirns von der Blutversorgung kurzfristig abgeschnitten wurde, wird als „ischämischer Schlaganfall“ bezeichnet. Das mit Blut nicht versorgte Gehirngewebe wird kurzfristig minderversorgt, was in der Regel eine körperliche Beeinträchtigung (z.B. Lähmung, Sprachstörung) zur Folge hat. Gelingt es nicht, das verschlossene Gefäß innerhalb der ersten Stunden wiederzueröffnen, kann bis heute nur die Rehabilitation (Krankengymnastik, Training, Sprachtherapie) zu einer Besserung der körperlichen Beeinträchtigung führen. Dabei werden Aufgaben des zerstörten Gehirngewebes von anderen Gehirngebieten teilweise übernommen.

Aus experimentellen Daten ist bekannt, dass körpereigene Knochenmarkstammzellen, die im Körper mit dem Blut verteilt werden, sich unter bestimmten Umständen in neurale, also hirnansässige Stammzellen umwandeln können. In Tierversuchen ließ sich zeigen, dass damit die Folgen des Schlaganfalles gemindert werden können. Ein Anstieg der Konzentration an körpereigenen Knochenmarkstammzellen könnte somit letztendlich zu einer Besserung, der durch den „ischämischen Schlaganfall“ verursachten Beeinträchtigung führen.

**Filgrastim (Neupogen ®)** ist ein Medikament, welches das Wachstum der körpereigenen Knochenmarkstammzellen anregt. Es wird seit Jahren mit Erfolg routinemäßig bei Tumor und Leukämiepatienten eingesetzt, um unerwünschte Wirkungen einer Chemotherapie zu mindern sowie bei Knochenmarkspendern.

Zur Anwendung von **Filgrastim (Neupogen ®)** bei Schlaganafällen liegen dagegen keine Erfahrungen vor. In dieser klinischen Studie soll untersucht werden, ob **Filgrastim (Neupogen ®)** eine verträgliche und wirksame Therapie darstellt, die dazu beiträgt, die Regeneration im geschädigten Gehirngewebe und die damit einhergehende körperliche Beeinträchtigung zu bessern. An dieser Studie werden mindestens 15 Patienten teilnehmen. Die Dauer der Studie beträgt drei Monate für den 1. Patienten.

**ABLAUF DER STUDIE**
Wenn Sie sich für eine Teilnahme an dieser Studie entscheiden, ist der weitere Ablauf wie folgt:

Zunächst werden Sie gebeten, diese Patienteninformation mit Einverständniserklärung sorgältig zu lesen und, nachdem alle Ihre Fragen beantwortet wurden, zu unterzeichnen. Bevor Sie die Studienmedikation erhalten werden bei Ihnen einige Untersuchungen dürchgeführt, die größtenteils zur Standardabklärung eines Schlaganfalls gehören. Im einzelnen sind dies: eine Erhebung der Krankengeschichte, eine ausführliche körperliche Untersuchung einschließlich der Bestimmung Ihrer Körpertemperatur, ein Elektrokardiogramm, ein Computertomogramm ihres Kopfes, eine Ultraschalldarstellung Ihrer Gehirngefäße, ein Kernspintomogramm Ihres Kopfes, eine Blutuntersuchung und bei Frauen unter fünfzig Jahren ein Schwangerschaftstest.

Unmittelbar nach Abschluß der oben genannten Untersuchungen erhalten Sie die Studienmedikation Filgrastim (Neupogen ®). Das Medikament wird subkutan (über eine kleine Nadel unter Ihre Haut) verabreicht. Die Studienmedikation wird Ihnen insgesamt fünf mal an fünf aufeinanderfolgenden Tagen gegeben.

Um die Sicherheit und Wirkung der Studienmedikation beurteilen zu können werden Sie weiteren Untersuchungen und Befragungen unterzogen. Hierzu gehören im einzelnen:

- insgesamt 2 weitere Kernspintomogramme Ihres Kopfes (am 7. Tag, nach 3 Monaten)
- tägliche Blutentnahmen (in den ersten 7 Tagen, Tag 14, Tag 90)
- wiederholte Körperliche Untersuchung zur Beurteilung der Symptome Ihres Schlaganfalls (zunächst täglich während der ersten Woche sowie nach 2 Wochen und 3 Monaten)
- insgesamt drei ärztliche Befragungen zu den Auswirkungen des Schlaganfalls auf Ihre Fähigkeit zur Ausübung von Alltagsaktivitäten und Ihrer allgemeinen Leistungsfähigkeit (nach einer bzw. zwei Wochen, sowie nach 3 Monaten)
- einmalige ärztliche Befragung zur Auswirkung des Schlaganfalls auf Ihre Lebensqualität (am Ende des 3. Monats)
- testpsychologische Untersuchung (nach 7 tagen und am Ende des 3. Monats)

## Mögliche Risiken und Beschwerden

Filgrastim (Neupogen ®) wurde bei anderen Erkrankungen als Schlaganfall umfassend geprüft. Neben den Beschwerden bei der Entnahme von Blutproben kann es infolge der Gabe von Filgrastim (Neupogen ®) zu weiteren Nebenwirkungen kommen. Diese können u.a. leichte bis mäßige, gelegentlich schwere Knochen- und Muskelschmerzen, Brennen beim Wasserlassen, gelegentlich vorübergehender Blutdruckabfall, gelegentlich leichter bis mässiger Anstieg der weißen Blutkörperchen, geringgradige Blutarmut, Schwindelgefühl, Müdigkeit, leichte Pulsbeschleunigung, leichtes Fieber, Schüttelfrost,

Schwitzen, Brennen an der Einstichstelle, in einigen Fällen Enzündung der kleinen Hautgefäße, selten eine Milzvergrößerung, Milzruptur, Kopfschmerzen, Durchfall, Nasenbluten, Osteoporose, Gelenkschmerzen, Haarausfall, Hautausschlag, Nierenentzündung und pulmonale Infiltrate mit Atemnotsyndrom (ARDS).

Von den genannten unerwünschten Wirkungen praktisch bedeutsam sind jedoch nur Knochenschmerzen und Fieber (Vorkommen bei etwa 20% der behandelten Patienten). Schließlich sei noch einmal darauf hingewiesen, daß Filgrastim (Neupogen ®) bisher bei anderen Erkrankungen als Schlaganfall eingeordnet und geprüft wurde, deshalb kann die Anwendung dieses Medikaments theoretisch bei Patienten mit einem Schlaganfall mit bisher unbekannten Nebenwirkungen verbunden sein.

**FRAUEN IM GEBÄRFÄHIGEN ALTER**

Filgrastim beeinträchtigt nicht die Fähigkeit, zukünftig Kinder zu bekommen. Schwangere oder stillende Frauen dürfen dennoch nicht an dieser klinischen Studie teilnehmen. Zum Ausschluß einer Schwangerschaft wird daher ggf. ein Schwangerschaftstest durchgeführt.

**MÖGLICHER NUTZEN**

Im Rahmen dieser wissenschaftlichen Studie soll die Verträglichkeit und die Wirksamkeit von Filgrastim bei der Behandlung von Patienten mit akutem ischämischem Schlaganfall geprüft werden. Ein direkter Nutzen aus Ihrer Teilnahme an dieser Studie kann Ihnen nicht zugesichert werden, aus theoretischen Überlegungen und experimentellen Daten ist es jedoch denkbar, dass Filgrastim zu einer Besserung der Schlaganfallsymptome über einen Schutz von Nervenzellen und einer vermehrten Regeneration führen kann. Die aus Ihrer Teilnahme gewonnenen Erkenntnisse können darüber hinaus zukünftigen Schlaganfallpatienten helfen.

**ANDERE BEHANDLUNGSMÖGLICHKEITEN**

Sie erhalten unabhängig von Ihrer Teilnahme an dieser Studie die Standardtherapie bei Schlaganfall. Diese können z.B. Medikamente zur Hemmung der Blutplättchen, Senkung des Blutdrucks und des Cholesterins, sowie Heparin sein. rt-PA kann unter bestimmten Voraussetzungen zur Auflösung des den Schlaganfall verursachenden Blutgerinnsels gegeben werden.

**FREIWILLIGE TEILNAHME UND AUSTRITT AUS DER STUDIE**

Ihre Teilnahme an dieser Studie ist vollkommen freiwillig. Sie können die Teilnahme an dieser Studie ablehnen oder Ihre Teilnahme an dieser Studie jederzeit ohne Angabe von Gründen beenden, ohne dass Ihnen Nachteile für Ihre weitere medizinische Versorgung entstehen oder Rechte verlorengehen.

**VORZEITIGE BEENDIGUNG DER STUDIE**

Die an dieser Studie beteiligten Ärzte können die gesamte Studie oder Ihre Teilnahme an dieser Studie ohne Ihre Zustimmung vorzeitig beenden, wenn dies aus ärztlicher Sicht vertretbar und mit den geltenden Richtlinien zur ordnungsgemäßen klinischen Prüfung (GCP) vereinbar ist. Ein Abbruch der Studie kann aus ärztlicher Sicht erforderlich sein, wenn bei Ihnen unter der Behandlung mit der Studienmedikation ein neuer „ischämischer Schlaganfall", eine schwerwiegende Blutung oder eine schwerwiegende Reaktion aufgetreten ist oder Ihrem Arzt neue Informationen zur Sicherheit oder Wirksamkeit von Filgrastim (Neupogen ®) bekannt geworden sind, die gegen die Fortsetzung der Studie sprechen.

**VERSICHERUNG**

Nach den Bestimmungen des Arzneimittelgesetzes (§ 40 AMG) muss für jeden Teilnehmer an einer klinischen Studie eine Probanden-/Patienten-Versicherung abgeschlossen werden.

Deshalb besteht für Sie ein Versicherungsschutz (ECCLESIA Versicherungsdienst GmbH), mit einer Höchstgrenze von Euro 500 000,-- für den Fall einer Gesundheitsschädigung, einer Körperverletzung oder des Todes, die als Folge der Anwendung von Arzneimitteln oder von Maßnahmen an Ihrem Körper bei der Durchführung der klinischen Studie entstanden sind.

Entsprechend den Versicherungsobliegenheiten ist es sehr wichtig, dass Sie die Anweisungen der an dieser klinischen Studie beteiligten Ärzte genau befolgen und die Arzneimittel und Therapiemaßnahmen ordnungsgemäß anwenden. Während der Dauer der klinischen Studie darf eine andere medizinische Behandlung nur nach vorheriger Absprache mit Ihrem Studienarzt durchgeführt werden. Hiervon sind Notfallsituationen ausgenommen.
Eine Gesundheitsschädigung, die als Folge der klinischen Studie eingetreten sein könnte, ist der Versicherung nach Rücksprache mit Ihrem Arzt unverzüglich anzuzeigen.

**VERTRAULICHKEIT**

Patienten- und Krankheitsdaten aus klinischen Studien werden entsprechend den Bestimmungen des Bundesdatenschutzgesetzes streng vertraulich behandelt und nur ohne Angabe Ihres Namens (d.h. anonym) weitergegeben und ausgewertet. Diese Daten können in einem Computer gespeichert und verarbeitet werden.

**WEITERGEHENDE INFORMATION**

Weitere Informationen sowie Antworten auf Ihre Fragen zu dieser Studie oder zu Ihren Rechten als Studienteilnehmer/-in, erhalten Sie von Ihrem behandelndem Arzt

Dr. ___________________________ unter der Telefonnummer 0941-941-_________

Sie werden über alle relevanten Erkenntnisse, die während des Verlaufs der klinischen Studie gewonnen werden und die Ihre Bereitschaft zur weiteren Teilnahme an dieser Studie beeinflussen könnten, in Kenntnis gesetzt. Ihr Arzt kann Ihren Hausarzt über Ihre Teilnahme an dieser Studie informieren. Während der Teilnahme an dieser Studie dürfen Sie an keiner anderen klinischen Studie teilnehmen.

## Appendix B

### Einwilligungserklärung

**zur klinischen Studie**

### Regeneration beim akuten ischämischen Schlaganfall – Programm zur Entwicklung einer Stammzellbasierten Therapie: Eine Phase I/II-Studie – Protokoll-Nr.:

Ich habe die vorstehenden schriftlichen Patienteninformationen sorgfältig gelesen. Darüber hinaus hat mir ______________________________ (Name des aufklärenden Arztes) den Zweck dieser Studie und die geplanten Untersuchungen sowie den möglichen Nutzten und eventuellen Risiken und Beschwerden erklärt. Es wurden auch andere Behandlungsmöglichkeiten besprochen. Ich hatte ausreichend Gelegenheit, etwaige Bedenken zu besprechen und Fragen zu stellen. Dabei wurden alle meine Fragen beantwortet.

Durch Unterzeichnung dieser Einverständniserklärung stimme ich der Teilnahme an der Studie freiwillig zu. Es ist mir bekannt, dass ich diese Zustimmung ohne Angabe von Gründen jederzeit und ohne Nachteile für meine weitere medizinische Versorgung widerrufen kann.

**Mit den im Rahmen der klinischen Prüfung erfolgten Aufzeichnungen von Krankheits- und Studiendaten sowie mit ihrer anonymisierten Weitergabe zur Überprüfung an den Auftraggeber, an die zuständige in- und ausländische Überwachungsbehörde oder die zuständige Bundesoberbehörde bin ich einverstanden. Soweit der Auftraggeber bzw. die zuständige Behörde Einzelangaben über meine persönlichen oder sachlichen Verhältnisse (personenbezogene Daten) benötigt, gestatte ich die Einsichtnahme durch zur Verschwiegenheit verpflichtete Beauftragte. Die gesetzlichen Datenschutzbestimmungen werden eingehalten.**

Ich habe eine Kopie dieser Patienteninformation mit Einverständniserklärung erhalten.

______________________________ ______________________
(Name des Zeugen (in Druckbuchstaben) Unterschrift des Zeugen

______________________________ ______________________
(Name des Patienten (in Druckbuchstaben) Patienten-Nr.

______________________________ ______________________
Unterschrift des Patienten Datum
(eigenhändig vom Patienten einzutragen)

______________________________
Name des aufklärenden Arztes (in Druckbuchstaben)

______________________________ ______________________
Unterschrift des aufklärenden Arztes Datum

## Appendix C - Patientenversicherung

ECCLESIA

Versicherungsdienst GmbH

ECCLESIA Versicherungsdienst GmbH · 32754 Detmold

**Vorab per Fax: 0941 / 944 – 5632**
Klinikum der Universität Regensburg
Zentrum für Klinische Studien
Frau Homeier
Franz-Josef-Strauß-Allee 11

93053 Regensburg

EINGEGANGEN
10. Okt. 2002
Zentrum für Klinische Studien

| Abteilung: | Bearbeitung: | | | |
|---|---|---|---|---|
| Sach II 2.06 | Alexander Madewitz | Telefon: | 05231/603-377 | Detmold, 08.10.02 |
| | | Telefax: | 05231/603-440 | |
| | | Email: | amadewitz@ecclesia.de | |

**Jahresvertrag zur Probandenversicherung Nr. 93567-08**
Ihre Studienanmeldung vom 04.10.2002
Unsere Akte: 030 33 1505 / 1501

Sehr geehrte Frau Homeier,

Sie erhalten die Versicherungsbestätigung zu der Studie „Regeneration nach akutem ischämischen Schlaganfall (RAIS)" sowie die dem Jahresvertrag zur Probandenversicherung zugrunde liegenden „Allgemeinen Versicherungsbedingungen für klinische Prüfungen von Arzneimitteln".

Versicherungsschutz besteht ab dem 01.11.2002.

Die für die angemeldeten 16 Probanden/Patienten zu zahlende Mindestprämie in Höhe von **707,60 €** inklusive Versicherungssteuer von zur Zeit 16% wird mit der Sammelrechnung zum 01.01. des nächsten Jahres erhoben.

Mit freundlichen Grüßen

ECCLESIA
Versicherungsdienst GmbH

Anlagen

Geschäftsführer:
Tilman Kay, Manfred Klocke,
Norbert Noehrbass
Amtsgericht Detmold, HRB 72
Hausanschrift: Klingenbergstraße 4, 32758 Detmold
Telefon 05231/603-0, Telefax 05231/603-197

Gesellschafter:
Evangelische Kirche in Deutschland, Hannover,
Das Diakonische Werk der Evangelischen Kirche
in Deutschland e.V., Hauptgeschäftsstelle Stuttgart,
Deutscher Caritasverband e.V., Freiburg/Br.

Dresdner Bank Detmold,
Kto.-Nr. 282 574 000, BLZ 480 800 20
Deutsche Bank Detmold,
Kto.-Nr. 4 095 006, BLZ 476 700 23

Mitglied im Verband
Deutscher Versicherungs-Makler e.V.

## Appendix D - Ethikvotum

ZKS Regensburg

Zentrum für Klinische Studien
Klinikum der Universität Regensburg D-93042 Regensburg

UNIVERSITÄT REGENSBURG

KLINIKUM

**Zentrum für Klinische Studien**

**Geschäftsstelle der Ethik-Kommission der Medizinischen Fakultät der Universität Regensburg**

Vorsitzender:
Prof. Dr. med. R.Andreesen

Telefon: +49 - (0)941 - 944-5631
Telefax: +49 - (0)941 - 944-5632
ethikkommission@klinik.uni-regensburg.de

Herrn
Prof. Dr. Bogdahn
Klinik und Poliklinik für Neurologie am Bezirksklinikum

21. Okt. 02

**Nr.02/146**
*Regeneration nach akutem ischaemischen Schlaganfall (RAIS), einarmige, dosis-eskalierte Phase II-Studie zur haematopoetischen Stammzellmobilisation bei akutem ischämischen A. cerebri media Teilinfarkt*

**Der Kommission vorgelegte Unterlagen zur Studie:**
Studienprotokoll, Pat. Info. und Einwilligung, Fach. Info

Sehr geehrter Herr Prof. Dr. Bogdahn,

die Ethik-Kommission der Medizinischen Fakultät der Universität Regensburg hat in ihrer Sitzung am 16.10.02 über das o.g. Vorhaben auf Grundlage der oben angegebenen Unterlagen beraten.

**Nachdem alle Auflagen erfüllt sind, erhalten Sie ein uneingeschränkt positives Votum des Vorhabens.**

1. Unabhängig vom Beratungsergebnis verbleibt die ärztliche und juristische Verantwortung beim Projektleiter und seinen Mitarbeitern
2. Die Auflagen der Deklaration von Helsinki des Weltärztebundes in der Fassung von Somerset West / Südafrika 1996 hinsichtlich der ethischen und rechtlichen Aspekte biomedizinischer Forschung am Menschen sind strikt zu beachten.
3. Bitte beachten Sie, dass alle Änderungen oder Erweiterungen in Protokoll sowie der Patienteninformation und -aufklärung schriftlich der Ethik-Kommission mitgeteilt werden müssen. Die Korrekturen sind in der jeweils überarbeiteten Version deutlich kenntlich zu machen.
4. Die Ethik-Kommission erwartet, dass ihr alle schwerwiegenden oder unerwarteten unerwünschten Ereignisse (u.a. alle Todesfälle), die während der Studie auftreten und die Sicherheit der Studienteilnehmer oder die Durchführung der Studie beeinträchtigen können (§40, Abs. 1, Satz 4AMG), unverzüglich schriftlich mitgeteilt werden. Dieses sollte in Verbindung mit einer Stellungnahme des Leiters der Klinischen Prüfung geschehen, ob aus deren Sicht die Nutzen-Risiko-Relation des Vorhabens verändert ist.
5. Der Ethik-Kommission muss der Abbruch oder Abschluss einer Studie mitgeteilt werden. Eine Publikation bzw. der Abschlussbericht der Studie sollte zum gegebenen Zeitpunkt nachgereicht werden. Bei Langzeitstudien bittet die Kommission 12 Monate nach Studienbeginn um Übersendung eines Zwischenberichtes.

Wir wünschen Ihrem Vorhaben ein gutes Gelingen

Prof. Dr. R. Andreesen

Hausanschrift:
Zentrum für klinische Studien
Klinikum der Universität Regensburg
Franz-Josef-Strauß-Allee 11
D-93053 Regensburg

Bankverbindung
Sparkasse Regensburg
Konto Nr. 780 010 500
BLZ 750 500 00

ethikkommission@klinik.uni-regensburg.de
http://www.zks-regensburg.de

# Appendix E

## Deklaration von Helsinki
(Übersetzung aus dem Englischen)

Empfehlung der Ärzte, die in der biomedizinischen Forschung am Menschen tätig sind.
Beschlossen auf der
18. Generalversammlung des Weltärztebundes in Helsinki im Juni 1964;
Revidiert von der 29. Generalversammlung in Tokio im Oktober 1975;
der 35. Generalversammlung in Venedig im Oktober 1983;
der 41. Generalversammlung in Hongkong im September 1989
und der 48. Generalversammlung in Somerset West, Südafrika im Oktober 1996

### Vorwort

Aufgabe des Arztes ist die Erhaltung der Gesundheit des Menschen. Der Erfüllung dieser Aufgabe dient er mit seinem Wissen und Gewissen.

Die Genfer Deklaration des Weltärztebundes verpflichtet den Arzt mit den Worten: „Die Gesundheit meines Patienten soll mein vornehmstes Anliegen sein“, und der internationale Kodex für ärztliche Ethik legt fest: „Jegliche Handlungen oder Beratung, die geeignet erscheinen, die physische und psychische Widerstandskraft eines Menschen zu schwächen, dürfen nur in seinem Interesse zur Anwendung gelangen“.

Ziel der biomedizinischen Forschung am Menschen muss es ein, diagnostische, therapeutische und prophylaktische Verfahren sowie das Verständnis für die Ätiologie und Pathogenese der Krankheit zu verbessern.

In der medizinischen Praxis sind diagnostische, therapeutische oder prophylaktische Verfahren mit Risiken verbunden; dies gilt um so mehr für die biomedizinische Forschung am Menschen.

Medizinischer Fortschritt beruht auf Forschung, die sich letztlich auch auf Versuche am Menschen stützen muss.

Bei der biomedizinischen Forschung am Menschen muss grundsätzlich unterschieden werden zwischen Versuchen, die im wesentlichen im Interesse des Patienten liegen und solchen, die mit rein wissenschaftlichem Ziel ohne unmittelbaren diagnostischen oder therapeutischen Wert für die Versuchsperson sind.
Besondere Vorsicht muss bei der Durchführung von Versuchen walten, die die Umwelt in Mitleidenschaft ziehen könnten. Auf das Wohl der Versuchstiere muss Rücksicht genommen werden.

Da es notwendig ist, die Ergebnisse von Laborversuchen auch auf den Menschen anzuwenden, um die wissenschaftliche Kenntnis zu fördern und der leidenden Menschheit zu helfen, hat der Weltärztebund die folgende Empfehlung als eine Leitlinie für jeden Arzt erarbeitet, der in der biomedizinischen Forschung am Menschen tätig ist. Sie sollte in der Zukunft überprüft werden.

### I Allgemeine Grundsätze

1. Biomedizinische Forschung am Menschen muss den allgemein anerkannten wissenschaftlichen Grundsätzen entsprechen; sie sollte auf ausreichenden Laboratoriums- und Tierversuchen sowie einer umfassenden Kenntnis der wissenschaftlichen Literatur aufbaün.

2. Die Planung und Durchführung eines jeden Versuchs am Menschen sollte eindeutig in einem Versuchsprotokoll niedergelegt werden: dieses sollte einem besonders berufenen unabhängigen Ausschuss zur Beratung, Stellungnahme und Orientierung zugeleitet werden. Dieser Ausschuss sollte unabhängig von Prüfarzt und Sponsor und in Übereinstimmung mit den Gesetzen und Bestimmungen des Landes sein, in dem der Versuch stattfindet.

3. Biomedizinische Forschung am Menschen sollte nur von wissenschaftlich qualifizierten Personen und unter Aufsicht eines klinisch erfahrenen Arztes durchgeführt werden. Die Verantwortung für die Versuchsperson trägt stets ein Arzt und nie die Versuchsperson selbst, auch dann nicht, wenn sie ihr Einverständnis gegeben hat.

4. Biomedizinische Forschung am Menschen ist nur zulässig, wenn die Bedeutung des Versuchsziels in einem angemessenen Verhältnis zum Risiko für die Versuchsperson steht.

5. Jedem biomedizinischen Forschungsvorhaben am Menschen sollte eine sorgfältige Abschätzung der voraussehbaren Risiken im Vergleich zu dem voraussichtlichen Nutzen für die Versuchsperson oder andere vorausgehen. Die Sorge um die Belange der Versuchsperson muss stets ausschlaggebend sein im Vergleich zu den Interessen der Wissenschaft und der Gesellschaft.

6. Das Recht der Versuchsperson auf Wahrung ihrer Unversehrtheit muss stets geachtet werden. Es sollte alles getan werden, um die Privatsphäre der Versuchsperson zu wahren; die Wirkung auf die körperliche und geistige Unversehrtheit sowie die Persönlichkeit der Versuchsperson sollte so gering wie möglich gehalten werden.

7. Der Arzt sollte es unterlassen, bei Versuchen am Menschen tätig zu werden, wenn er nicht überzeugt ist, dass das mit dem Versuch verbundene Wagnis für vorhersagbar gehalten wird. Der Arzt sollte jeden Versuch abbrechen, sobald sich herausstellt, dass das Wagnis den möglichen Nutzen übersteigt.

8. Der Arzt ist bei der Veröffentlichung der Versuchsergebnisse verpflichtet, die Befunde genau wiederzugeben. Berichte über Versuche, die nicht in Übereinstimmung mit den in dieser Deklaration niedergelegten Grundsätzen durchgeführt wurden, sollten nicht zur Veröffentlichung angenommen werden.

9. Bei jedem Versuch am Menschen muss jede Versuchsperson ausreichend über Absicht, Durchführung, erwarteten Nutzen und Risiken des Versuches sowie über möglicherweise damit verbundene Störungen des Wohlbefindens unterrichtet werden. Die Versuchsperson sollte darauf hingewiesen werden, dass es ihr freisteht, die Teilnahme am Versuch zu verweigern und dass sie jederzeit einmal gegebene Zustimmung widerrufen kann. Nach dieser Aufklärung sollte der Arzt die freiwillige Zustimmung der Versuchsperson einholen; die Erklärung sollte vorzugsweise schriftlich abgegeben werden.

10. Ist die Versuchsperson vom Arzt abhängig oder erfolgte die Zustimmung zu einem Versuch möglicherweise unter Druck, so soll der Arzt beim Einholen der Einwilligung nach Aufklärung besondere Vorsicht walten lassen. In einem solchen Fall sollte die Einwilligung durch einen Arzt eingeholt werden, der mit dem Versuch nicht befasst ist und der außerhalb eines etwaigen Abhängigkeitsverhältnisses steht.

11. Ist die Versuchsperson nicht voll geschäftsfähig, sollte die Einwilligung nach Aufklärung vom gesetzlichen Vertreter entsprechend nationalem Recht eingeholt werden. Die Einwilligung des mit der Verantwortung betrauten Verwandten ersetzt die der Versuchsperson, wenn diese infolge körperlicher oder geistiger Behinderung nicht wirksam zustimmen kann oder minderjährig ist. Wenn das minderjährige Kind fähig ist, seine Zustimmung zu erteilen, so muss neben der Zustimmung des Personensorgeberechtigten auch die Zustimmung des Minderjährigen eingeholt werden.

12. Das Versuchsprotokoll sollte stets die ethischen Überlegungen im Zusammenhang mit der Durchführung des Versuchs darlegen und aufzeigen, dass die Grundsätze dieser Deklaration enthalten sind.

## II. Medizinische Forschung in Verbindung mit ärztlicher Versorgung (Klinische Versuche)

1. Bei der Behandlung eines Kranken muss der Arzt die Freiheit haben, neue diagnostische und therapeutische Maßnahmen anzuwenden, wenn sie nach seinem Urteil die Hoffnung bieten, das Leben des Patienten zu retten, seine Gesundheit wiederherzustellen oder seine Leiden zu lindern.

2. Die mit der Anwendung eines neuen Verfahrens verbundenen möglichen Vorteile, Risiken und Störungen des Befindens sollten gegen die Vorzüge der bisher bestehenden diagnostischen und therapeutischen Methoden abgewogen werden.

3. Bei jedem medizinischen Versuch sollten alle Patienten - einschließlich derer einer eventüll vorhandenen Kontrollgruppe - die beste erprobte diagnostische und therapeutische Behandlung erhalten. Dies schließt den Einsatz von Placebo, in Studien für die keine erprobten Diagnostika oder Therapeutika zur Verfügung stehen, nicht aus.

4. Die Weigerung eines Patienten, an einem Versuch teilzunehmen, darf niemals die Beziehung zwischen Arzt und Patient beeinträchtigen.

5. Wenn der Arzt es für unentbehrlich hält, auf die Einwilligung nach Aufklärung zu verzichten, sollten die besonderen Gründe für dieses Vorgehen in dem für den unabhängigen Ausschuss bestimmten Versuchsprotokoll niedergelegt werden.

6. Der Arzt kann medizinische Forschung mit dem Ziel der Gewinnung neuer wissenschaftlicher Erkenntnisse mit der ärztlichen Betreuung nur soweit verbinden, als diese medizinische Forschung durch ihren möglichen diagnostischen oder therapeutischen Wert für den Patienten gerechtfertigt ist.

## III. Nicht-therapeutische biomedizinische Forschung am Menschen

1. In der rein wissenschaftlichen Anwendung der medizinischen Forschung am Menschen ist es die Pflicht des Arztes, das Leben und die Gesundheit der Person zu beschützen, an welcher biomedizinische Forschung durchgeführt wird.

2. Die Versuchspersonen sollten Freiwillige sein, entweder gesunde Personen oder Patienten, für die die Versuchsabsicht nicht mit ihrer Krankheit in Zusammenhang steht.

3. Der ärztliche Forscher oder das Forschungsteam sollten den Versuch abbrechen, wenn dies nach seinem oder ihrem Urteil im Falle der Fortführung dem Menschen schaden könnte.

Bei Versuchen am Menschen sollte das Interesse der Wissenschaft und der Gesellschaft niemals Vorrang vor den Erwägungen haben, die das Wohlbefinden der Versuchsperson betreffen.

## Appendix F - Fachinformationen zu G-CSF-Filgrastim (Neupogen®)

## Appendix G - Datenerfassungsbögen CRF (computergestützte Datenerfassung)

## Appendix H - Liste zur Identifikation der Patienten

| Nr. | Code | Familienname | Vorname | Geburtsdatum | Einschlussdatum |
|---|---|---|---|---|---|
| 1 | □□□ □□□□□□ | | | | |
| 2 | □□□ □□□□□□ | | | | |
| 3 | □□□ □□□□□□ | | | | |
| 4 | □□□ □□□□□□ | | | | |
| 5 | □□□ □□□□□□ | | | | |
| 6 | □□□ □□□□□□ | | | | |
| 7 | □□□ □□□□□□ | | | | |
| 8 | □□□ □□□□□□ | | | | |
| 9 | □□□ □□□□□□ | | | | |
| 10 | □□□ □□□□□□ | | | | |
| 11 | □□□ □□□□□□ | | | | |
| 12 | □□□ □□□□□□ | | | | |
| 13 | □□□ □□□□□□ | | | | |
| 14 | □□□ □□□□□□ | | | | |
| 15 | □□□ □□□□□□ | | | | |
| 16 | □□□ □□□□□□ | | | | |
| | | | | | |
| | | | | | |
| | | | | | |
| | | | | | |

## Appendix I - NIHSS

| | | |
|---|---|---|
| **1a Bewußtsein** | wach<br>somnolent<br>soporös<br>komatös, Streck-/Beugesynergismen, keine mot. Antwort | 0<br>1<br>2<br>3 |
| **1b Orientierung** | Monat, Alter bei erstem Versuch richtig<br>eins richtig bzw. intubiert, schwer dysarthrisch oder Sprachbarriere<br>keins richtig oder aphasisch oder komatös | 0<br>1<br><br>2 |
| **1c Kommando befolgen** | Befolgt beide Aufforderungen<br>Befolgt eine Aufforderung<br>Befolgt keine Aufforderung oder komatös | 0<br>1<br>2 |
| **2 Blick** | Normal<br>Isolierte periphere Parese (N. III, IV, VI) oder überwindbare Blickdev.<br>Fixierte Blickdeviation | 0<br>1<br><br>2 |
| **3 Gesichtsfeld** | Normal<br>Quadrantenanopsie oder Auslöschung<br>Komplette Hemianopsie<br>Blindheit | 0<br>1<br>2<br>3 |
| **4 Mimische Muskulatur** | Normal<br>Geringe zentrale Parese, verstrichene Nasolabial-falte<br>Deutliche zentrale Parese oder Paralyse<br>Beidseitige oder periphere Parese oder Koma | 0<br>1<br><br>2<br>3 |
| **5a Armvorhalte-Versuch <u>links</u>** | 10 sec normal gehalten<br>langsames, unvollständiges Absinken<br>schnelles, vollständiges Absinken<br>Herabfallen<br>Keine Bewegung oder Koma | 0<br>1<br>2<br>3<br>4 |
| **5b Armvorhalte-Versuch <u>rechts</u>** | 10 sec normal gehalten<br>langsames, unvollständiges Absinken<br>schnelles, vollständiges Absinken<br>Herabfallen<br>Keine Bewegung oder Koma | 0<br>1<br>2<br>3<br>4 |
| **6a Beinvorhalte-Versuch <u>links</u>** | 10 sec normal gehalten<br>langsames, unvollständiges Absinken<br>schnelles, vollständiges Absinken<br>Herabfallen<br>Keine Bewegung oder Koma | 0<br>1<br>2<br>3<br>4 |
| **6b Beinvorhalte-Versuch <u>rechts</u>** | 10 sec normal gehalten<br>langsames, unvollständiges Absinken<br>schnelles, vollständiges Absinken<br>Herabfallen<br>Keine Bewegung oder Koma | 0<br>1<br>2<br>3<br>4 |
| **7 Extremitäten-ataxie** | Keine Ataxie oder<br>Pat. versteht Übung nicht oder Koma | 0 |

| | | |
|---|---|---|
| | 1 Gliedmaße ataktisch<br>2 Gliedmaßen ataktisch | 1<br>2 |
| **8 Sensibilität** | Normal<br>Hypästhesie<br>Anästhesie oder Koma | 0<br>1<br>2 |
| **9 Sprache** | Normal<br>Wortfindungsstörungen, leichte Aphasie<br>Deutlich gestörte Konverstation<br>Globale Aphasie, Patient stumm oder komatös | 0<br>1<br>2<br>3 |
| **10 Dysarthrie** | Keine Dysarthrie<br>Dysarthrisch, gut verständlich<br>Dysarthrisch, kaum verständlich oder Pat. antwortet nicht oder Koma | 0<br>1<br>2 |
| **11 Auslöschung** | Nicht vorhanden<br>Auslöschung einer Sinnesmodalität oder andere Zeichen von Neglect<br>Auslöschung mehr als einer Sinnesmodalität oder Koma | 0<br>1<br>2 |

**Appendix J**

# Modified Rankin Scale

| | |
|---|---|
| **0** | No symptoms at all |
| **1** | No signifikanat disability despite Symptoms: able to carry out all usual duties and acitivities |
| **2** | Unable to carry out all previous activities, but able to look after own affairs without assistance |
| **3** | Requiring some help, but able to walk without assistance |
| **4** | Unable to walk without assistance, and unable to attend to own bodily needs with-out assistance |
| **5** | Bedridden, incontinent and requiring constant nursing and attention |

**Appendix K**

# Barthel Index

| | | |
|---|---|---|
| **Essen** | 10 unabhängig<br>5 Hilfe beim Schneiden<br>0 mehr Hilfe | |
| **Transfer Rollstuhl-Bett** | 15 unabhängig<br>10 wenig Hilfe<br>5 alleine Sitzen<br>0 mehr Hilfe | |
| **Körperpflege** | 5 unabhängig<br>0 braucht Hilfe | |
| **Toilettenbenutzung** | 10 unabhängig<br>5 braucht Hilfe<br>0 abhängig | |
| **Badbenutzung** | 5 unabhängig<br>0 abhängig | |
| **Gehen 50m eben** | 15 unabhängig<br>10 mit Hilfe<br>5 Rollstuhl 50m<br>0 nicht möglich | |
| **Treppensteigen** | 10 unabhängig<br>5 mit Hilfe<br>0 nicht möglich | |
| **Anziehen (incl. Schu-he)** | 10 unabhängig<br>5 mit Hilfe<br>0 abhängig | |
| **Darmkontinenz** | 10 kontinent<br>5 gelegentlich inkontinent<br>0 inkontinent | |
| **Blasenkontinenz** | 10 kontinent<br>5 gelegentlich inkontinent<br>0 inkontinent | |
| | Summe | |

## Appendix L

# FIM™ instrument

AAP NEWSLETTER - FALL 1998 - 29

**Functional Independence Measure**

| LEVELS | | |
|---|---|---|
| | 7 Complete Independence (Timely, Safely)<br>6 Modified Independence (Device) | **NO HELPER** |
| | **Modified Dependence**<br>5 Supervision (Subject = 100%+)<br>4 Minimal Assist (Subject = 75%+)<br>3 Moderate Assist (Subject = 50%+)<br><br>**Complete Dependence**<br>2 Maximal Assist (Subject =25%+)<br>1 Total Assist (Subject = less than 25%) | **HELPER** |

| | **ADMISSION** | **DISCHARGE** | **FOLLOW-UP** |
|---|---|---|---|
| **Self-Care** | | | |
| A. Eating | | | |
| B Grooming | | | |
| C. Bathing | | | |
| D. Dressing - Upper Body | | | |
| E. Dressing - Lower Body | | | |
| F. Toileting | | | |
| **Sphincter Control** | | | |
| G. Bladder Management | | | |
| H. Bowel Management | | | |
| **Transfers** | | | |
| I. Bed, Chair, Wheelchair | | | |
| J. Toilet | | | |
| K. Tub, Shower | | | |
| **Locomotion** | | | |
| L. Walk/Wheelchair | W Walk / C Wheelchair / B Both | W Walk / C Wheelchair / B Both | W Walk / C Wheelchair / B Both |
| M. Stairs | | | |
| ***Motor Subtotal Score*** | | | |
| **Communication** | | | |
| N. Comprehension | A Auditory / V Visual / B Both | A Auditory / V Visual / B Both | A Auditory / V Visual / B Both |
| O. Expression | V Vocal / N Nonvocal / B Both | V Vocal / N Nonvocal / B Both | V Vocal / N Nonvocal / B Both |
| **Social Cognition** | | | |
| P. Social Interaction | | | |
| Q. Problem Solving | | | |
| R. Memory | | | |
| ***Cognitive Subtotal Score*** | | | |
| ***TOTAL FIM Score*** | | | |

**NOTE:** Leave no blanks; enter 1 if patient not testable due to risk

Copyright © 1993 Uniform Data System for Medical Rehabilitation, a division of U B Foundation Activities, Inc.

Reprinted with permission of Uniform Data System for Medical Rehabilitation, a division of U B Foundation Activities, Inc.
Guide for the Uniform Data Set for Medical Rehabilitation (including the FIM™ instrument), Version 5.0 Buffalo, NY 14214: State University of New York at Buffalo; 1996.

## Appendix M - Testpsychologie

### 1. LOGICAL MEMORY

Den Patienten werden zwei kurze Geschichten (Geschichte A und Geschichte B) mit jeweils 25 Items langsam vorgelesen. Sie sollen sich den Wortlaut merken und ihn möglichst genau zweimal (sofort und nach 30 Minuten) nacherzählen. Dabei sollen so viele Items wie möglich korrekt wiedergegeben werden.

### 2. REY COMPLEX FIGURE

**Die Patienten sollen eine ihnen vorgelegte Zeichnung möglichst genau unmittelbar und 30 Minuten verzögert aus dem Gedächtnis zeichnen.**

### 3. CONTROLLED ORAL WORD ASSOCIATION (COW)

Die Patienten sollen innerhalb von 1 Minute jeweils so viele Wörter wie möglich nennen, die mit dem Buchstaben B, F und L beginnen. Dabei dürfen keine Namen, Eigennamen und Namen von Städten genannt werden.

### 4. VERBAL FLUENCY

Die Patienten sollen innerhalb von 1 Minute jeweils möglichst viele Tiernamen und Supermarktartikel nennen.

### 5. ZAHLENSPANNE

Die Patienten sollen die Ihnen vorgelesenen Zahlen im ersten Teil des Tests vorwärts, im zweiten Teil des Tests rückwärts wiederholen, wobei die Zahlenspanne immer länger wird.

### 6. ZAHLEN-SYMBOLTEST

Die Patienten sollen innerhalb von 90 Sekunden möglichst vielen Zahlen festgelegte Symbole zuordnen.

**Appendix N**

# SSQL

Name: Geburtsdatum: Datum:

1. Ich fühle mich häufig müde.
1 volle Zustimmung
2 eher Zustimmung
3 weder Zustimmung noch Ablehnung
4 eher Ablehnung
5 volle Ablehnung

2. Ich nehme an Freizeitaktivitäten der Familie nicht teil.
1 volle Zustimmung
2 eher Zustimmung
3 weder Zustimmung noch Ablehnung
4 eher Ablehnung
5 volle Ablehnung

3. Haben Sie Probleme beim Sprechen wie z.B. stottern oder eine undeutliche Aussprache?
1 gar nicht möglich
2 nur unter großen Schwierigkeiten
3 schwierig
4 leichte Schwierigkeiten
5 keine Schwierigkeiten

4. Haben Sie Probleme bei Gehen?
1 gar nicht möglich
2 nur unter großen Schwierigkeiten
3 schwierig
4 leichte Schwierigkeiten
5 keine Schwierigkeiten

5. Ich bin über meine Zukunft entmutigt.
1 volle Zustimmung
2 eher Zustimmung
3 weder Zustimmung noch Ablehnung
4 eher Ablehnung
5 volle Ablehnung

6. Ich bin leicht irritierbar.
1 volle Zustimmung
2 eher Zustimmung
3 weder Zustimmung noch Ablehnung
4 eher Ablehnung
5 volle Ablehnung

7. Brauchen Sie Hilfe beim Zubereiten von Speisen?
1 Komplett auf Hilfe angewiesen
2 Viel Hilfe nötig
3 Hilfe nötig
4 Etwas Hilfe nötig
5 Keine Hilfe nötig

8. Ich gehe nicht so oft aus, wie ich möchte.
1 volle Zustimmung
2 eher Zustimmung
3 weder Zustimmung noch Ablehnung
4 eher Ablehnung
5 volle Ablehnung

9. Ich kann mich schlecht konzentrieren.
1 volle Zustimmung
2 eher Zustimmung
3 weder Zustimmung noch Ablehnung
4 eher Ablehnung
5 volle Ablehnung

10. Haben Sie Probleme beim Schreiben mit der Hand oder mit einer Tastatur ?
1 gar nicht möglich
2 nur unter großen Schwierigkeiten
3 schwierig
4 leichte Schwierigkeiten
5 keine Schwierigkeiten

11. Haben Sie ein Problem, das Bild eines Fernsehgeräts so gut zu sehen, dass Sie eine Fernsehsendung genießen können?
1 gar nicht möglich
2 nur unter großen Schwierigkeiten
3 schwierig
4 leichte Schwierigkeiten
5 keine Schwierigkeiten

12. Haben Sie Probleme, die regelmäßige Arbeit um das Haus herum zu erledigen?
1 gar nicht möglich
2 nur unter großen Schwierigkeiten
3 schwierig
4 leichte Schwierigkeiten
5 keine Schwierigkeiten

13. Ich muss oft während des Tages eine Erholungspause machen.
1 volle Zustimmung
2 eher Zustimmung
3 weder Zustimmung noch Ablehnung
4 eher Ablehnung
5 volle Ablehnung

14. Ich empfinde mich als eine Belastung für meine Familie.
1 volle Zustimmung
2 eher Zustimmung
3 weder Zustimmung noch Ablehnung
4 eher Ablehnung
5 volle Ablehnung

15. Ist es ein Problem für Sie, so deutlich zu sprechen, dass man Sie am Telefon verstehen kann?
1 gar nicht möglich
2 nur unter großen Schwierigkeiten
3 schwierig
4 leichte Schwierigkeiten
5 keine Schwierigkeiten

16. Verlieren Sie das Gleichgewicht, wenn Sie sich nach vorn beugen oder nach etwas greifen ?
1 gar nicht möglich
2 nur unter großen Schwierigkeiten
3 schwierig
4 leichte Schwierigkeiten
5 keine Schwierigkeiten

17. Ich bin an anderen Leuten oder an Aktivitäten nicht interessiert.
1 volle Zustimmung
2 eher Zustimmung
3 weder Zustimmung noch Ablehnung
4 eher Ablehnung
5 volle Ablehnung

18. Ich habe keine Geduld mit anderen.
1 volle Zustimmung
2 eher Zustimmung
3 weder Zustimmung noch Ablehnung
4 eher Ablehnung
5 volle Ablehnung

19. Brauchen Sie Hilfe beim Essen, z.B. beim Schneiden oder Portionieren?
1 Komplett auf Hilfe angewiesen
2 Viel Hilfe nötig
3 Hilfe nötig
4 Etwas Hilfe nötig
5 Keine Hilfe nötig

20. Ich übe Hobbies und andere Freizeitaktivitäten nicht mehr so lange aus, wie ich möchte.
1 volle Zustimmung
2 eher Zustimmung
3 weder Zustimmung noch Ablehnung
4 eher Ablehnung
5 volle Ablehnung

21. Ich habe Schwierigkeiten, mich an Dinge zu erinnern.
1 volle Zustimmung
2 eher Zustimmung
3 weder Zustimmung noch Ablehnung
4 eher Ablehnung
5 volle Ablehnung

22. Haben Sie Probleme beim Anziehen von Socken?
1 gar nicht möglich
2 nur unter großen Schwierigkeiten
3 schwierig
4 leichte Schwierigkeiten
5 keine Schwierigkeiten

23. Haben Sie Probleme, Dinge zu greifen, weil Sie diese Dinge nicht richtig sehen können?
1 gar nicht möglich
2 nur unter großen Schwierigkeiten
3 schwierig
4 leichte Schwierigkeiten
5 keine Schwierigkeiten

24. Ist es für Sie ein Problem, Arbeiten zu Ende zu führen, die Sie begonnen haben?
1 gar nicht möglich
2 nur unter großen Schwierigkeiten
3 schwierig
4 leichte Schwierigkeiten
5 keine Schwierigkeiten

25. Ich bin oft zu erschöpft, um zu tun, was ich wollte.
1 volle Zustimmung
2 eher Zustimmung
3 weder Zustimmung noch Ablehnung
4 eher Ablehnung
5 volle Ablehnung

26. Meine körperliche Leistungsfähigkeit beeinträchtigt mein Familienleben.
1 volle Zustimmung
2 eher Zustimmung
3 weder Zustimmung noch Ablehnung
4 eher Ablehnung
5 volle Ablehnung

27. Haben Gesprächspartner manchmal Probleme, zu verstehen, was Sie sagen?
1 gar nicht möglich
2 nur unter großen Schwierigkeiten
3 schwierig
4 leichte Schwierigkeiten
5 keine Schwierigkeiten

28. Haben Sie Probleme beim Treppensteigen?
1 gar nicht möglich
2 nur unter großen Schwierigkeiten
3 schwierig
4 leichte Schwierigkeiten
5 keine Schwierigkeiten

29. Ich fühle mich isoliert von anderen Leuten.
1 volle Zustimmung
2 eher Zustimmung
3 weder Zustimmung noch Ablehnung
4 eher Ablehnung
5 volle Ablehnung

30. Meine Persönlichkeit hat sich verändert.
1 volle Zustimmung
2 eher Zustimmung
3 weder Zustimmung noch Ablehnung
4 eher Ablehnung
5 volle Ablehnung

31. Brauchen Sie Hilfe beim Ankleiden wie z.B. beim Zuknöpfen oder beim Knoten von Schnürsenkeln?
1 Komplett auf Hilfe angewiesen
2 Viel Hilfe nötig
3 Hilfe nötig
4 Etwas Hilfe nötig
5 Keine Hilfe nötig

32. Ich treffe nicht mehr so viele Freunde, wie ich möchte.
1 volle Zustimmung
2 eher Zustimmung
3 weder Zustimmung noch Ablehnung
4 eher Ablehnung
5 volle Ablehnung

33. Ich muss mir oft Notizen machen, damit ich nichts vergesse.
1 volle Zustimmung
2 eher Zustimmung
3 weder Zustimmung noch Ablehnung
4 eher Ablehnung
5 volle Ablehnung

34. Haben Sie Probleme beim Zuknöpfen?
1 gar nicht möglich
2 nur unter großen Schwierigkeiten
3 schwierig
4 leichte Schwierigkeiten
5 keine Schwierigkeiten

35. Haben Sie Probleme, Dinge am Rande des Gesichtsfeldes zu sehen?
1 gar nicht möglich
2 nur unter großen Schwierigkeiten
3 schwierig
4 leichte Schwierigkeiten
5 keine Schwierigkeiten

36. Ist es für Sie schwierig, die gleichen Arbeiten auszuführen, die Sie früher machen konnten?
1 gar nicht möglich
2 nur unter großen Schwierigkeiten
3 schwierig
4 leichte Schwierigkeiten
5 keine Schwierigkeiten

37. Ist es für Sie ein Problem, im Gespräch das passende Wort zu finden?
1 gar nicht möglich
2 nur unter großen Schwierigkeiten
3 schwierig
4 leichte Schwierigkeiten
5 keine Schwierigkeiten

38. Müssen Sie sich öfter ausruhen, als Sie wollen beim Laufen oder Rollstuhlfahren?
1 gar nicht möglich
2 nur unter großen Schwierigkeiten
3 schwierig
4 leichte Schwierigkeiten
5 keine Schwierigkeiten

39. Ich habe kaum Selbstvertraün.
1 volle Zustimmung
2 eher Zustimmung
3 weder Zustimmung noch Ablehnung
4 eher Ablehnung
5 volle Ablehnung

40. Brauchen Sie Hilfe beim Baden oder Duschen?
1 Komplett auf Hilfe angewiesen
2 Viel Hilfe nötig
3 Hilfe nötig
4 Etwas Hilfe nötig
5 Keine Hilfe nötig

41. Ich habe weniger Sex, als mir wünsche.
1 volle Zustimmung
2 eher Zustimmung
3 weder Zustimmung noch Ablehnung
4 eher Ablehnung
5 volle Ablehnung

42. Haben Sie Probleme beim Öffnen und Schließen eines Reißverschlusses?

1 gar nicht möglich
2 nur unter großen Schwierigkeiten
3 schwierig
4 leichte Schwierigkeiten
5 keine Schwierigkeiten

43. Müssen Sie sich wiederholen, damit andere Sie verstehen?
1 gar nicht möglich
2 nur unter großen Schwierigkeiten
3 schwierig
4 leichte Schwierigkeiten
5 keine Schwierigkeiten

44. Haben Sie Probleme beim Stehen?
1 gar nicht möglich
2 nur unter großen Schwierigkeiten
3 schwierig
4 leichte Schwierigkeiten
5 keine Schwierigkeiten

45. Ich habe keinen großen Appetit.
1 volle Zustimmung
2 eher Zustimmung
3 weder Zustimmung noch Ablehnung
4 eher Ablehnung
5 volle Ablehnung

46. Brauche Sie Hilfe, wenn Sie zur Toilette gehen?
1 Komplett auf Hilfe angewiesen
2 Viel Hilfe nötig
3 Hilfe nötig
4 Etwas Hilfe nötig
5 Keine Hilfe nötig

47. Meine körperliche Leistungsfähigkeit beeinträchtigt meine sozialen Aktivitäten.
1 volle Zustimmung
2 eher Zustimmung
3 weder Zustimmung noch Ablehnung
4 eher Ablehnung
5 volle Ablehnung

48. Haben Sie Probleme beim Aufdrehen von Flaschendeckeln?
1 gar nicht möglich
2 nur unter großen Schwierigkeiten
3 schwierig
4 leichte Schwierigkeiten
5 keine Schwierigkeiten

49. Haben Sie Probleme beim Aufstehen aus einem Stuhl?

1 gar nicht möglich
2 nur unter großen Schwierigkeiten
3 schwierig
4 leichte Schwierigkeiten
5 keine Schwierigkeiten

## Appendix O

| **Bericht über schwerwiegende unerwünschte Ereignisse (SAE)** | **Meldung an**<br><br>Herrn Prof. Dr. Bogdahn |
|---|---|
| (Bitte ankreuzen)<br>O Stationäre Behandlung oder Verlängerung einer stationären Behandlung<br>O lebensbedrohlich<br>O...letal<br>O...Behinderung oder Mißbildung | Klinikum der Universität Regensburg<br>Klinik und Poliklinik für Neurologie<br><br>Tel. 0941-941-3215<br>Fax 0941-941-3205 |

### I. PATIENTENDATEN

| Initialen | Geburtsdatum | Geschlecht | | Pat. Nr.: |
|---|---|---|---|---|
| ___ ___<br>Vor- Nachname | ___ ___ ___ ___ ___ ___<br>Tag Monat Jahr | ☐ männlich<br>☐ weiblich | Größe __ __ __ cm<br>Gewicht __ __ __ kg | ☐☐☐ ☐☐☐☐☐☐☐ |

Name der Studie/AWB:

### II. UNERWÜNSCHTES EREIGNIS

**SÄ** (Symptome, klinisches Bild, Lokalisation, Labor- und Meßwerte, Vorwerte, Diagnostik)

**Auftreten des SÄ (Datum):** ____.____.____

**lebensbedrohlich:** O ja = nein
**Daür, Therapie und Verlauf des SÄ**

**Krankenhausaufenthalt erforderlich oder verlängert:** O ja O nein

**Ausgang der SÄ:**
O wiederhergestellt O noch nicht wiederhergestellt O bleibende Schäden O Exitus, Datum: ____.____.____ O nicht bekannt

### III. VERDÄCHTIGE PRÄPARATE

| Verdächtige Präparate (incl. Oberbegriff) | Tagesdosis | Applika-tionsart und -ort | Therapiedaten (von/bis) | Indikation |
|---|---|---|---|---|
| 1. | | | | |
| 2. | | | | |
| 3. | | | | |

**Vermuteter Zusammenhang mit Arzneimittel Nr.:** 1 2 3 4
**Beurteilung des Kausalzusammenhanges:**
O gesichert O wahrscheinlich O möglich O unwahrscheinlich O unbeurteilt O kein Zusammenhang

| Abklingen der Reaktion nach Absetzen des Medikamentes/der Medikamente? | Wiedereintreten der Reaktion nach erneuter Gabe |
|---|---|
| O Ja O Nein O unbekannt | O Ja O Nein O unbekannt |

### IV. BEGLEITMEDIKAMENTE, VORGESCHICHTE, WEITERE DATEN

**Begleitmedikamente mit Applikationsdaten** (excl. Medikamente zur Behandlung der Nebenwirkung)

| Anamnese/weitere Erläuterungen (z.B. Allergien, Schwangerschaft), Begleiterkrankungen | Allergien: O<br>Alkohol: O<br>Nikotin: O |
|---|---|

| Arzt/Apotheker Telefon | Adresse/Stempel |
|---|---|
| Datum/Unterschrift | |
| Meldung an weitere Stellen:<br>O BfArM O Andere Stellen:<br>O Arzneimittelkommission | |

## Appendix P

**BERICHT ÜBER UNERWÜNSCHTE ARZNEIMITTELWIRKUNGEN** (auch Verdachtsfälle)
Bundesinstitut für Arzneimittel und Medizinprodukte, Kurt-Georg-Kiesinger-Allee 3, 53175 Bonn, Tel.: 0228/207-30, FAX: 0228/207-5207

**BfArM**

| Firmen Code Nr. | Pat. Init. | Geburtsdatum | Geschlecht | Größe | Gewicht | Schwangerschafts-woche: |
|---|---|---|---|---|---|---|
| | N-name ☐ V-name ☐ | | m ☐ w ☐ | | | |

**Beobachtete unerwünschte Wirkungen** aufgetreten am Dauer

| Arzneimittel / Darreichungsform | Tages-dosis | Appli-kation | gegeben von / bis | wegen (Indikation) |
|---|---|---|---|---|
| 1. Chrg.-Nr: | | | | |
| 2. Chrg.-Nr: | | | | |
| 3. Chrg.-Nr: | | | | |
| 4. Chrg.-Nr: | | | | |

| Vermuteter Zusammenhang mit Arzneimittel Nr. 1 2 3 4 | dieses früher gegeben ja ☐ nein ☐ | vertragen ja ☐ nein ☐ | ggf. Reexposition neg. ☐ pos. ☐ |
|---|---|---|---|

**Grunderkrankung:** Begleiterkrankungen:

**Anamn. Besonderheiten:** Nikotin ☐ Alkohol ☐ Kontrazeptiva ☐ Schrittmacher ☐
Implantate ☐ Strahlentherapie ☐ physikal. Therapie ☐ Diät ☐ Allergien* ☐
Stoffwechseldefekte ☐ Arzneimittelabusus* ☐ Sonstige: ☐
weitere Erläuterungen

**Veränderung von Laborparametern in Zusammenhang mit der unerwünschten Arzneimittelwirkung:** (ggf. Befund beifügen)

**Verlauf der Therapie der unerwünschten Arzneimittelwirkung:** lebensbedrohend ja ☐ nein ☐

**Ausgang der unerwünschten Arzneimittelwirkung:**
wiederhergestellt ☐ bleibender Schaden ☐ noch nicht wiederhergestellt ☐ unbekannt ☐
Exitus ☐ Sektion ja ☐ nein ☐ (ggf. Befund beifügen)
Todesursache:

a) beh. Arzt
b) Hersteller
c) Arznei. Komm.

**Beurteilung des Kausalzusammenhanges:** gesichert ☐ wahrscheinlich ☐ möglich ☐ unwahrscheinlich ☐ unbeurteilt ☐ nicht zu beurteilen ☐

Weitere Bemerkungen:
(ggf. Anlage verwenden)

Wer wurde informiert: BfArM ☐ Hersteller ☐ Arznm.-Komm.-Ärzte ☐ Sonstige:

| Name des Arztes: Fachrichtung: PLZ: Klinik: ja ☐ nein ☐ (ggf. Stempel) | Hersteller: | Datum: Unterschrift: |
|---|---|---|

BfArM 643 / I (10.99)

**Appendix Q**

# Synopsis of Study Protocol

| | |
|---|---|
| **title** | **Regeneration in Acute Ischemic Stroke (RAIS) -**<br>**Stem Cell Based Stroke Therapy Program Regensburg**<br>**An Investigator-initiated clinical Phase I/II-Study** |
| **short title** | **RAIS** |
| **study population** | moderate acute stroke within MCA (middle cerebral artery) territory, specified as M2-occlusion, verified by CCT and/or MRI investigations |
| **studydesign** | • prospective, single arm, dose escalation, phase I/II clinical trial in acute stroke patients<br>• blinded evaluation by an independant study review comittee<br>• clinical evaluation (NIH-Stroke Scale) at days 1 through 7, at day 14, 28 and at 3 months<br>• assessment of quality of life and functional outcome (SSQL, Barthel, Rankin and FIM)<br>• neuroradiological evaluation of brain morphology (incl. T1 MP-Rage, DWI, ADC, TOF-MRA,Perfusion)<br>• daily clinical chemistry during days 1-7, 14, 28 and 90<br>• CD34/AC133 determination<br>• neuroradiological exams at day 1 (CCT or MRI, for inclusion), day 7 and at 3 months (MRI) |
| **Number of patients** | 15 patients (3 x 5 pts) |
| **Duration of treatment** | 5 daily s. c. applications of G-CSF (Neupogen® ) in each patient (dose escalation: initially in 5 pts. 2,5µg / kg BW, further in 5 pts. 5 µg / kg BW and in in 5 pts. 10 µg/ kg BW) starting from day 1 to day 5 in acute stroke |
| **Time of Study** | Recruitment phase: appr. 6 months<br>Observation period : 3 months in each patient |
| **End Points of Study** | **Primary Endpoints:**<br>• Toxicity and tolerability of G-CSF in acute stroke patients<br>**Secondary Endpoints:**<br>• clinical outcome in stroke patients after G-CSF-treatment as detected by NIH-Stroke Scale, BI, RS and FIM at day 7, day 14, 28 and at 3 months after stroke<br>• morphological changes in MRI at day 1-3, day 7 and at 3 months<br>• changes in brain perfusion as detected by MR-perfusion imaging at day 1-3, day 7 and at 3 months<br>• changes in quality of life (SSQL) at 3 months |
| **Study medication and treatment plan** | • Study medication: G-CSF (Neupogen R) 2,5 - 5 or 10 µg/kg BW s.c. days 1 - 5 in 5 days; in the highest dose at 10 µg/kg BW s.c. day 1 and 2 with 5 µg/kg BW s.c. and day 3, 4, 5 with 10µg/kg BW s.c.<br>• no other experimental medication, standard stroke treatment<br>• low molecular weight heparin s.c. (prevention of thromboembolism)<br>• aspirin 300 mg/d or clopidogrel 75 mg/d or dipyridamol 400 mg/d + aspirin 50 mg/d for secondary stroke prevention<br>• symptomatic therapy of blood pressure, hypercholesterolemia, infection etc. |

| | |
|---|---|
| | • physiotherapy |
| **Time points of examination** | • at time of inclusion into study protocol<br>• daily during treatment phase (day 1 - day 5) clinical exams, further on at days 6,7, 14, 28 and at 3 months (complete clinical examination, neurological examination with NIH-Stroke Scale)<br>• daily clinical chemistry during treatment phase, days 6, 7, 14 and 28 (specified below)<br>• Assessment of quality of life (SSQL)<br>• MRI imaging at days 1-3, day 7 and at 3 months |

| | |
|---|---|
| **Inclusion criteria** | • moderate acute stroke within MCA (middle cerebral artery) territory, specified as M2-occlusion<br>• dominant and non-dominant hemisphere<br>• patient must comprehend study protocol (minor aphasia accepted)<br>• inclusion into study protocol within 12 hours after stroke<br>• age ≥ 18 years, ≤ 65 years<br>• NIH-stroke scale 4-22<br>• adequate liver function: GOT, GPT, γGT < 3 times upper normal values; bilirubin < 1,5 mg/dl<br>• adequate bone marrow function (no gross abnormalities in thrombocytes or leukocytes)<br>• patients must be compliant<br>• patient must be able to give fully written informed consent |
| **Exclusion criteria** | • previous treatment with Abciximab (Rheopro $^{R}$)<br>• global aphasia<br>• any type of immediately necessary intervention, as e.g. carotid endarterectomy<br>• ulcerating plaque of carotid artery<br>• signs and symptoms of acute cerebral vasculitis<br>• dissection of brain arteries relevant for acute symptomatology<br>• thrombolytic therapy within previous 2 weeks, however an acute preceding thrombolysis is allowed<br>• previous disabling stroke events (ischemic or hemorrhagic stroke)<br>• systemic malignancy<br>• hematologic systemic disorder (e.g. myeloproliferative disorder)<br>• thrombocyte function disorders<br>• metabolic syndrome with inadequate treatment parameters, e.g. excessive hypertension, hyperlipemia, hyperglycemia<br>• known deficite in hemostasis<br>• any other serious disease, as for example:<br>    • severe psychiatric disorder (major depression, schizophrenic psychosis)<br>    • severe cardiac disorder (with hemodynamic relevance)<br>    • severe CAD<br>    • chronic hepatitis<br>    • positive HIV-serology<br>• allergy against G-CSF (Neupogen $^{R}$), E-coli-products<br>• pregnancy (test will be performed in case of uncertainty)<br>• heavy smoker, daily use ≥ 20 cigarettes |
| **Criteria for toxicity - adverse events** | • continuous fever > 38.5 $^{0}$C<br>• blood thrombocytes > 500.000 /µl with clinical signs and symptoms<br>• leukocytosis > 20.000 /µl will lead to 50% dose-reduction of G-CSF<br>• severe continuos leukocytosis > 50.000 /µl with clinical signs and symptoms |

| | |
|---|---|
| | • any type of central nervous system adverse events not regularly seen with stroke of MCA type<br>• recurrent minor or major stroke within 14 days in study, unexplained by individual patient`s pathophysiology<br>• macroscopic CNS hemorrhage within 14 days after study inclusion<br>• any type of systemic toxicity unexplained by previous toxicity studies<br>• CAD previously unknown |
| **Criteria for study interruption** | The **entire study** must be interrupted in case of 3 serious adverse events or death within the first 5 patients, or in the other two cohorts<br>The study must be interrupted for the **individual patient** in case of:<br>• severe toxicity<br>• severe continuos leukocytosis > 70.000 /µl with clinical signs and symptoms<br>• second stroke or major hemorrhage within treatment period<br>• major side effects due to known side effects of Neupogen R<br>• allergies against Neupogen R<br>• wish of patient to terminate study |
| **Data acquisition** | **At time of inclusion (Screening, Baseline)**<br>• clinical findings (complete physical, neurological examination)<br>• complete patient`s medical history<br>• documented neurological deficit (Rankin and NIH-Stroke Scales)<br>• cranial computed tomography (CCT/CTA) or magnetic resonance imaging (MRI/MRA) indicative of MCA (M2) - infarction (T1, T2, DW, ADC)<br>• Duplex sonography of external brain arteries to exclude ulcerous plaques and / or dissection<br>• Clinical Chemistry: differential blood count, CRP, elektrolytes (Na+, K+, Ca++), creatinin, urea, liver enzymes (bilirubin, AP, GGT, GPT, GOT), CK, glucose, total cholesterine, triglyceride, HDL, LDL, INR, PTT, HIV-serology, TT and PTT, INR, CD 34, AC 133<br>• electrocardiogramm<br>• evaluation of SSQL<br>• women < 50 years: pregnancy test<br>• body temperature<br>• informed consent of patient (either written or testified by an independant witness<br>**day 2 / 3**<br>• MRI in case CCT was performed at inclusion<br>**day 2, 3,4, 5, 6, 7, 14, 28, 90 and at interruption of study (primary endpoint)**<br>• clinical findings (complete physical, neurological examination, temperature, RR, puls)<br>• documented neurological deficit (RS, BI and NIHSS)<br>• Clinical Chemistry: differential blood count, CRP, elektrolytes (Na+, K+, Ca++), creatinin, urea, liver enzymes (bilirubin, AP, GGT, GPT, GOT), CK, glucose, total cholesterine, triglyceride, HDL, LDL, INR, PTT, HIV-serology, TT and PTT, INR<br>• CD 34, AC 133 cells will be quantified and functionally characterized at day 0, day 4, day 7, day 28 and day 90<br>• Electrocardiogram<br>• quality of life (SSQL) at day 90 |

| | |
|---|---|
| | • MRI at day 7 and day 90 |
| **SSQL** | **stroke-specific scale for quality of life (SSQL) at day 90** |
| **MR-examinations** | **MP-Rage: T1-weighted 3-D-GE Sequence (day 1-3, day 7 and day 90)-**<br>axial Flair, axial DWI and ADC, coronar T1, axial Perfusion, TOF-MRA (day 2/3), in case significant volume changes will be observed, a further analysis with voxel-directed morphometry will be performed. |
| **interim analysis** | A formal interim analysis should be performed at completion of the first 8 patients |
| **final analysis of study** | after completion of patient number 15 all data will be evaluated by the different section study coordinators within 6 calender weeks, a publication draft will be written by the Principal investigator within 3 months after end of study. |
