# Supplementary material for: Mobilisation of Hematopoietic CD34+ Precursor Cells in Patients with Acute Stroke Is Safe - Results of an Open-Labeled Non Randomized Phase I/II Trial
Source: PLoS One. 2011 Aug 26;6(8):e23099. doi: 10.1371/journal.pone.0023099 (PMC3162562; doi:10.1371/journal.pone.0023099)
Supplement: Amendment S1 — Amendments to the original protocol. (PDF) [file pone.0023099.s006.pdf]

UNIVERSITÄT
REGENSBURG

KLINIKUM

Neurologische Universitätsklinik im BKR • 93042 Regensburg

Zentrum für Klinische Studien
Geschäftsstelle der Ethik-Kommission der Medizinischen Fakultät der Universität Regensburg

Z.Hd. Vorsitzender
Prof. Dr. med. R. Andreesen

Klinik und Poliklinik für Neurologie
im Bezirksklinikum
Direktor: Prof. Dr. med. Ulrich Bogdahn
Universitätsstraße 84
93053 Regensburg

Telefon: 0941 941-*3003* Fax: *-3015*

Regensburg, den 13.06.05

Betreff: RAIS-Studie (Nr 02/146)

Amendment für RAIS-Studie (Regeneration in Acute Ischemic Stroke) nach Behandlung von 15 Patienten mit G-CSF bei akutem Mediateilinfarkt

Sehr geehrter Herr Professor Andreesen,

die RAIS-Studie stellt als Phase II Studie eine „Safety"-Studie zur Anwendung von G-CSF (Granulocyte-Colony-Stimulating-Factor) bei akuter cerebraler Ischämie dar.

Sie basiert auf aus der Literatur bekannten Daten, dass „Granulocyte-Colony-Stimulating Factor" (G-CSF) einen neuroprotektive Eigenschaften besitzt und zur Mobilisation von unreifen Vorläuferzellen aus dem Knochenmark führt. Diese Vorläuferzellen können möglicherweise durch Transdifferenzierung, durch Zellfusion und/oder durch „trophic support" eine neuronale Regeneration und somit eine Funktionsbesserung in geschädigtem neuronalen Gewebe positiv beeinflussen. So wird Patienten <65 Jahre mit einem akut ischämischen cerebralen Ereignis (Mediateilinfarkt) im Rahmen der RAIS-Studie G-CSF (Neupogen®) appliziert.

Bis zum jetzigen Zeitpunkt sind insgesamt 15 Patienten im Alter von 32-65 Jahren (Altersdurchschnitt 54 Jahre) im Rahmen der Studie behandelt worden. Dabei haben insgesamt 3 Patienten eine Dosis von 1,25 µg / kg Körpergewicht G-CSF erhalten. 5 Patienten wurde eine Dosis von

Hausadresse:
Bezirksklinikum
Universitätsstraße 84
93053 Regensburg

Postadresse:
93042 Regensburg

eMail: ulrich.bogdahn@bkr-regensburg.de

Öffentl. Verkehrsmittel:
Buslinien 4,6,11

2,5 µg / kg Körpergewicht appliziert. Nach komplikationsloser Verträglichkeit dieser Dosierung wurde die Dosisreduktionsgrenze von 20.000 Leukozyten / µl, deren Überschreiten nach Studienprotokoll eine Dosisreduktion um 50% nach sich zog, auf 50.000 Leukozyten / µl angehoben. Nach Anheben dieser Dosisreduktionsgrenze konnten 6 weitere Patienten mit einer Dosierung von 5 µg / kg Körpergewicht behandelt werden. In dieser Kohorte kam es bei einem Patienten zu einem SAE. Bei dem Patienten war es initial zu einem Stammganglieninfarkt auf der rechten Seite gekommen. Bei V.a. auf eine kardio-embolische Genese erfolgte eine PTT-wirksame Heparinisierung, worunter es ca. 14 Stunden nach Erstsymptomen zu einer Einblutung in den Infarkt mit Ventrikeleinbruch kam, so dass eine Verlegung in die Neurochirurgie unseres Hauses notwendig wurde. Natürlich ist ein Zusammenhang zwischen G-CSF und dieser Einblutung nicht mit letzter Sicherheit auszuschliessen, jedoch sind Einblutungen in einen frischen cerebralen Infarkt eine bekannte Komplikation, zumal der Patient i.v. Heparin erhalten hatte. Die Einblutungsrate in einen frischen cerebralen Infarkt unter i.v.-Heparingabe wird nach Literaturangaben mit ca. 1% angegeben (1). Auch der zeitliche Abstand von nur 5 Stunden zwischen G-CSF-Gabe und Einblutung macht eine Kausalität mit G-CSF eher unwahrscheinlich. Eine erhöhte Blutungsneigung ist unter G-CSF bisher in der Literatur nicht beschrieben worden. Nach Meldung dieses SAE an die Ethik-Kommission (ZKS), BfArM und an die Herstellerfirma von G-CSF (Amgen) konnte die Studie nach Freigabe durch diese Stellen fortgeführt werden.

Ein Überschreiten der Grenze von 50.000 Leukozyten / µl wurde in dieser Kohorte nicht beobachtet. Nach Abschluss dieser Kohorte wurde ein Zwischenbericht (siehe Zwischenbericht vom 10.01.2005) erstellt und es wurde begonnen, Patienten für die 3. Kohorte, die eine Dosis von 5 µg / kg Körpergewicht an den Tagen d1 und d2 und nachfolgend eine Dosiseskalation auf 10 µg / kg Körpergewicht an den Tagen d3, d4 und d5 vorsieht, zu rekrutieren. Bei dem ersten Patienten in dieser Kohorte kam es zu einem Anstieg der Leukozyten bis auf 75.000 / µl. Damit wurde die Leukozytengrenze von 50.000 / µl überschritten, so dass die Dosis, wie im Protokoll vorgesehen, wieder auf 5 µg / kg halbiert werden musste.

Aufgrund des bisher komplikationslosen Verlaufes der Dosiseskalation beantragen wir mit diesem Schreiben ein Anheben der Dosisreduktionsgrenze von 50.000 Leukozyten / µl auf 80.000 Leukozyten für die 3. Kohorte mit einer Maximaldosierung von 10 µg / kg Körpergewicht. Kom-

plikationen durch erhöhte Leukozytenzahlen wurden bisher nicht beobachtet, so dass wir eine neue Dosisreduktionsgrenze von 80.000 Leukozyten / µl für vertretbar halten. Die Dosierung von 10 µg / kg Körpergewicht ist eine Standard-Dosierung und wird zur Mobilisierung von CD34+-Zellen bei Stammzellspendern eingesetzt (2). In dieser Dosierung werden Leukozytenzahlen > 50.000 / µl beobachtet. Rheologische Komplikationen wurden auch bei dieser Dosierung nicht beobachtet (3). Auch Bensinger et al. testeten die Auswirkungen von G-CSF bei gesunden Patienten. Hier wurden Leukozytenzahlen bis 80.000 / µl ohne schwerwiegende Nebenwirkungen toleriert (4).

Nachdem auch wir bei Patienten mit akutem ischämischen cerebralen Ereignis Leukozytenwerte bis 75.000 / µl keine Komplikationen beobachtet haben, halten wir eine Anhebung der Dosisreduktionsgrenze auf 80.000 / µl für sicher und vertretbar.

Sollten inhaltliche oder regulatorische Fragen noch offen oder nicht hinreichend geklärt sein, teilen Sie dies uns zeitnah mit. Wir werden uns dann bemühen, diese umgehend genauer zu beantworten.

Wir hoffen auf ein positives Votum und verbleiben mit freundlichen Grüßen

Prof. Dr. med. U. Bogdahn
(Direktor der Klinik)

Dr. med. S. Haas
(Assistenzarzt)

1) Sherman DG Heparin and heparinoids in stroke. Neurology. 1998 Sep;51(3 Suppl 3):S56-8

2) M. Engelhardt, W. Aulitzky Leitlinien: Empfehlungen der DGHO für die Diagnostik und Therapie hämatologischer und onkologischer Erkrankungen / Januar 05

3) Grigg AP, Roberts AW, Raunow H, Houghton S, Layton JE, Boyd AW, McGrath KM, Maher D Optimizing dose and scheduling of filgrastim (granulocyte colony-stimulating factor) for mobilization and collection of peripheral blood progenitor cells in normal volunteers Blood. 1995 Dec 15;86(12):4437-45

4) Bensinger WI, Price TH, Dale DC, Appelbaum FR, Clift R, Lilleby K, Williams B, Storb R, Thomas ED, Buckner CD The effects of daily recombinant human granulocyte colony-stimulating factor administration on normal granulocyte donors undergoing leukapheresis Blood. 1993 Apr 1;81(7):1883-8
